# Supplementary material for: Thalamic control of sensory processing and spindles in a biophysical somatosensory thalamoreticular circuit model of wakefulness and sleep
Source: Cell Rep. 2023 Mar 1;42(3):112200. doi: 10.1016/j.celrep.2023.112200 (PMC10066598; doi:10.1016/j.celrep.2023.112200)
Supplement: Document S1. Tables S1–S3 and Figures S1–S5 [file mmc1.pdf]

Cell Reports, Volume *42*

Supplemental information

Thalamic control of sensory processing and spindles in a biophysical somatosensory thalamoreticular circuit model of wakefulness and sleep

Elisabetta Iavarone, Jane Simko, Ying Shi, Marine Bertschy, María García-Amado, Polina Litvak, Anna-Kristin Kaufmann, Christian O'Reilly, Oren Amsalem, Marwan Abdellah, Grigori Chevtchenko, Benoît Coste, Jean-Denis Courcol, András Ecker, Cyrille Favreau, Adrien Christian Fleury, Werner Van Geit, Michael Gevaert, Nadir Román Guerrero, Joni Herttuainen, Genrich Ivaska, Samuel Kerrien, James G. King, Pramod Kumbhar, Patrycja Lurie, Ioannis Magkanaris, Vignayanandam Ravindernath Muddapu, Jayakrishnan Nair, Fernando L. Pereira, Rodrigo Perin, Fabien Petitjean, Rajnish Ranjan, Michael Reimann, Liviu Soltuzu, Mohameth François Sy, M. Anıl Tuncel, Alexander Ulbrich, Matthias Wolf, Francisco Clascá, Henry Markram, and Sean L. Hill

Table S1

Pathway	Synapse type	g_{syn} (nS)	τ_d (ms)	NMDA/AMPA ratio	U_{SE}	D	F
**Rt_RC to Rt_RC**	Inh. Dep.	0.9±0.23	8.3±2.2	NA	0.41±0.14	464±339	54±71
**Rt_RC to VPL_TC**	Inh. Dep.	1.1±0.4	8.3±2.2	NA	0.32±0.18	352±46	2±209
**Rt_RC to VPL_IN**	Inh. Dep.	0.9±0.23	8.3±2.2	NA	0.41±0.14	464±339	54±71
**VPL_TC to Rt_RC**	Exc. Dep.	2.8±0.1	1.58±0.26	0.57	0.86±0.09	671±17	17±5
**VPL_IN to VPL_TC**	Inh. Dep.	0.4±0.4	8.3±2.2	NA	0.47±0.18	137±46	239±209
**VPL_IN to VPL_IN**	Inh. Dep.	2.7±0.4	8.3±2.2	NA	0.41±0.14	464±339	54±71
**ML to VPL_TC**	Exc. Dep.	1.15±0.12	1.74±0.18	0.41	0.3±0.21	2350±315	1±2
**ML to VPL_IN**	Exc. Dep.	1.15±0.12	1.74±0.18	0.41	0.48±0.21	690±315	57±53
**CT to Rt_RC**	Exc. Fac.	0.16±0.016	2.74±0.25	0.99	0.09±0.12	138±211	670±830
**CT to VPL_TC**	Exc. Fac.	0.16±0.016	1.74±0.18	1.91	0.09±0.12	138±211	670±830
**CT to VPL_IN**	Exc. Fac.	0.16±0.016	1.74±0.18	0.99	0.09±0.12	138±211	670±830

Table S1. Synapse kinetics and short-term plasticity parameters, related to Figure 2

Synaptic parameters for all pathways in the model. Quantal synaptic conductance g_{syn} (in nanosiemens nS), τ_d is the decay time constant of AMPA and $GABA_A$ currents for excitatory and inhibitory connections. U_{SE} (utilisation of synaptic efficacy, analogous to release probability), D (time constant of recovery from depression), F (time constant of recovery from facilitation) are the short-term plasticity parameters. Values are expressed as mean ± standard deviation. All the parameters were fitted to in-house paired-recordings or generalized from similar pathways.

Table S2

Presynaptic	Postsynaptic	CV 1st PSP amplitude, experiment (mV)	CV 1st PSP amplitude, model (mV)	Data source
**Rt_RC**	**VPL_TC**	0.4600 (n=1)	0.8424 ± 0.3450 (n=47)	In-house
**VPL_TC**	**Rt_RC**	0.1232 ± 0.0686 (n=11)	0.3089 ± 0.2112 (n=43)	(Gentet and Ulrich, 2003)
**VPL_IN**	**VPL_TC**	0.5479 ± 0.1744 (n=4)	0.8663 ± 0.386 (n=22)	In-house
**VPL_IN**	**VPL_IN**	0.5028 ± 0.2783 (n=10)	1.0993 ± 0.4132 (n=49)	In-house
**ML**	**VPL_IN**	0.5466 ± 0.1195 (n=1)	1.4047 ± 0.7389 (n=49)	In-house

TableS 2. Coefficient of variation (CV) of first PSP amplitudes, related to Figure 2

CV of first PSP amplitudes values as characterized experimentally through *in vitro* paired recordings. Values are reported as mean ± standard deviation (of multiple pairs). (Related to Fig. 5C1).

Table S3

Presynaptic	Postsynaptic	PSP amplitude, experiment (mV)	PSP amplitude, model (mV)	Data source
**Rt_RC**	**VPL_TC**	1.33 ± 0.36 (n=1)	1.31 ± 1.30 (n=47)	In-house
**VPL_TC**	**Rt_RC**	7.4 ± 1.5 (n=11)	6.79 ± 1.30 (n=43)	(Gentet and Ulrich, 2003)
**VPL_IN**	**VPL_TC**	0.55±0.15 (n=4)	1.16 ± 1.56 (n=22)	In-house
**VPL_IN**	**VPL_IN**	1.66±1.44 (n=10)	0.82 ± 0.77 (n=49)	In-house
**ML**	**VPL_TC**	4.58 ± 0.30 (n=11)	3.62 ± 2.30 (n=49)	(Mo et al., 2017)
**CT**	**VPL_TC**	0.085±0.008 (n=3)	0.071 ± 0.022 (n=29)	(Golshani et al., 2001)

Table S3. Postsynaptic potential (PSP) amplitudes, related to Figure 2

PSP amplitude values as characterized experimentally through *in vitro* paired recordings. Values are reported as mean ± standard deviation (of multiple pairs).

Figure S1

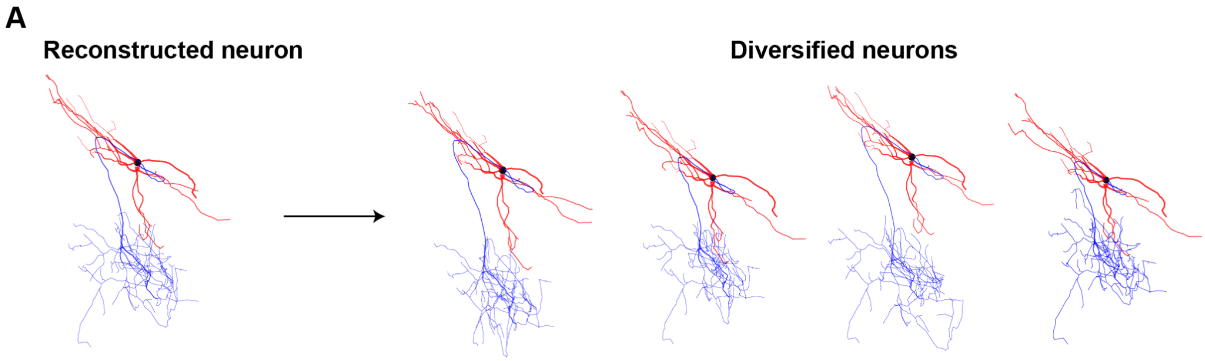

Figure S1, morphology diversification, related to Figure 1.

(A) The reconstructed neuron on the left was diversified to generate a sample of unique morphologies, by introducing variability (jittering in f branch lengths of 0 ± 20% and jittering in branch rotations of 0° ± 20°, mean ± standard deviation, see Methods) to the branch lengths and angles (see Methods).

Figure S2

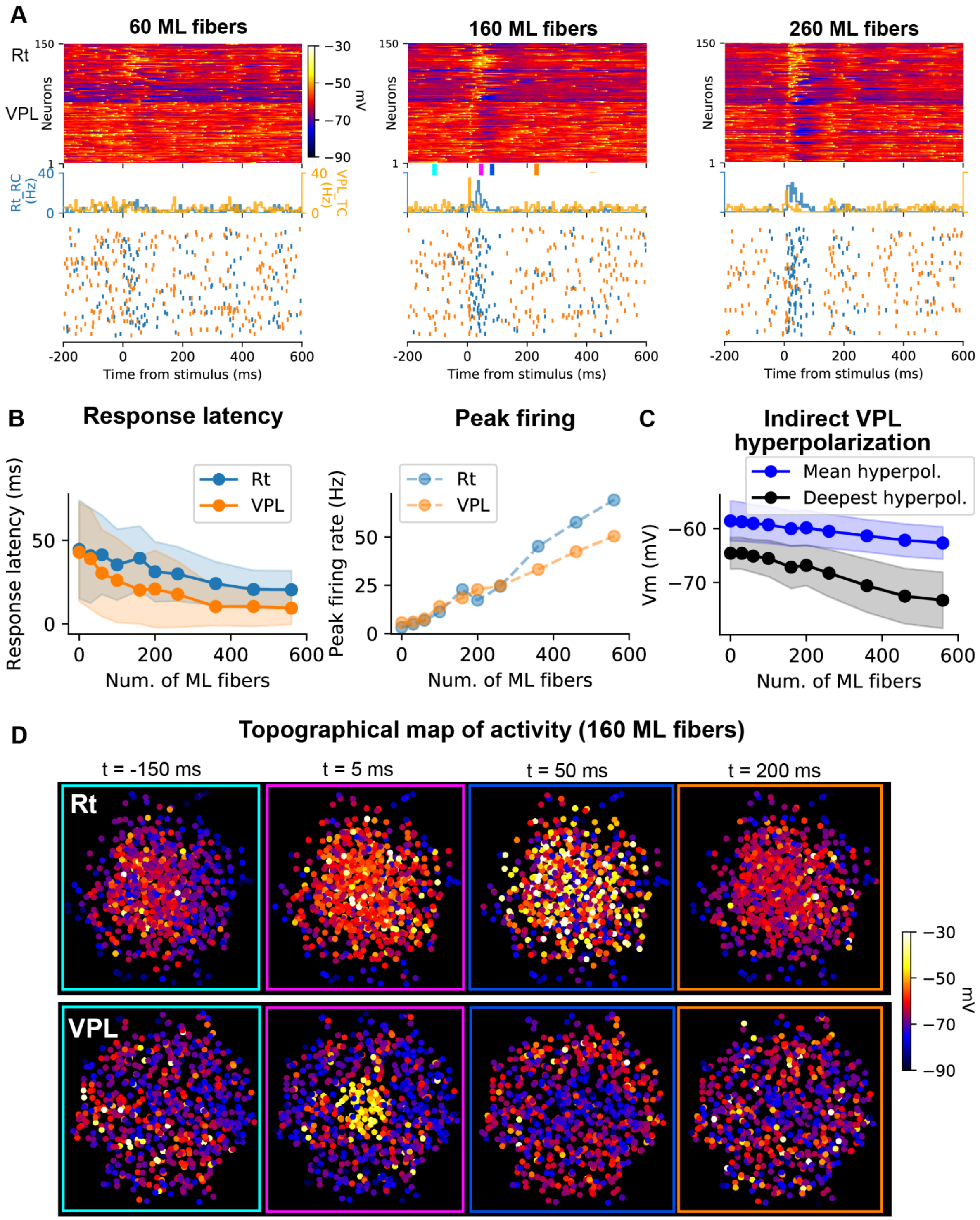
A
60 ML fibers
160 ML fibers
260 ML fibers
Rt
VPL
Neurons
mV
Rt_RC (Hz)
VPL_TC (Hz)
Time from stimulus (ms)
B
Response latency
Response latency (ms)
Num. of ML fibers
Peak firing
Peak firing rate (Hz)
C
Indirect VPL hyperpolarization
Mean hyperpol.
Deepest hyperpol.
Vm (mV)
D
Topographical map of activity (160 ML fibers)
t = -150 ms
t = 5 ms
t = 50 ms
t = 200 ms

Figure S2. **Stimulus-dependent recruitment of reticular nucleus neurons and surround inhibition of thalamic neurons (*in vivo* wakefulness-like condition), related to Figure 4.**

(**A**) Simulated sensory inputs with brief activation of increasing numbers of medial lemniscal fibers (ML). Top: voltage rasters show Rt and VPL responses of a sample of 150 active neurons, sorted by their vertical position in the microcircuit. Bottom: spiking responses (firing rate histograms and spike rasters). Note that stimulus-evoked responses in the VPL as well as the following hyperpolarization increase with increasing stimulus size. (**B**) Stimulus-response curves. Response latency decreases with stimulus size (left), while peak firing rates increase the VPL, as well as in the Rt (right). Mean (lines) and standard deviation (shaded areas) are shown. The peak firing rate is calculated in the 100 ms following the stimulus and response latency as the time to first spike after the stimulus (n=1,000 neurons). (**C**) Stimulus-dependent hyperpolarization in the VPL. The mean and deepest hyperpolarization in VPL cells are shown (lines: mean, shaded areas: standard deviation). Note that with increasing stimulus size more VPL cells are inhibited by Rt neurons and the hyperpolarization becomes stronger. The hyperpolarization is calculated in a time window of 40-200 ms after the stimulus (same sample as in B, n=1,000). (**D**) Topographical activity in a slice through the VPL and Rt showing the average membrane potentials at different time windows before, during and after the stimulus, as indicated by colored ticks in A (middle panel), 160 fibers were activated. Time windows of 10 ms starting at the time indicated were used for the average activity. Note that increased activity is confined to the central part of the VPL in response to the stimulus (t=5 ms), which triggers spiking activity in the Rt (t=50 ms) in central as well as in peripheral neurons. This result suggests that the Rt has larger receptive fields compared to the VPL. Consequently (t=50ms), the central part and the surround in the VPL is inhibited (blue points at t=50ms).

Figure S3

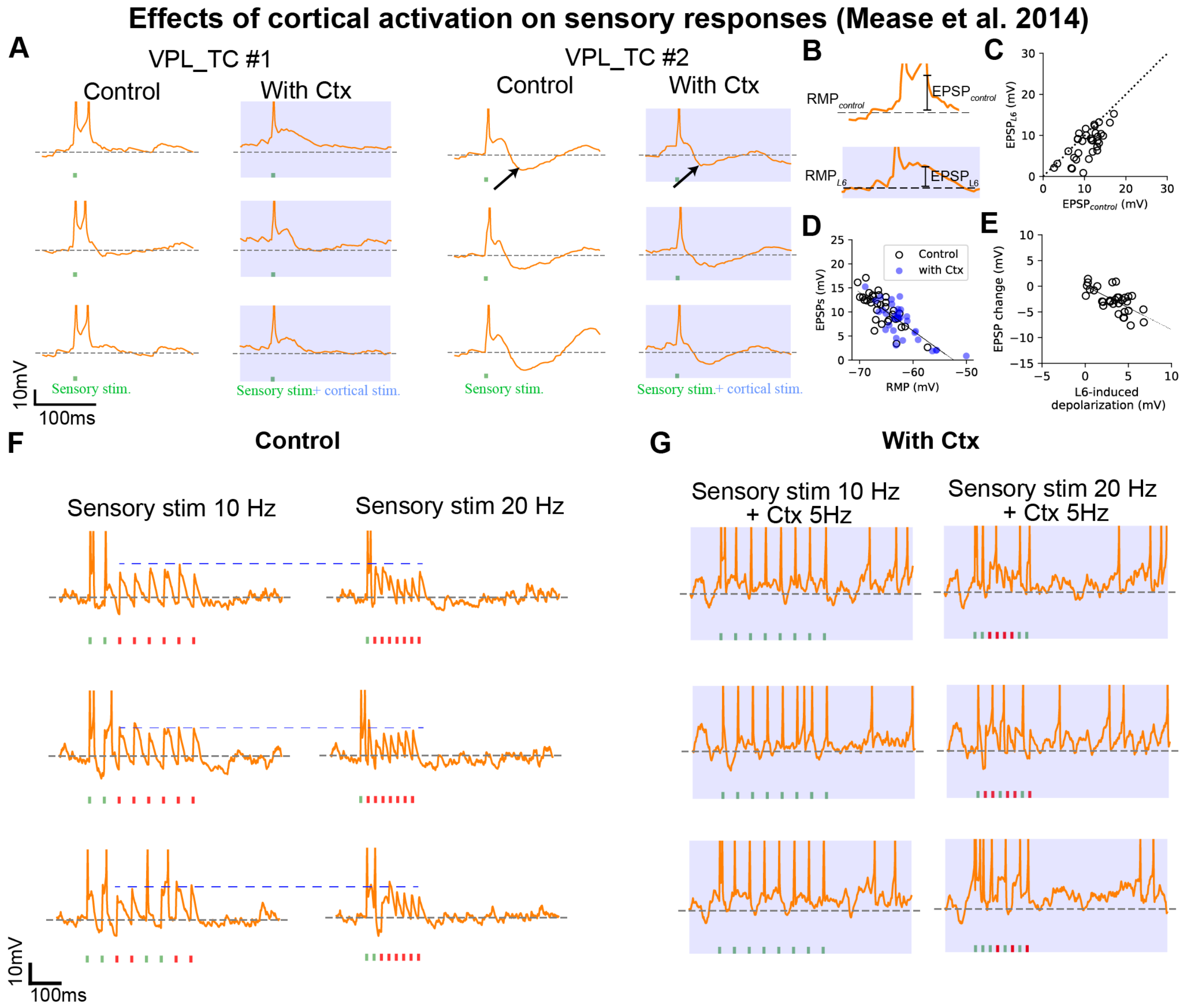

Effects of cortical activation on sensory responses (Mease et al. 2014)
A
VPL_TC #1
Control
With Ctx
VPL_TC #2
Control
With Ctx
Sensory stim.
Sensory stim.+ cortical stim.
Sensory stim.
Sensory stim.+ cortical stim.
10mV
100ms
B
RMP control
EPSP control
RMP L6
EPSP L6
C
EPSP L6 (mV)
EPSP control (mV)
D
Control
with Ctx
EPSPs (mV)
RMP (mV)
E
EPSP change (mV)
L6-induced depolarization (mV)
F
Control
Sensory stim 10 Hz
Sensory stim 20 Hz
G
With Ctx
Sensory stim 10 Hz + Ctx 5Hz
Sensory stim 20 Hz + Ctx 5Hz
10mV
100ms

Figure S3. Cortical activation decreases sensory adaptation by depolarizing VPL_TCs and enhances responses to stimuli at ~10Hz preferentially, related to Figure 5.

(**A**) Left: Single cell recording of a VPL_TC neuron (3 of 25 repetitions are shown) that responded to the sensory stimulus (green) with a burst of two spikes. The sensory stimulus was generated with brief synchronous activation of 160 ML afferent fibers. Right: activation of the CT afferent fibers (blue) stimulus depolarized the cells and shifted their responses to single spikes. VPL_TC #2 showed a marked IPSP (arrow) following the stimulus-evoked spike, which was reduced with cortical activation. CT fibers were activated with noisy input at 4 Hz, 200 ms before the sensory stimulus to approximate the optogenetic protocol in Mease et al., 2014. (**B**) Illustration of different metrics used to quantify subthreshold responses (in a time window of 50 ms after the stimulus to the sensory stimuli (cfr. Mease et al, 2014). (**C**) Population analysis of VPL_TC cells (n=50, values are median of the 25 repetitions for each cell) showing the decrease of EPSP amplitude with cortical activation ($EPSP_{L6}$). This effect is due to partial inactivation of the low-threshold Ca^{2+} conductance, but inhibition from the Rt can't be excluded. (**D**) The amplitude of the EPSPs (both with and without cortical activation) is negatively correlated with the resting potential of the cell ($r=-0.8$). This is due to a greater availability of ionic currents activated at hyperpolarized potentials and greater driving force of excitatory conductances (whose reversal potential is 0 mV). (**E**) Correlation between the magnitude of sensory response change ($EPSP_{L6}$ − EPSP with sensory stimulus only) and the depolarization induced by the cortical activation. The line shows the best fit ($r=-0.8$). This shows that greater cortical activation corresponds to decreased responses to sensory input (for single stimulation). (**F**) Single cell recordings of a VPL_TC neuron (3 of 25 repetitions are shown) with sensory stimulus at 10 Hz (left) and 20 Hz (right). Note the smaller amplitudes of the EPSPs in response to the 20 Hz stimulus. (**G**) Same cell as in F, with activation of cortex (noisy input at 5 Hz). Note a higher number of spiking failures (red ticks) with 20 Hz sensory stimulation. With 10 Hz stimulus, cortical activation made the cell fire in response to each pulse of the stimulus (green ticks).

Figure S4

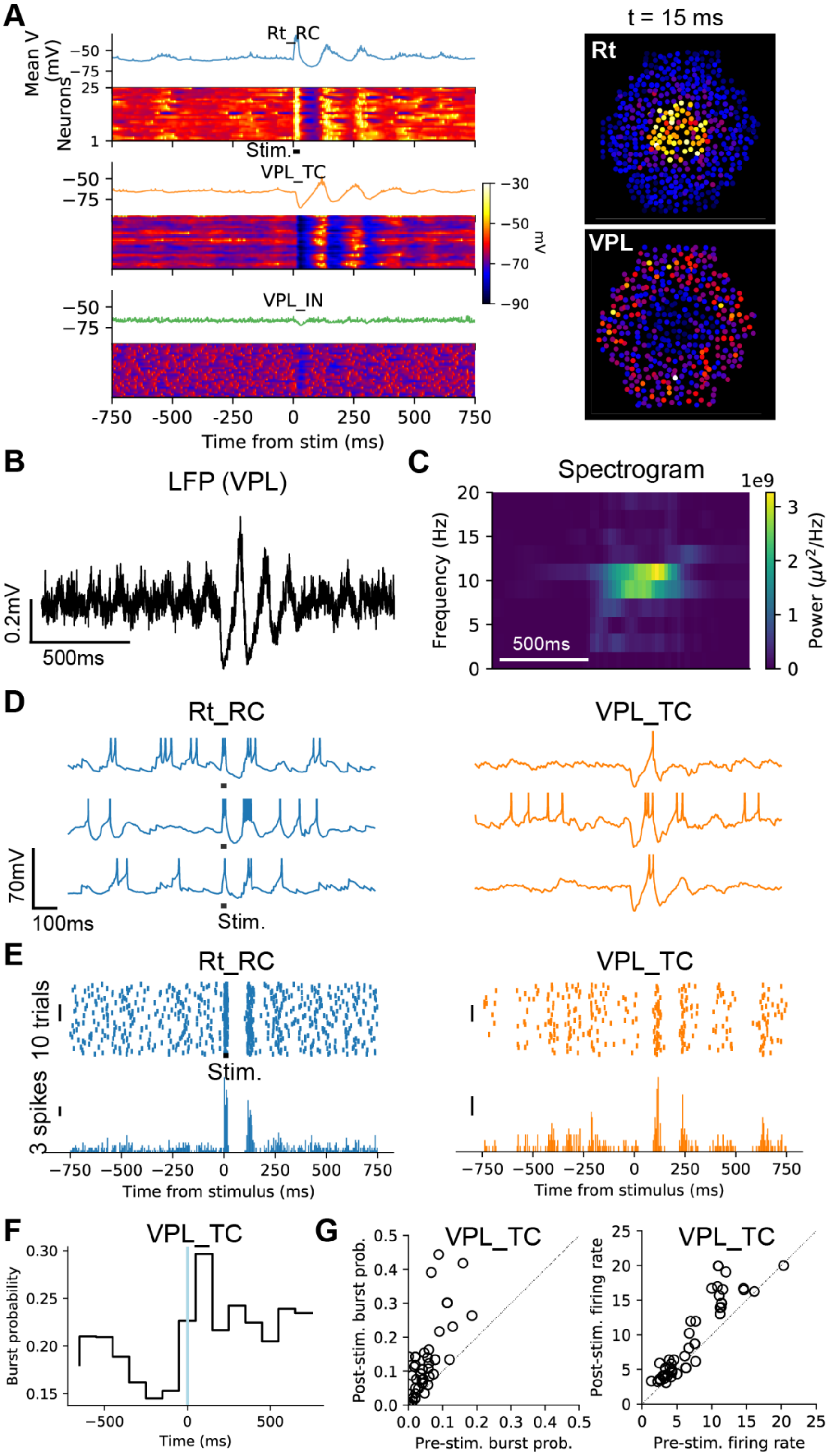
A
Rt_RC
Mean V (mV)
Neurons
Stim.
VPL_TC
VPL_IN
mV
Time from stim (ms)
t = 15 ms
Rt
VPL
B
LFP (VPL)
0.2mV
500ms
C
Spectrogram
Frequency (Hz)
Power (μV^2/Hz)
1e9
500ms
D
Rt_RC
VPL_TC
70mV
100ms
Stim.
E
Rt_RC
VPL_TC
10 trials
3 spikes
Stim.
Time from stimulus (ms)
Time from stimulus (ms)
F
VPL_TC
Burst probability
Time (ms)
G
VPL_TC
Post-stim. burst prob.
Pre-stim. burst prob.
VPL_TC
Post-stim. firing rate
Pre-stim. firing rate

Figure S4. Activating the reticular nucleus increases thalamic bursts and initiates spindle-like oscillations (*in vivo* wakefulness-like condition), related to Figure 6.

Spindle-like oscillations are evoked by localized pulse (20 ms) activation of 750 Rt_RC cells located at the center of the microcircuit. (**A**) Left: voltage rasters showing spindle-like activity. A sample of neighboring 25 neurons per each m-type is shown and color-coded according to their membrane potential. Right: topographical map of activity showing average membrane potential of Rt and VPL neurons in a 10 ms time window starting at the time indicated. (**B**) LFP recording from a central site in the VPL. Note the increased oscillatory activity after the stimulus applied to the Rt (not shown here). (**C**) Frequency-time analysis of the LFP in C showing increased power in the 8-10 Hz frequency range. (**D**) Example of single cell recordings for 3 cells per m-type. Note the burst responses in Rt_RCs and the IPSPs-rebound sequences in VPL_TCs. (**E**) Left: spike rasters and PSTHs showing the activity of one exemplar Rt_RC neuron (50 trials). Note the increased activity in response to the stimulus (black dot, 20 ms pulse) and a second peak, generated by network interactions. Right: Same as in A, for one example VPL_TC, note the post-inhibitory rebound response ~100 ms after the Rt stimulation. (**F**) Histogram showing increased burst probability following the stimulus in VPL_TC (n=100 VPL_TC), as shown in experiments (Halassa et al., 2011). (**G**) Left: burst probability in VPL_TCs increases as a result of Rt_RC stimulus (each dot corresponds to one cell, the same sample as in F, n=100). Right: analysis of firing rates of VPL_TCs before and after the stimulus, as shown in experiments (Halassa et al., 2011). Pre-stim./post-stim. data were calculated in the 1s preceding/following the stimulus

Figure S5

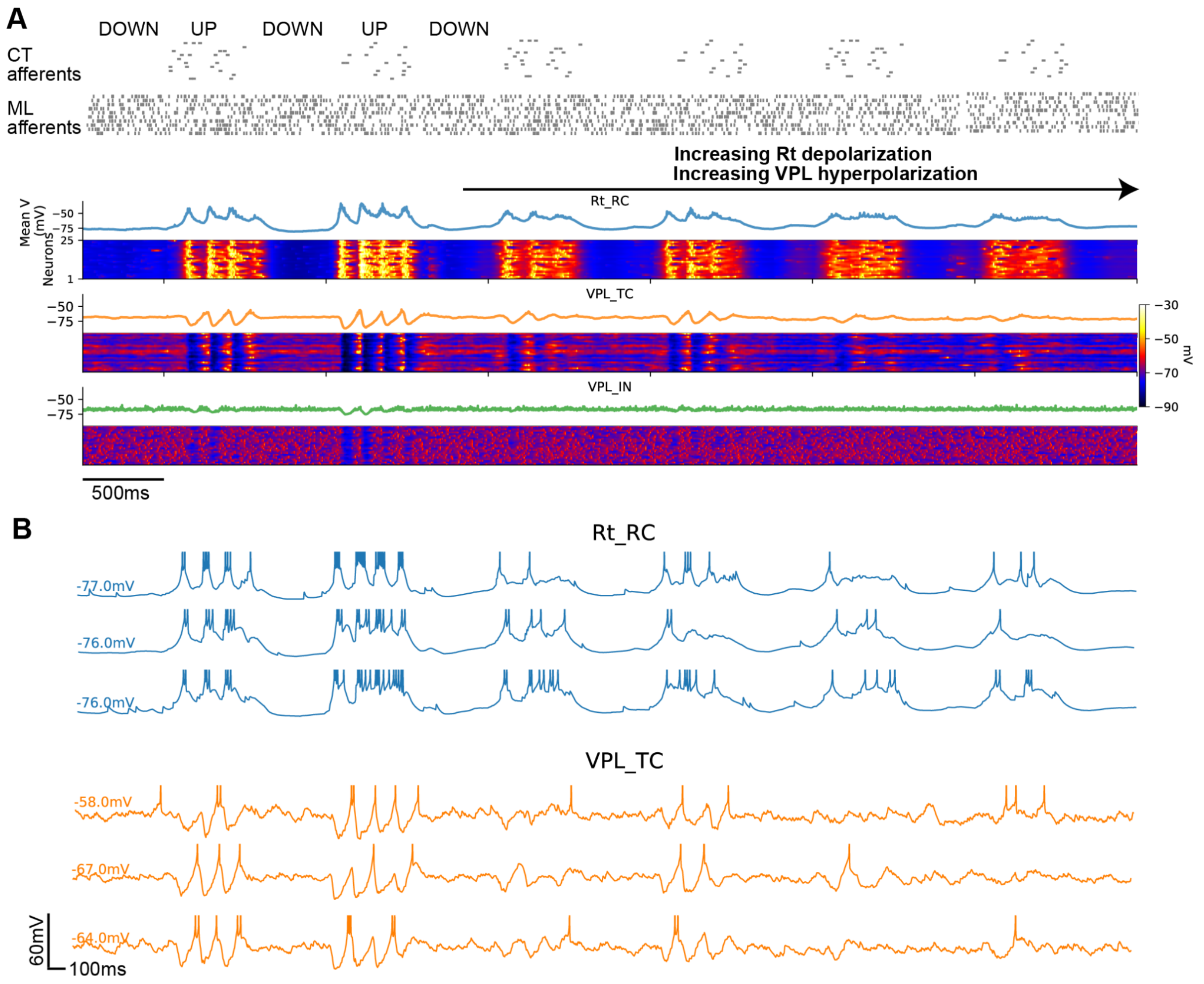

Figure S5. Spindle-like oscillations cease when simulating the effect of neuromodulation on thalamic and reticular neurons, related to Figure 7.

(**A**) Simulated UP and DOWN states evoke reticular and thalamic depolarizations through afferent input, resulting in the "ping-pong" generation of spindle-like oscillations (as seen in Fig. 11). To approximate the differential effects of neuromodulators (e.g. acetylcholine) onto Rt_RC and VPL_TC we applied constant currents to depolarize Rt_RC and hyperpolarize VPL_TC cells. This resulted in spindle-like oscillations (left) being abolished (right). (**B**) Example single cell recordings from the simulation in A, note that while Rt_RC cells fire preferentially low threshold bursts during the cortical UP states (left), they transition to single spike modes when depolarized (right). The change in Rt_RC firing mode and hyperpolarization of VPL_TC cells resulted in a significant decrease of large amplitude IPSPs in VPL_TC cells and rebound bursts.
