# Supplementary material for: Thalamic control of sensory processing and spindles in a biophysical somatosensory thalamoreticular circuit model of wakefulness and sleep
Source: Cell Rep. 2023 Mar 1;42(3):112200. doi: 10.1016/j.celrep.2023.112200 (PMC10066598; doi:10.1016/j.celrep.2023.112200)
Supplement: Document S2. Article plus supplemental information [file mmc7.pdf]

Cell Reports

Resource

Thalamic control of sensory processing and spindles in a biophysical somatosensory thalamoreticular circuit model of wakefulness and sleep

Graphical abstract

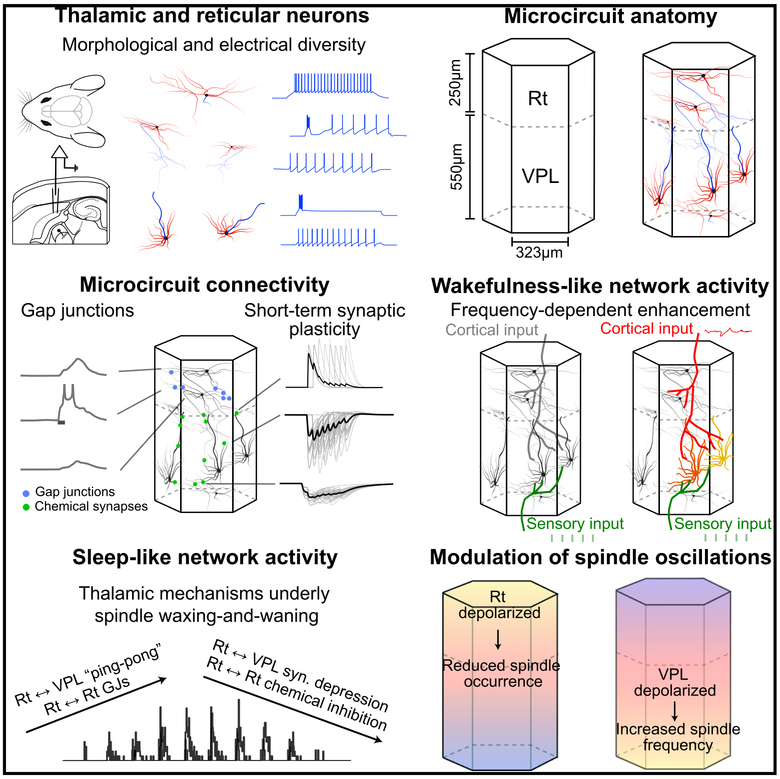

Highlights

- A computational model of thalamoreticular microcircuitry is presented and publicly available
- Thalamic inhibitory rebound leads to frequency-specific enhancement of sensory responses
- Interactions within the thalamus produce distinctive waxing and waning of spindle oscillations
- Alterations in thalamic excitability influence frequency and occurrence of spindle oscillations

Authors

Elisabetta Iavarone, Jane Simko, Ying Shi, ..., Francisco Clascá, Henry Markram, Sean L. Hill

Correspondence

elisabetta.iavarone01@ateneopv.it (E.I.), sean.hill@epfl.ch (S.L.H.)

In brief

Iavarone et al. present a computational model of thalamoreticular microcircuitry providing insight into thalamic interactions during wakefulness and sleep. They find common mechanisms that regulate the frequency-dependent enhancement of sensory responses and fluctuations of spindle oscillations, and discover that thalamic excitability controls spindle frequency and incidence.

Iavarone et al., 2023, Cell Reports *42*, 112200
March 28, 2023 © 2023 The Author(s).
https://doi.org/10.1016/j.celrep.2023.112200

CellPress

Cell Reports

CellPress
OPEN ACCESS

Resource

Thalamic control of sensory processing and spindles in a biophysical somatosensory thalamoreticular circuit model of wakefulness and sleep

Elisabetta Iavarone,[1,*] Jane Simko,[2] Ying Shi,[1,2] Marine Bertschy,[2] María García-Amado,[3] Polina Litvak,[1] Anna-Kristin Kaufmann,[1] Christian O'Reilly,[1] Oren Amsalem,[4] Marwan Abdellah,[1] Grigori Chevtchenko,[1] Benoît Coste,[1] Jean-Denis Courcol,[1] András Ecker,[1] Cyrille Favreau,[1] Adrien Christian Fleury,[1] Werner Van Geit,[1] Michael Gevaert,[1] Nadir Román Guerrero,[1] Joni Herttuainen,[1] Genrich Ivaska,[1] Samuel Kerrien,[1] James G. King,[1] Pramod Kumbhar,[1] Patrycja Lurie,[1] Ioannis Magkanaris,[1] Vignayanandam Ravindernath Muddapu,[1] Jayakrishnan Nair,[1] Fernando L. Pereira,[1] Rodrigo Perin,[2] Fabien Petitjean,[1] Rajnish Ranjan,[1] Michael Reimann,[1] Liviu Soltuzu,[1] Mohameth François Sy,[1] M. Anıl Tuncel,[1] Alexander Ulbrich,[1] Matthias Wolf,[1] Francisco Clascá,[3] Henry Markram,[1,2] and Sean L. Hill[1,5,6,7,8,*]

[1]Blue Brain Project, École polytechnique fédérale de Lausanne (EPFL), Campus Biotech, Geneva, Switzerland
[2]Laboratory of Neural Microcircuitry (LNMC), École polytechnique fédérale de Lausanne (EPFL), Lausanne, Switzerland
[3]Departamento de Anatomía, Histología y Neurociencia, Facultad de Medicina, Universidad Autónoma de Madrid, Madrid, Spain
[4]Department of Neurobiology, Hebrew University of Jerusalem, Jerusalem, Israel
[5]Department of Psychiatry, University of Toronto, Toronto, Canada
[6]Department of Physiology, University of Toronto, Toronto, Canada
[7]Krembil Centre for Neuroinformatics, Centre for Addiction and Mental Health (CAMH), Toronto, Canada
[8]Lead contact
*Correspondence: elisabetta.iavarone01@ateneopv.it (E.I.), sean.hill@epfl.ch (S.L.H.)
https://doi.org/10.1016/j.celrep.2023.112200

SUMMARY

Thalamoreticular circuitry plays a key role in arousal, attention, cognition, and sleep spindles, and is linked to several brain disorders. A detailed computational model of mouse somatosensory thalamus and thalamic reticular nucleus has been developed to capture the properties of over 14,000 neurons connected by 6 million synapses. The model recreates the biological connectivity of these neurons, and simulations of the model reproduce multiple experimental findings in different brain states. The model shows that inhibitory rebound produces frequency-selective enhancement of thalamic responses during wakefulness. We find that thalamic interactions are responsible for the characteristic waxing and waning of spindle oscillations. In addition, we find that changes in thalamic excitability control spindle frequency and their incidence. The model is made openly available to provide a new tool for studying the function and dysfunction of the thalamoreticular circuitry in various brain states.

INTRODUCTION

The thalamus and thalamic reticular nucleus (Rt) lie at the heart of the thalamocortical (TC) system in the mammalian brain.[2] Thalamic relay cells send projections to the cortex and form excitatory collaterals with thalamic reticular neurons. These neurons then send inhibitory projections back to the thalamus, creating the thalamoreticular circuit.[3–5] This circuit plays a key role in several brain functions, such as transmitting sensory information to the cortex and regulating brain states such as sleep and wakefulness. It has also been linked to attentional processes and is responsible for the generation of spindle oscillations during sleep.[6–10] Changes in thalamic neuron firing and connectivity have been associated with abnormal brain rhythms, such as those seen in absence epilepsy.[11–15] Alterations in the incidence and density of spindle oscillations during sleep have been observed in various disorders, including schizophrenia,[16–20] neurodevelopmental disorders,[21] attention-deficit/hyperactivity disorder (ADHD),[22] and Alzheimer's disease.[23]

In this work, we follow and extend the reconstruction pipeline presented in Markram et al.[24] to develop and validate a digital model of a thalamoreticular microcircuit of a portion of first-order somatosensory (ventral posterolateral nucleus [VPL]) thalamus in the adult mouse.

This study uses a computational model to examine the dynamics of the thalamoreticular circuit during wakefulness and sleep-like states. The model is able to replicate multiple experimental findings and provide new insights and predictions. In wakefulness, the model shows that the thalamoreticular circuitry can generate frequency-selective enhancement of thalamic responses. In sleep, the model reveals that the waxing and waning of spindle oscillations is generated intrathalamically, and that changes in cell excitability can alter spindle incidence and frequencies. We provide the experimental data and computational models as an open resource for further research.

This is an open access article under the CC BY license (http://creativecommons.org/licenses/by/4.0/).

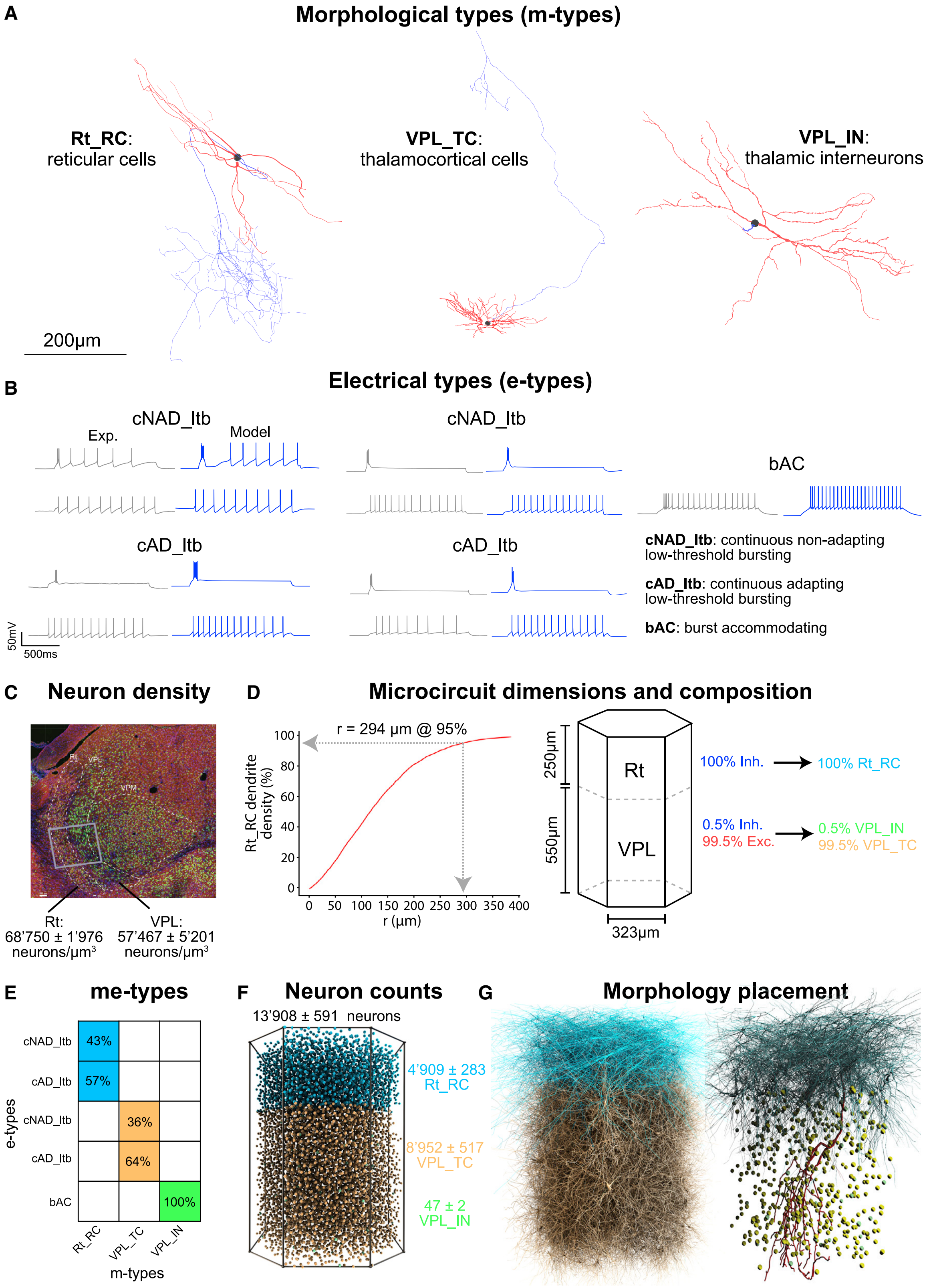

(legend on next page)

CellPress
OPEN ACCESS

RESULTS

Reconstructing morpho-electrical models of thalamic and reticular neurons

Morphological types

One-hundred and fifty-seven neuron morphologies were collected from *in vitro* and *in vivo* labeling experiments in mice, including data from the MouseLight Project at Janelia[25] and in-house experiments. The morphologies were classified into three m-types: VPL_TC, VPL_IN, and Rt_RC (Figure 1A). VPL_TC represented all TC neurons in the VPL, VPL_IN represented all thalamic interneurons (INs), and Rt_RC represented all neurons in the Rt. A validated morphological diversification pipeline was used to generate 92,970 unique morphologies that accounted for individual variability (see STAR Methods).

Electrical types

Over 100 TC neurons, INs, and reticular neurons were characterized through patch-clamp recordings (see STAR Methods) in mouse brain slices. TC neurons were classified as adapting (cAD_ltb) and non-adapting (cNAD_ltb) e-types (Figure 1B) based on their tonic firing responses, similar to those in rat TC cells.[26] Rt neurons also showed adapting and non-adapting responses. INs were classified as a single-burst-accommodating (bAC) e-type.[27]

Morpho-electrical models

A multi-objective optimization pipeline was used to build electrical models (e-models) for the e-types in Figure 1B, resulting in five e-models (see STAR Methods). These e-models were combined with 92,970 morphologies to generate 142,678 unique morpho-electrical models, which were assessed with a battery of tests to reject models with electrical features and firing behavior that differed significantly from the experiments.

Reconstructing thalamoreticular microcircuit structure

We defined the thalamoreticular microcircuit and determined its neuron numbers, composition, and positions (Figures 1C–1G). The microcircuit spans parts of the Rt and VPL of the thalamus. We chose the VPL nucleus because it receives information from the hindlimb[28] and is representative of a more generic non-barreloid thalamus.

The extent of the Rt_RC dendrites informed the horizontal dimension of the microcircuit (see STAR Methods), while the height of the circuit comes from the anatomical parcellation in the Allen Brain Atlas.[29] The resulting volume is ~0.22 mm^4 with a length of 323 μm and a height of 800 μm. Using semi-automated cell counting (see STAR Methods), we found an average cell density of 68,750 ± 1,976 cells/mm^3 in Rt and 57,467 ± 5,201 cells/mm^3 in VPL. 100% of Rt neurons are inhibitory, while 0.5% of VPL neurons are inhibitory (mean ± std, n = 37 slices).

The resulting model microcircuit contains a total of ~14,000 neurons (averaged over seven microcircuit instances) composed of 4,909 ± 283 Rt_RCs, 8,952 ± 517 VPL_TCs, and 47 ± 2 VPL_INs. The Rt has 100% m-type Rt_RC neurons with 57% cAD_ltb and 43% cNAD_ltb e-types. VPL has VPL_IN m-type with bAC e-type, and VPL_TC m-type with 64% cAD_ltb and 36% cNAD_ltb e-types. Soma positions were determined using a pseudorandom algorithm and taking into account morphological constraints.[4,28–32]

Reconstructing and validating synaptic connectivity

The connectivity between neurons in a microcircuit was established using an adapted algorithm based on Markram et al.[24] and Reimann et al.[33] A linear relation between the dendritic surface in the thalamus and bouton numbers on reticular axons[34] was found, suggesting predictability of functional synapse locations.[35] We used neuron morphologies and bouton densities as constraints. Our experimental dataset showed that TCs had 0.102 ± 0.021 boutons/μm (n = 9 axons) and Rt neurons 0.124 ± 0.002 boutons/μm (n = 2 axons). Synaptic connections were established by presynaptic axons and postsynaptic dendrites/somata (Figure 2). INs largely established connections through presynaptic dendrites, as observed in the visual thalamus.[36,37] Medial lemniscal (ML) and corticothalamic (CT) synapses were included using volumetric bouton densities as constraints (Figure 2D). Data on lemniscal synapses in the mouse VPM[38] was used and the relative proportions of CT to lemniscal synapses onto TCs and the ratio of CT to TCs synapses in the Rt.[39,40]

The model resulted in 4.77 million intrathalamic synapses and 17,998 lemniscal and 40,905 corticothalamic synapses. We compared the synapse convergence onto reticular neurons[41] and the distribution of number of synapses per connection,[36] and both gave results comparable with the experimental counterpart (Figures 2E and 2F).

A detailed map of synaptic physiology in a thalamic microcircuit

We modeled the physiology of synapses in the thalamic microcircuit using data from experiments and literature on short-term plasticity, postsynaptic potential amplitudes, time constant

Figure 1. Single-cell reconstructions, neuron densities, and microcircuit features

(A) 3D reconstructions of three different thalamic and reticular cell types (m-types) from a mouse. Axons are shown in blue, dendrites in red, and the cell bodies in black.

(B) Electrical types (e-types) and the models that match them. From left to right: examples of recordings (gray) and models (blue) that correspond to the Rt_RC, VPL_TC, and VPL_IN m-types. The two different firing modes of the cNAD_ltb and cAD_ltb e-types are shown: low-threshold bursting (first row) and tonic firing (second row).

(C) Average number of neurons in the Rt and VPL regions of the thalamus. A slice was stained with anti-GABA (red), anti-NeuN (green), and DAPI (blue), and the average number of neurons was calculated. The gray box shows a thalamic microcircuit.

(D) Dimensions of the microcircuit (lateral and vertical). The lateral size was determined by the smallest circle that captured the Rt_RC dendritic density in the center of the microcircuit. The vertical size was calculated from the Allen Reference Atlas. The excitatory/inhibitory ratios and m-type compositions are also shown.

(E) The fraction of e-types for each m-type found in our single-cell recordings.

(F) Predicted number of neurons and their positions in the microcircuit (mean and standard deviation of five microcircuits).

(G) The placement of cell morphologies in the microcircuit. Only 10% of the neurons are shown (left) and axons are not shown for clarity. The right image shows one example of an Rt_RC axon (red) innervating the VPL.

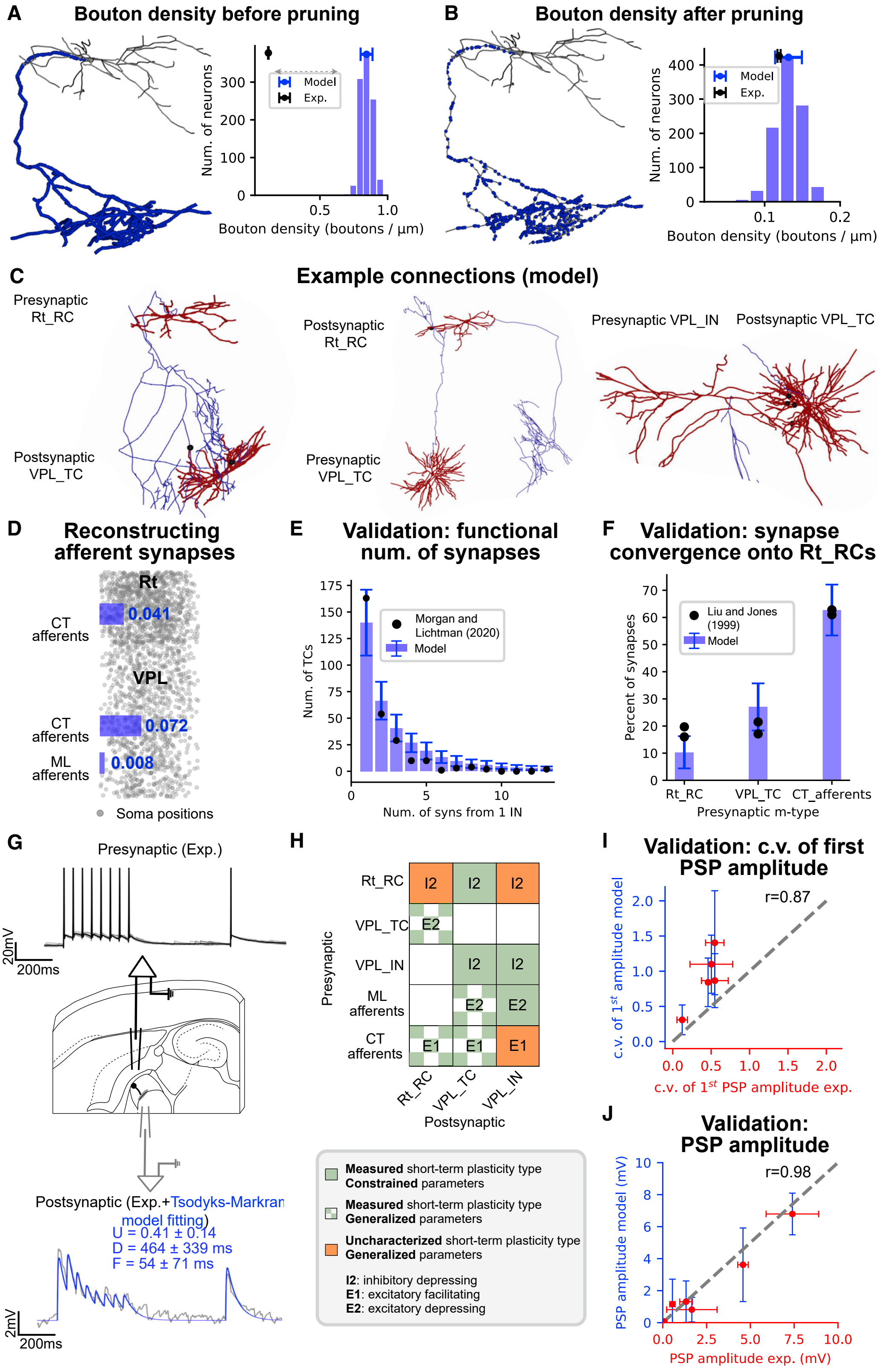

(legend on next page)

of synaptic currents, and reversal potentials. We identified three types of short-term plasticity—inhibitory depressing (I2), excitatory depressing (E2), and excitatory facilitating (E1)—and used synapse models featuring stochastic transmission and short-term plasticity.[24,42] We constrained the parameters of the Tsodyks-Markram model with available thalamic data (See STAR Methods; Table S1; Figures 2G and 2H). The model provides a comprehensive map of synapse types with the main external afferents (Figure 2H). The model was able to reproduce the stochastic nature of synaptic release and *in silico* paired-recording data, closely matching the experimental results (Tables S2 and S3; Figures 2I–L).

Gap junction connectivity between reticular neurons is predicted by dendrodendritic appositions

Rt neurons are connected through electrical synapses, contributing to synchronization in thalamic networks.[43–46] Gap junction (GJ)-coupled neurons are estimated to be 30%–50% of the total population in Rt[47] and play a role in synchronization and desynchronization balance in thalamic networks.[11]

In the model, we defined GJ locations by identifying dendrite-soma appositions and found that assigning GJs to 30% of appositions matched experimental results (Figure 3A). The model predicts that each neuron is directly coupled with 2–20 other neurons at distances of 40–120 μm, with rare coupling at 300–400 μm (Figure 3B), in line with experiment findings. Coupling strength was validated with a GJ conductance of 0.2 nS,[48] and calculated coupling coefficients between simulated paired recordings of 0.023 ± 0.008 (mean $\pm$ standard deviation, Figure 3C) were within the variability of mouse Rt neurons.[44] These results indicate that many aspects of GJ connectivity, in particular its distance dependence, can be predicted by the morphological properties of Rt_RC dendrites.

Spontaneous and evoked activity during wakefulness and sleep in the model thalamoreticular microcircuit

Spontaneous and evoked sensory activity (wakefulness-like)

We first explored spontaneous and evoked activity in the resulting microcircuit in a simulated *in vivo* wakefulness-like condition (Figure 4). In this condition, the model reproduces the distribution of firing rates in first-order thalamic and Rt neurons during quiet wakefulness, characterized by low firing rates (<10 Hz).[49–51] The simulation exhibits irregular firing activity in all m-types, with higher firing rates in VPL_INs due to their lower spiking threshold in the model (Figure 4B). Single-cell activities are dominated by single tonic spikes (Figure 4B), consistent with their predominance over low-threshold bursts in wakefulness-like states.[52]

We then compared spontaneous activity with sensory-evoked responses with brief activation of a subset of ML fibers, simulating a whisker flick or a brief stimulation of the hindlimb.[53] We found increased population firing rates in VPL_TC neurons, with a peak in firing in a short time window (5–10 ms) following the stimulus (Figures 4A and 4B; Video S1). This activity is followed by a period of silence in some cells, lasting 100–200 ms (Figure 4B). Rt_RCs exhibit an increase in firing rate compared with their spontaneous activity as a result of the excitation they receive from VPL_TCs (Figure 4B). The increased activity in Rt_RCs lasts for 50–100 ms after the stimulus and gradually decreases to baseline levels, resulting in a clear hyperpolarization of VPL_TCs (Figure 4A). The longer activation of Rt_RCs is in line with experimental findings and suggests an important role of the Rt in limiting sensory responses and focusing them on rapid perturbations.[50]

Simulating the activation of an increasing number of afferent ML fibers revealed increased recruitment of reticular inhibition (Figure S2A) and decreased response latency and variability with increasing stimulus size, increasing synchronous responses (Figure S2B). As expected, increased recruitment of inhibitory cells results in increased hyperpolarization of the VPL (Figures S2A and S2C).

At the topographical level (Figure S2D; Video S2), VPL cells located at the center responded to the stimulus, while the activation in the Rt was broader, with a degree of VPL-mediated depolarization in the periphery of the Rt. This result suggests that receptive fields in the Rt are larger than in the VPL, yet they provide topographically aligned inhibition to the thalamus in a focal manner, as shown for visual reticular neurons.[49,54] This result demonstrates that Rt can contribute to precise responses in the VPL not only by rapidly inhibiting directly responding neurons but also by limiting the response in the surrounding area.

Figure 2. Reconstructing and validating intrathalamic and thalamic afferent connectivity, short-term synaptic plasticity, and PSP amplitudes
(A and B) Neuron morphologies and bouton densities are used to constrain intrathalamic connectivity. (A) Putative synapses are identified using axodendritic appositions. High bouton densities (number of boutons per axonal length) characterize the resulting connectivity. Exemplar Rt_RC neuron (red, dendrites; blue, axon; black, soma) with putative synapse locations is shown on the left, and bouton density distribution for 1,000 Rt_RC morphologies in the model and experiment (n = 2 Rt_RC morphologies) on the right.
(B) Bouton densities from experiments are used to remove a fraction of axodendritic appositions.
(C) Resulting mono- and multi-synapse connections between neuron pairs are shown with black dots representing functional synapses.
(D) Volumetric bouton densities (boutons/μm3) were used to add synapses from medial lemniscal (ML) and corticothalamic (CT) afferents.
(E) Comparison of synapses per connection in the model and from an electron microscopy (EM) reconstruction of one IN in the mouse (Morgan and Lichtman[36]; N = 47 VPL_IN in the model).
(F) Validation of synapse convergence onto Rt_RC neurons in the model with EM experiments in the rat (N = 2, Liu and Jones,[41] N = 4,909 Rt_RCs in the model).
(G) *In vitro* paired recordings (eight pulses at 40 Hz followed by recovery stimulus) constrain the parameters of the Tsodyks-Markram model of short-term plasticity.
(H) Map of short-term plasticity types in the model (green, in-house experimentally characterized pathways; green checked, literature-derived pathways; orange, uncharacterized pathways).
(I) Validation of the coefficient of variation (c.v.) of first PSP amplitudes for five *in vitro*-characterized pathways (see Table S2).
(J) Comparison of PSP amplitudes in the model for seven characterized pathways in house or in the literature (see Table S3).

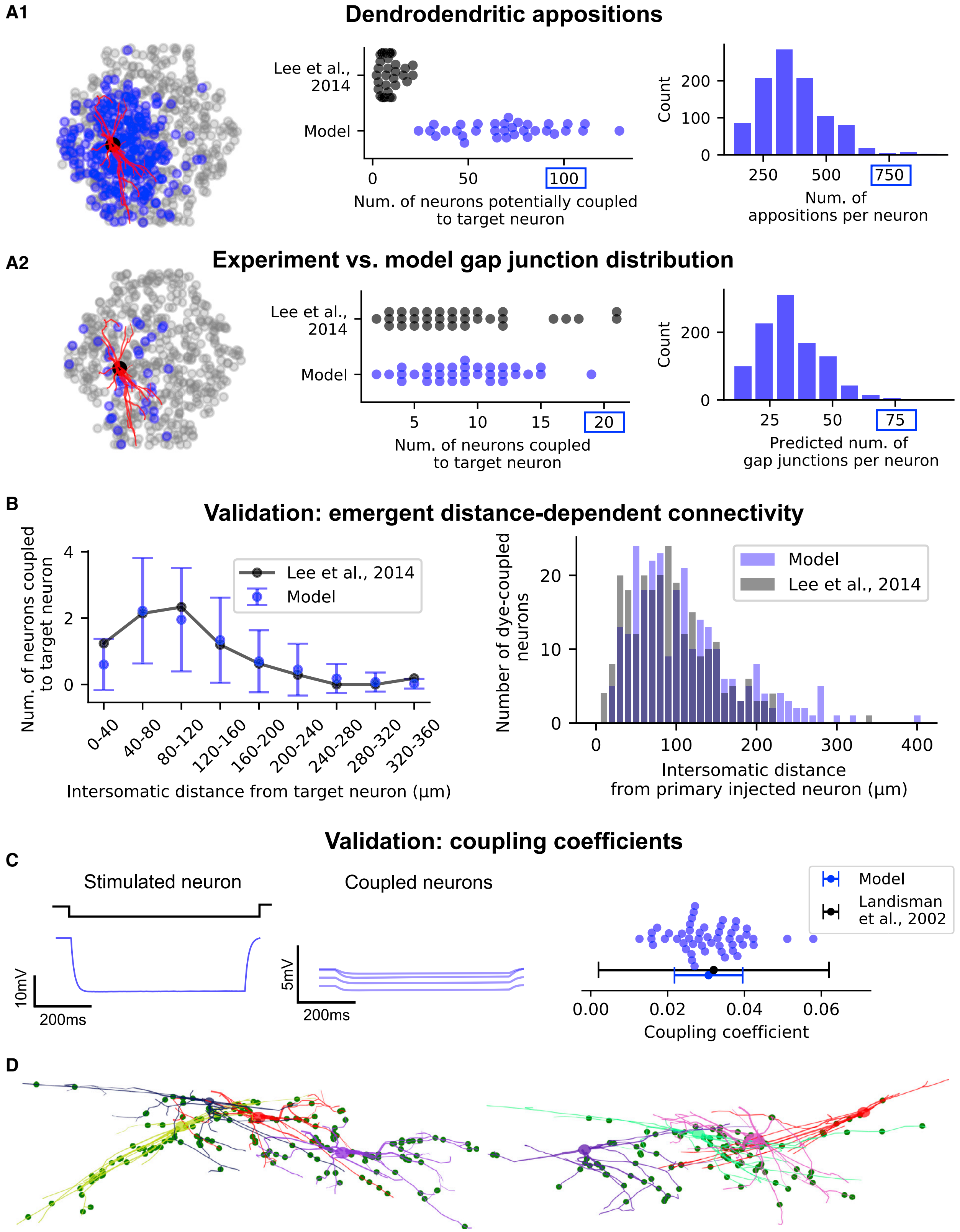

(legend on next page)

Simulated sleep-like cortical up and down states initiate spindle-like oscillations

Spindles often occur during cortical up states, when cortical neurons are more active.[55–57] To examine the model response in such conditions, we simulated a transition from a wakefulness-like to nonrapid eye movement (NREM)-like state. We simulated the up and down states that characterize NREM by periodically removing the firing background from the cortical afferents for 500 ms and reactivating it for another 500 ms to the same level as the one used for our standard *in vivo* wakefulness-like condition (Figures 4C and 4D; Video S3).

During the up state, CT input drives Rt activity and initiates spindle-like oscillations through post-inhibitory rebound in the VPL (Figure 4C). Interestingly, during the down state, Rt_RCs become highly hyperpolarized (<-70 mV), while VPL_TCs are less affected (Figure 4C). During the simulated cortical up state, Rt_RCs fire robustly, predominantly low-threshold bursts, causing deep inhibitory postsynaptic potentials (IPSPs) in the VPL_TCs that, in turn, often respond with post-inhibitory rebound bursts, the hallmark of spindle-like activity in the thalamus (Figure 4D).

Thalamic responses to sensory input exhibit adaptation and cortical enhancement

Numerous studies have shown adaptation to sensory stimuli in the lemniscal pathway due to short-term depression of lemniscal excitatory postsynaptic potentials (EPSPs).[58–62] To explore this, we set out to use the model to recreate a recent study that showed that depressed responses in the somatosensory thalamus can be enhanced by cortical activation in anesthetized mice.[63]

Consistent with experimental findings, activating lemniscal fibers, in the model, with trains of stimuli at 8 Hz results in high response probability to the first stimuli, while subsequent EPSPs exhibit decreased amplitudes (Figure 5A). The activation of cortical afferents 200 ms before the sensory stimulus with noisy input at a mean firing rate of 4 Hz increased the firing probability to all the other stimuli in the train, thus counterbalancing the adaptation (Figure 5B), as seen in the original study.[63]

Cortical enhancement of sensory responses is frequency dependent and frequency selective

The effects of the cortex on thalamic activity depend on the frequency and pattern of cortical activation.[64,65] In this study, when sensory stimuli of elevated frequencies (e.g., 10–20 Hz) were presented, thalamic responses were highly depressed and the probability of firing was greatly reduced (~0.1) (Figures 5C and 5D). When the mean firing rate of the cortex was increased, the efficacy of corticothalamic (CT) inputs in counterbalancing sensory adaptation also increased, as indicated by an increase in the firing probability of thalamic cells (Figure 5D). The cortical enhancement of sensory responses was found to be frequency selective, with the greatest enhancement observed when sensory stimuli were at a frequency of around 10 Hz. This was due to the recruitment of Rt inhibition and the activation of intrinsic subthreshold currents in thalamic cells (Figures 5E, S3F, and S3G). At higher frequencies of sensory stimuli, the effects of the cortex on thalamic activity were less pronounced due to synaptic short-term depression and limited activation of the low-threshold calcium current in thalamic cells (Figure 5E). The selective enhancement of 10-Hz sensory stimuli was most prominent when the mean CT firing rate was ≥10 Hz.

Spindle-like oscillations emerge through thalamoreticular interactions

The model exhibited spindle-like activity during simulated cortical up states. It is known that sleep spindles are generated through interactions between cells in the Rt and thalamic cells.[10,66] Numerous studies have explored the mechanisms underlying the generation of spindle-like oscillations.[6,10,67–71] This process involves the activation of Rt neurons, which hyperpolarize TC cells via inhibitory connections between the two cell types.[66,72] This hyperpolarization primes TC cells for rebound bursts, which perpetuate the spindle cycle through a "ping-pong" interaction between Rt and TC cells.[73] The model was not specifically designed to replicate these known mechanisms, but it was found to do so.

Activating the Rt increases thalamic bursting and initiates spindle-like oscillations

A subset of cells in the Rt were briefly activated with a current pulse (20 ms) to simulate optogenetic activation[74] (Figure S4). This caused brief (~250 ms) oscillatory responses in both Rt and VPL cells (Figure S4A). The local field potential in the VPL showed oscillations at ~10 Hz (Figure S4B), which is consistent with the spindle frequency range *in vivo*. Single-cell responses

Figure 3. Dendrodendritic overlap predicts Rt GJ connectivity

(A1) Potential connectivity based on dendrodendritic and somatic appositions between Rt_RC dendrites. The left panel shows the microcircuit from the Rt side, including a sample of 500 Rt_RC neurons (gray dots), a target Rt_RC morphology (2D projection, dendrites in red), and the location of Rt_RC neurons connected to the target (blue dots). The middle panel shows neuron divergence (number of postsynaptic neurons) in the model and literature[45] (N = 33 for both experiment and model), with each dot representing one target neuron. The right panel displays the distribution of potential connectivity divergence (number of appositions per neuron, for a sample of 1,000 Rt_RC neurons in the model).

(A2) Predicted GJs after removing dendrodendritic appositions to match average GJ divergence. The left panel is similar to A1. The middle panel shows that the neuron divergence in the model matches experimental findings, while the right panel shows that the resulting GJ divergence is reduced by an order of magnitude. Note the different maximal values in A1 and A2.

(B) Validation of distance-dependent GJs connectivity. The right panel shows results from *in silico* dye injections that reproduce dye-coupling experiments (n = 500 neurons in the model, n = 33 in the experiment), including mean and standard deviation.

(C) Validation of GJs functional properties. The left panel shows an example of *in silico* paired recordings, where an Rt_RC is stimulated with a hyperpolarizing current step, and its somatic potential, along with the somatic potential of all coupled neurons, is recorded. The ratio of the voltage response between a coupled neuron and the stimulated neuron is the coupling coefficient (CC). The right panel compares CC values in the model (n = 50 pairs, each one represented by a dot) with paired recordings from the literature, including mean and standard deviation.

(D) Resulting GJ connectivity, including an example of clusters of four Rt_RC neurons coupled by GJs and GJ locations. Each neuron morphology is represented by a different color, with axons omitted for clarity. Green dots show the detailed morphological location of GJs received by each of the neurons from the other three and from other Rt_RC neurons not shown.

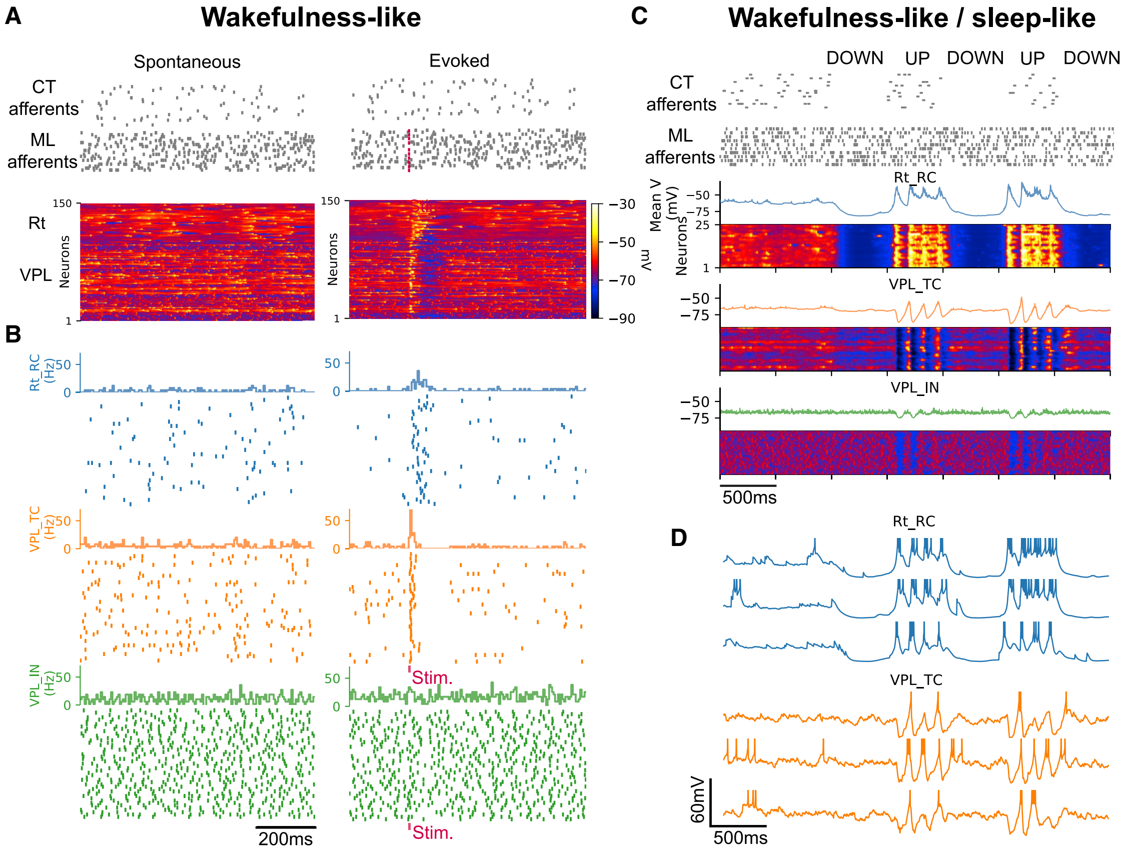

Figure 4. Wakefulness and sleep-like activity in the simulated thalamoreticular microcircuit

(A) Population voltage raster displays the membrane potential of a sample of 50 active neurons per m-type (group of neurons) in response to brief activation of 160 ML fibers. The activity is sorted by microcircuit depth and shows increased responses in both Rt and VPL and visible hyperpolarization in the VPL after the stimulus.

(B) Spike rasters and firing rate histograms of uncorrelated spiking activity in all m-types. VPL_IN neurons have higher firing rates. Rt_RCs show increased activity for a longer time after the stimulus compared with VPL neurons.

(C) The network is simulated in wakefulness-like conditions for the first 1,000 ms. Then, background activity from CT afferents is removed for 500 ms to approximate a cortical down state, followed by a 500-ms re-activation to simulate an up state. A sample of 25 neurons per each m-type is shown and color coded according to its membrane potential. The down state results in marked hyperpolarization in the Rt while spindle-like oscillations emerge during the up state.

(D) Sample of single-cell recordings from the neurons shown in (C). There is a change in firing mode during the NREM-like phase, where Rt_RC and VPL_TC fire mainly low-threshold bursts. Spikes are truncated at −25 mV.

also showed increased bursting in both Rt and VPL cells (Figure S4D). This synchronized burst firing in Rt cells was found to be a potent trigger of spindle oscillations (Figures S4D–S4G). These results confirm the important role of intrinsic neuronal mechanisms in the generation of spindle-like oscillations[6,75–79] and validate that optogenetic-like activation of the Rt in the model can trigger them, as shown by Halassa et al.[74]

Spindle-like oscillations are maintained by ping-pong interactions between the Rt and thalamus

To investigate the mechanisms of spindle-like oscillations, the network of thalamoreticular cells was simulated in a silent, hyperpolarized state (only receiving spontaneous synaptic release). When a group of neighboring Rt_RCs was activated (20-ms pulse; see Video S4), the duration of the resulting ping-pong interactions between VPL_TCs and Rt_RCs that mediate the spindle oscillation was influenced by the synaptic release probability (P_{rel}) (Figure 6A). Short-term synaptic depression, controlled by the recovery time constant (τ_{RecDep}), also affected the duration of the oscillations (Figure 6B). The frequency of the oscillations (~5–6 Hz; see Figure 6C) was similar to the barrages of IPSPs recorded in spindle waves in ferrets *in vitro*.[6] The study found that synaptic mechanisms, including P_{rel} and short-term synaptic depression, play a role in the duration of spindle-like oscillations, which was not previously known.

GJs increase the duration of spindle-like oscillations by propagating low-threshold bursts across the reticular network

GJs between reticular neurons contribute to the duration of spindle-like oscillations by synchronizing activity and transmitting low-threshold bursts, as shown in previous studies.[8,11,44,46] Our results show direct evidence of GJs' effect, as removal of GJs prevented spindle-like oscillations from occurring. Comparison of spiking activity and spatiotemporal patterns in the Rt and VPL revealed increased spiking and longer-lasting excitation in

CellPress
OPEN ACCESS

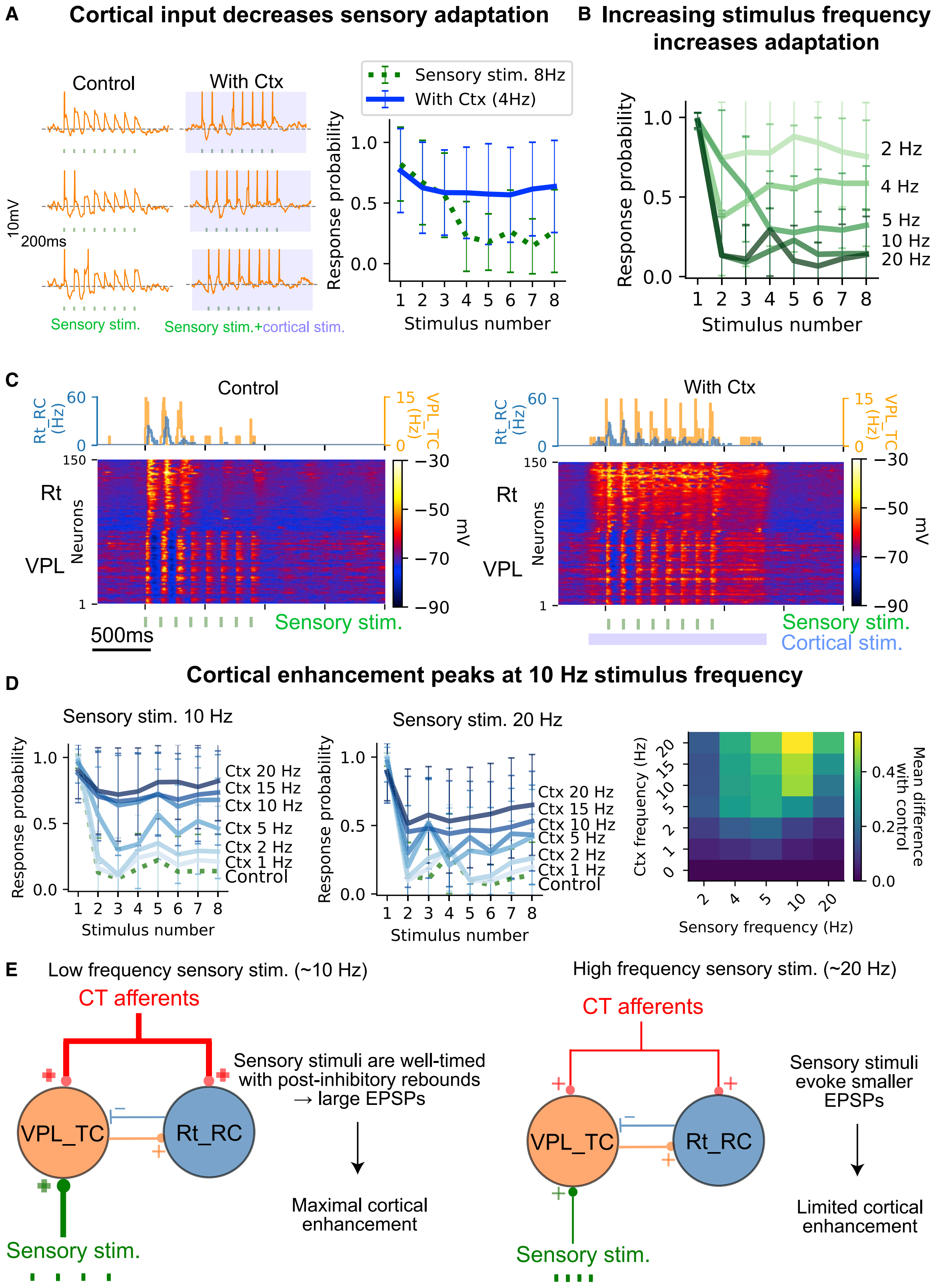

(legend on next page)

the Rt and higher inhibition in the VPL with the presence of GJs (Figures 6D–6I). GJs transmitted slow signals efficiently, with decreased amplitude in peripheral cells (Figure 6H; see Video S5 for comparison of spatiotemporal activity).

The termination of spindle-like oscillations is determined by short-term depression and buildup of intra-reticular inhibition

Several intrathalamic mechanisms have been identified as contributing to the termination of spindle activity. These include the Ca^{2+}-dependent upregulation of I_h current in thalamic cells,[68,80] hyperpolarization of reticular neurons, with the activation of Na^+- and Ca^{++}-dependent K^+ currents.[81] External inputs, such as desynchronized cortical activity and noradrenergic input from the locus coeruleus, have also been hypothesized to play a role in spindle termination.[82,83]

The model revealed additional cellular and synaptic mechanisms responsible for spindle-like oscillation termination, not previously investigated. Intrathalamic connections are governed by depressing synapses (Figure 2), which leads to decreased efficacy between Rt and VPL neurons. By scaling the time constant of recovery from short-term synaptic depression, it was found that reducing it to 85% of the control value in Rt_RC to VPL_TC connections caused the oscillation to not terminate (Figure 6B). Short-term depression in the VPL_TC to Rt_TC pathway had limited effect on termination and could even be removed if present in Rt_RCs to VPL_TCs. Rt_RC bursting activity is important for spindle-like oscillation initiation and duration, but these cells inhibit each other through GABAergic synapses. Inhibitory Rt-Rt synapses were studied and found to be crucial, as reducing their conductance led to non-terminating oscillations (Figure 6C) with an increase in Rt_RC cells participating in each cycle.

Waxing and waning of spindle-like oscillations emerges due to intrinsic cellular and synaptic mechanisms

Waxing and waning are defining characteristics of spontaneous sleep spindles recorded at the cortical level in the electroencephalogram (EEG), local field potential, and in thalamic recordings.[6] Despite this characteristic pattern of activity, the mechanisms underlying the spindle-shaped oscillation have not been clear.[84] It has been thought that TC and corticothalamic interactions were responsible for waxing and waning.[83,85–87]

In the model, we found that the waxing and waning of spindles can be generated within the thalamoreticular microcircuit alone (Figure 6L). Specifically, the waxing of a spindle oscillation is created by the rhythmic recruitment of neurons, first in the Rt, and second through the ping-pong interaction with the thalamus: each ping from the Rt successively recruits additional neurons and generate a stronger pong, via low-threshold bursts in VPL_TC cells. GJs augment this process through their ability to rapidly recruit Rt neurons. The waning of the spindle-like oscillation is a result of the progressive reduction in the probability of synaptic release (due to short-term synaptic depression), the subsequent decrease in postsynaptic potential (PSP) amplitudes, and the consequential reduction in recruitment of neuron firing during the ping-pong interaction between Rt and VPL neurons. At the same time, mutual inhibition between Rt_RCs progressively builds up, as additional Rt_RCs are recruited, and prevents the spread of the activity, acting as a self-limiting mechanism.

Depolarizing the Rt decreases spindle-like oscillation duration

Spindle oscillations in naturally sleeping (i.e., non-anesthetized) rodents are more easily evoked when thalamic activity is mildly synchronized and thalamic neurons are less active.[74,88] Their features, such as frequency and duration, evolve during NREM episodes.[88,89] In the model, we show that spindle duration can vary as a result of the membrane potential in the thalamus and the Rt.

We studied how membrane potential levels in Rt and VPL affect spindle-like oscillation duration, frequency, and peak firing (Figure 7). This was done by injecting noisy current into Rt_RCs and VPL_TCs to approximate the influence of neuromodulators on thalamic and reticular activity. A range of depolarization levels was explored, from hyperpolarized to near firing threshold.

Stimulating a subset of Rt_RCs with a 20-ms current pulse resulted in different spiking responses based on the depolarization level. When Rt_RCs were depolarized, spindle-like oscillation duration decreased (Figure 7A) and the number of spikes per burst decreased (Figure 7B), leading to reduced inhibition to VPL and decreased VPL_TC firing. As small as a 2- to 3-mV increase in membrane potential was sufficient to observe this effect. The waxing and waning in firing responses also tended to become predominantly waning when the Rt was depolarized (Figure 7A).

Depolarizing the thalamus increases spindle-like oscillation duration and frequency

When the membrane potential in the Rt is held constant (around -76 mV) and the VPL is depolarized, the oscillation increases in

Figure 5. Frequency-dependent sensory adaptation and cortical modulation of sensory responses in wakefulness

(A) Left: example of a VPL_TC cell response to a train of eight sensory stimuli delivered at 8 Hz (green). The cell only responded to the first stimulus in the train, demonstrating adaptation. Right: comparison of the firing probability of VPL_TC cells in response to the sensory stimulus alone (red) and with cortical activation (blue). The blue line represents an increase in firing probability with cortical activation. The markers indicate the mean probability in response to each stimulus, with the vertical line showing the standard deviation.

(B) The adaptation in the VPL to sensory responses increases with increasing frequency of the sensory stimulus.

(C) Comparison of population voltage rasters in the control condition and with cortical activation. The Rt responds to the first two or three stimuli in the train in both conditions, with visible hyperpolarization in the VPL.

(D) Left: the effect of different mean firing rates of cortical input on response probabilities for sensory stimuli at 10 and 20 Hz. Right: a map showing the efficacy of cortical input in counterbalancing sensory adaptation for different sensory frequencies and cortical mean firing rates.

(E) A schematic explaining why cortical enhancement is greater for sensory stimuli at around 10 Hz compared with higher frequencies (around 20 Hz). Sensory stimuli around 10 Hz are timed with post-inhibitory rebounds and activation of low-threshold calcium and produce larger EPSPs that can reach firing threshold with cortical activation. For higher stimulus frequencies, EPSPs decrease in amplitude due to synaptic depression, and cortical inputs are no longer sufficient to reach the firing threshold and counterbalance the adaptation.

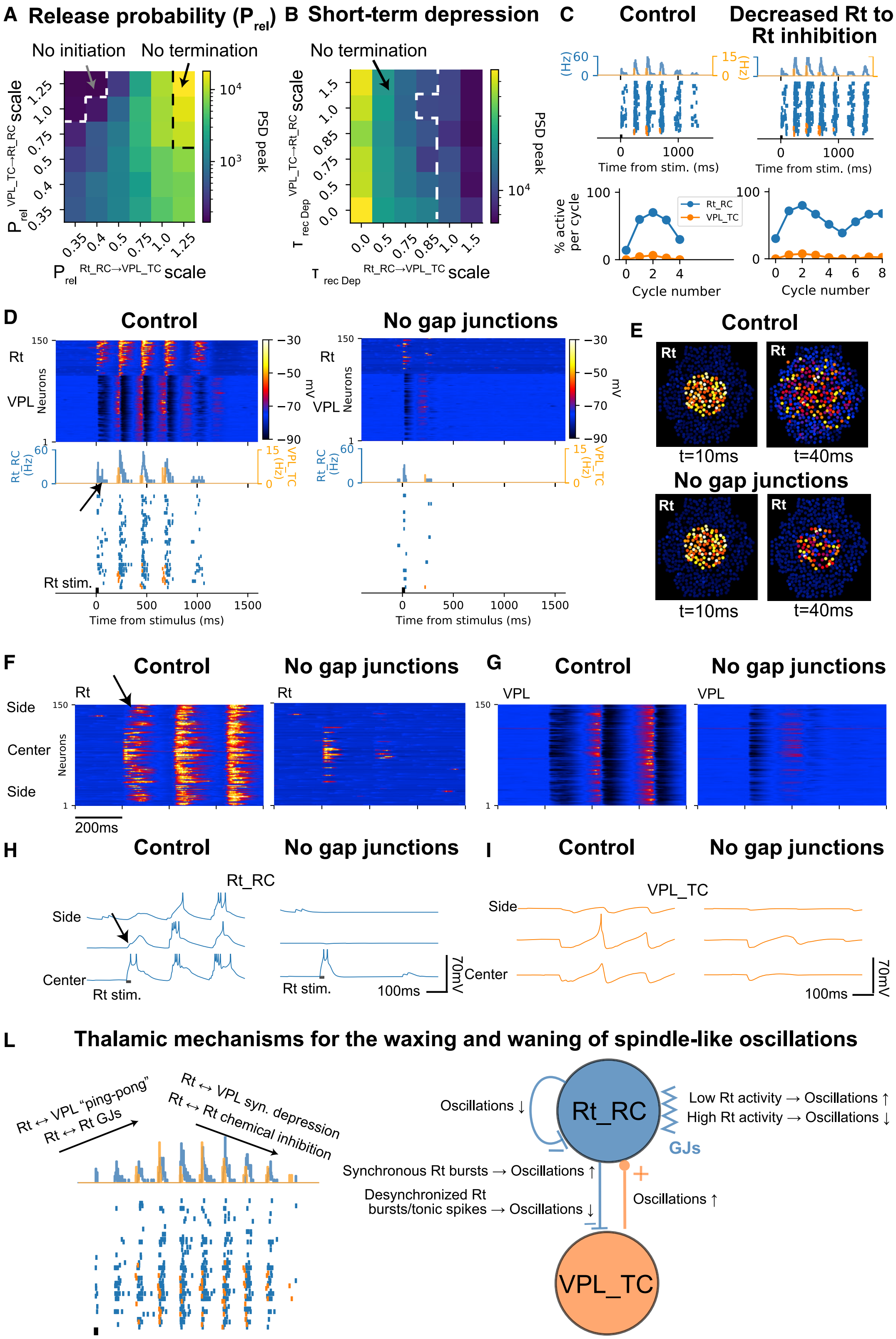

(legend on next page)

duration and frequency (Figure 7C). VPL depolarization results in deeper and faster IPSPs (Figure 7D). Deeper IPSPs result in stronger post-inhibitory rebound responses in VPL_TCs, which in turn excite more Rt_RCs causing a longer period of ping-pong interactions between the two populations, increasing the oscillation duration. Faster repolarization after the IPSPs is associated with post-inhibitory rebound responses occurring at shorter intervals, driving the ping-pong of activity at higher frequencies.

Differential depolarization of the Rt and thalamus modulates spindle properties

For each combination of membrane potentials in Rt_RCs and VPL_TCs, we calculated oscillation duration, frequency, and peak firing (Figures 7E–7G). The oscillation duration map shows that clear spindle-like oscillations (duration $\geq$ 500 ms) can only be evoked in a region where Rt_RC membrane potentials are below -75 mV and VPL_TCs are more depolarized than Rt_RCs. If we assume that VPL_TCs neurons are in general more depolarized during wakefulness than during sleep, and that Rt_RC neurons are more hyperpolarized, this result suggests that spindle-like oscillations are easier to evoke at the transition between wakefulness-like and sleep-like states, consistent with experimental observations.[8,90,91] When both VPL_TCs and Rt_RCs are at their baseline membrane potential, the frequency decreases to 5–6 Hz.

Simulating the effects of neuromodulation on thalamic and reticular neurons causes spindle-like oscillations to cease

Sleep spindles are a defining characteristic of stage 2 NREM sleep, and appear less frequently in deeper stages of NREM sleep.[8,90,91] The change in the incidence of spindles are due, at least in part, to neuromodulatory changes.[92–94] Our previous simulations, in the *in vitro*-like condition, showed that membrane potentials have a strong effect on oscillation duration and frequency (Figure S5). They also indicated that, when the VPL is hyperpolarized and the Rt is depolarized, spindles are less easy to evoke. We hypothesized that such differential polarization levels of the Rt and VPL would resemble the transition to deeper stages of NREM sleep and result in decreased incidence of spindles.

We tested this hypothesis with the model in the *in vivo*-like condition using simulated cortical up and down states (as in Figure 4). We then progressively depolarized the Rt and hyperpolarized the VPL, approximating the differential effect of neuromodulators on thalamic and reticular populations.[95–97] These simulated neuromodulatory changes cause the spindle-like oscillations to occur with reduced amplitude and then to cease, while the cortically-generated up-down states continue (Figure S5).

DISCUSSION

In this study, we developed a detailed model of the thalamoreticular microcircuit, a network of brain cells involved in sensory processing and rhythm generation. The model incorporates experimental data on the anatomy, physiology, and connectivity of individual neurons, as well as the three-dimensional (3D) organization of the reticular and VPL nuclei. It was validated across different simulated conditions, including wakefulness and sleep. The model showed that short-term synaptic plasticity and mutual inhibition within the Rt play key roles in the termination of spindle-like oscillations. It also found that GJs, corticothalamic feedback, and membrane potentials play a key role in modulating the duration and frequency of spindle-like oscillations. The model provides a comprehensive account of thalamoreticular network dynamics and can be used to interpret alterations in corticothalamic feedback and rhythm generation in health and disease. The model and accompanying data are openly available for future research (see section "data and code availability" below).

Comparison with prior work

The model differs from previous ones in several aspects, including scale (in terms of number of neurons), the level of biological detail, and scope.[68,69,83,98–102] While previous models were explicitly built and tuned to study network phenomena, such as sleep spindles, oscillations emerge in our model only by fitting cellular and synaptic properties, without tuning for network behavior. This data-driven approach enables novel insights on spindle generation and termination (see below). Prior models have largely used single-compartment neurons or simplified multicompartmental models,[103] while this model uses reconstructed 3D neuron morphologies to constrain the biophysical models and connectivity.

Morphological constraints on thalamoreticular connectivity

The model suggests that many properties of intrathalamic connectivity can be predicted by the axodendritic and dendrodendritic overlap of neuron morphologies. This includes the distribution of synapse locations and the number of synapses per connection

Figure 6. Spindle-like oscillations arise from intrinsic cellular and synaptic dynamics, with GJs enhancing the oscillations by recruiting low-threshold spikes in reticular cells

(A and B) In this figure, the circuit is in an *in vitro*-like environment, leading to hyperpolarized membrane potentials and stronger synaptic interactions. One-thousand Rt_RC cells are stimulated with a 20-ms current pulse. The parameter map in (A) shows the effect of synapse release probability between Rt_RC and VPL_TC cells on oscillation strength. In (B), the map shows the effect of short-term synaptic depression on the evoked oscillations.

(C) The inhibitory connections between Rt_RC cells play a role in termination.

(D) The spindle-like oscillation in control conditions (left) and with GJs removed (right).

(E) The topographical activity maps at 10 and 40 ms after the stimulus.

(F) The membrane potential along the lateral extent of the microcircuit.

(G) The same for VPL_TC cells.

(H) Single-cell recordings of Rt_RC.

(I) The same for VPL_TCs.

(L) Left: schematic of mechanisms underlying the waxing and waning of spindle-like oscillations. Right: connections can have positive/negative effects on oscillations.

CellPress
OPEN ACCESS

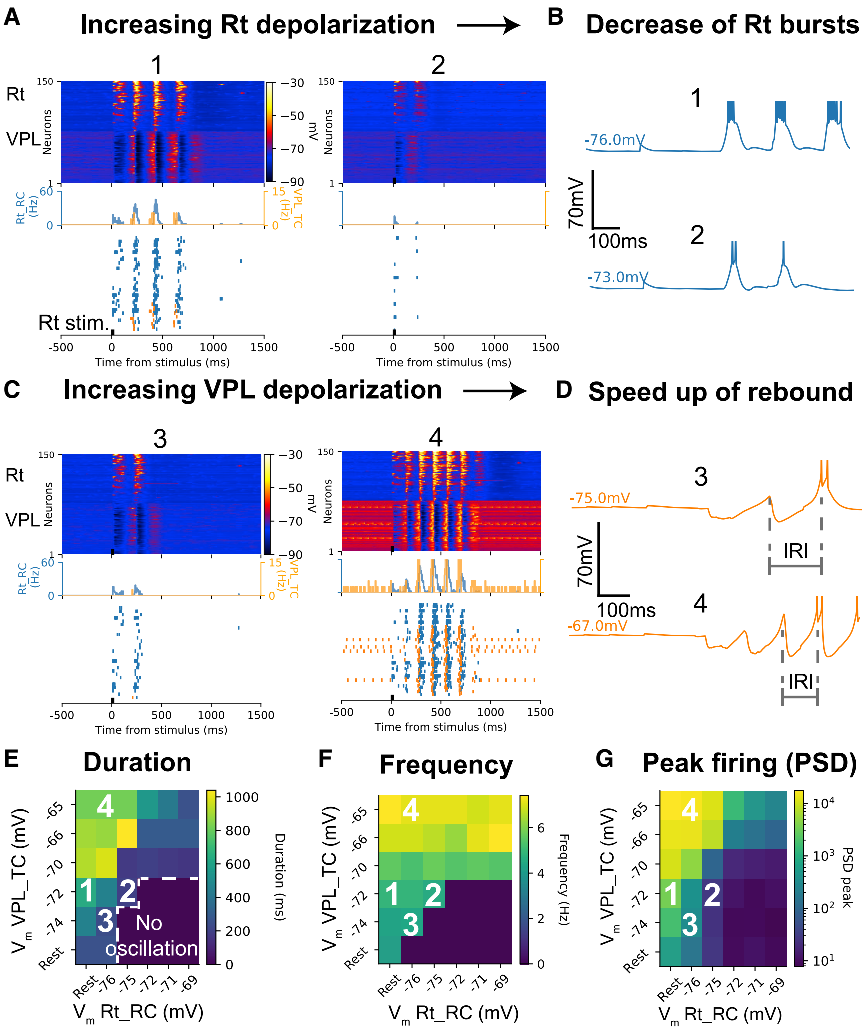

Figure 7. Depolarization levels affect spindle-like oscillation properties

(A) Voltage rasters and firing rate histograms of Rt_RCs showing decrease in oscillation duration with increased Rt depolarization.

(B) Single Rt_RC showing fewer spikes per burst with increased depolarization.

(C) Voltage rasters and firing rate histograms of VPL_TCs showing increase in oscillation duration and frequency with VPL depolarization.

(D) Single VPL_TC recording showing increased rebound responses and faster responses with VPL depolarization.

(E–G) Parameter maps showing effect of depolarizing VPL and Rt on oscillation (E) duration, (F) frequency, and (G) peak firing rate/power spectral density (PSD), respectively. VPL depolarization increases duration and frequency, while Rt depolarization decreases them.

established by thalamic INs, as well as the convergence of synapses from TC axon collaterals, other reticular neurons, and corticothalamic afferents onto reticular neurons. The model accurately recreates the distribution of single and multi-synaptic connections,[36] and it recapitulates distance-dependent GJ connectivity between reticular neurons, as observed in experimental studies.[45] The model also shows that dendrodendritic appositions provide a sufficient basis for determining GJ locations, and that the distance-dependent distribution of GJs between reticular neurons is determined by the extent of their dendrodendritic overlap.

Common cellular and synaptic mechanisms between sensory enhancement and spindle generation

The model recreates the network dynamics of spontaneous and sensory-evoked activity in wakefulness-like states. It allows for simultaneous observation of direct and indirect sensory responses in the thalamus and Rt. Responses to stimuli are clearly visible in the Rt, as has been shown experimentally with sensory, auditory, or visual stimuli.[50,53,104] The model also shows that robust inhibition from the Rt generates strong surround inhibition in the VPL, and that cortical activation sharpens the spatial properties of sensory inputs by evoking stronger Rt-mediated surround inhibitions. This is in line with recent research on the visual thalamus.[49]

As previously reported in anesthetized animals, the responses to trains of sensory stimuli were adapting for relatively low frequencies of 4–5 Hz, and the degree of adaptation increased with the increase of stimulus frequency.[59,60,62] The adaptation was reduced by simulated cortical activation, approximating cortical activity during activated states, allowing the transmission of high-frequency inputs.[63]

The model showed that the thalamoreticular microcircuit exhibits frequency-selective cortical enhancement. When simulating different frequencies of input and corticothalamic activation, peak enhancement of thalamic responses was observed for lemniscal inputs around 10 Hz. This enhancement occurs because cortical activation recruits sufficient Rt inhibition to activate I_h and low-threshold Ca^{2+} currents, enhancing the gain of input to TC cells at ~10 Hz. This is notable because rhythmic activity around this frequency emerges in TC networks during different behavioral states such as sleep spindles and alpha oscillations during attention.[105]

Our simulations suggest that ~10-Hz rhythms are intrinsic to thalamoreticular networks and could be responsible for enhanced gain of sensory inputs and TC activity around that frequency. These results are consistent with the observation of overlapping thalamic mechanisms between sensory processing, attention, and sleep.[105]

A novel cellular and synaptic account of spindle generation

The model provides novel insights into the mechanisms underlying the generation of sleep spindles in thalamic networks, including the role of TC-Rt synapse efficacy, short-term synaptic

depression, mutual inhibition between Rt neurons and GJs in the thalamic generation of waxing-and-waning spindle-like oscillations.

The model generates spindle-like oscillations without being explicitly designed for this purpose. The model is based on *in vitro* findings, but many aspects closely resemble thalamic activities during spindle oscillations in rodents *in vivo*.[88,89,106] This indicates that the model is robust and can generate spindle-like oscillations within experimentally plausible parameter ranges.

Consistent with previous experimental and modeling studies, spindle oscillations in the model are generated through a combination of intrinsic mechanisms, namely low-threshold calcium bursting in reticular neurons[75,77] and the synaptic interactions between reticular and TC neurons.[67,68,107]

Dendrodendritic GJs between reticular neurons have been known to transmit low-threshold bursts between cells, promote spiking correlations and synchronized activity.[44,46] The model shows that GJs also influence spindle duration. Furthermore, the spatial organization of Rt dendrites and their connections through GJs also shows a clear functional role in propagating the stimuli along the horizontal dimension of the microcircuit and enhancing spindle-like oscillations.

The model showed that spindle termination can occur through synaptic mechanisms alone, without requiring specific ionic mechanisms such as Ca^{2+}-dependent I_h current upregulation in TC cells or cortical input desynchronization.[68,83,100,102] The model revealed that mechanisms such as short-term synaptic depression and mutual inhibition between reticular neurons limit the duration of the oscillation. While mutual inhibition has been previously suggested as a factor,[11,13,14,70,108] short-term synaptic depression has not been considered as a termination mechanism. Based on the role of Rt-Rt mutual inhibition, which decreases spindle duration, and GJs, which tend to prolong it, we propose that Rt interactions have a self-limiting contribution to spindle oscillations. GJ coupling promotes synchronized IPSPs and TC excitation, recruiting more Rt neurons. When a critical recruitment level is reached, the overall excitation is countered by reciprocal inhibition and short-term synaptic depression, and the oscillatory activity limits itself.

Thalamoreticular circuitry is responsible for the waxing and waning of spindles

The gradual increase in amplitude (waxing) and the gradual decline (waning) of spindle oscillations has been hypothesized to be due to changes in cortical activity impinging on the Rt and thalamus.[83,85–87] However, the model demonstrates that cellular and synaptic mechanisms of thalamoreticular circuitry that underlie the increased recruitment of additional neurons during each cycle of a spindle can produce the waxing phenomena, while synaptic depression and the buildup of inhibition within the Rt are sufficient to explain the waning phenomena.

Relative differences in the excitability of thalamic and reticular nuclei can explain differences in spindle frequency, density, and amplitude

We found that changes in depolarization level of thalamic and reticular neurons affect oscillation frequency, duration, strength, and incidence of spindle-like oscillations. Our model approximates the dynamic change of thalamic network states during transitions between wakefulness and sleep, based on known mechanisms of action of neuromodulators such as acetylcholine (ACh) and noradrenaline (NA). ACh levels decrease during the transition from wakefulness to NREM sleep, causing hyperpolarization of the Rt and depolarization of the thalamus.[95–97] NA levels fluctuate during NREM sleep *in vivo*, leading to depolarized membrane potentials that affect low-threshold bursting activity underlying sleep spindles.[93] Other alterations in thalamic and reticular excitability could be due to changes in synaptic activity and plasticity of corticothalamic and corticoreticular projections.[109]

These findings have important implications when considering changes in sleep spindle frequency, density, and amplitude as potential biomarkers of disease. The model could be used to study the mechanisms that affect spindle oscillations and their properties during sleep in conditions such as schizophrenia,[16–19,21] ADHD,[22] and Alzheimer's disease.[23] For example, spindle densities have been found to decrease in patients with Parkinson's disease, and their density and duration are sensitive to Alzheimer's disease.[23,110–112] Previous research has suggested that the cortex is required for the generation of slow spindles,[113] but the present model shows that the thalamus can modulate the frequency of spindle-like oscillations, indicating that thalamic mechanisms may be sufficient to generate both types of spindles.

Limitations of the study

The model presented here is a first-draft reconstruction of thalamoreticular microcircuitry and is not intended to be a complete representation of the full circuitry. The model does not account for reciprocal connectivity with the cortex and therefore is not sufficient to explore the role of the thalamus in higher-order functions that depend on the cortex, such as attention and cognition. We are making the model openly available to facilitate future extensions and refinements.

This model represents a primary somatosensory microcircuit and includes only the "core" cell characteristic of sensory thalamic nuclei that target cortical layers III and IV, rather than the "matrix" cells, found in higher-order thalamic nuclei that target supragranular layers. Different thalamic nuclei have different mixtures of excitatory cells that have unique projection characteristics to the cortex.[114,115] Future refinements could take into account these different cell populations.

In addition, the cellular e-types in the present model do not capture the full diversity of known ion channels implicated in bursting behavior in reticular neurons or dendritic properties of thalamic INs. Inclusion of additional and more specific ion channel mechanisms, (e.g., variants of low-threshold Ca^{2+} Cav3.1, Cav3.2, and Cav3.3) in TC and Rt neurons could more accurately reproduce differences in bursting behavior in thalamic and reticular neurons, as well as dendritic properties of thalamic INs.[75,77,80,116,117]

Further refinements of the model, such as including the distribution of different morphological types within the thalamic and reticular domains, could improve its accuracy.[118–121] Additionally, incorporating the laminar structure[120,122,123] of the Rt and coupling the thalamoreticular model with cortical microcircuitry[24]

could provide a more comprehensive understanding of the role of TC interactions in sensory processing, attention, and rhythm generation across wakefulness and sleep.

STAR★METHODS

Detailed methods are provided in the online version of this paper and include the following:

- KEY RESOURCES TABLE
- RESOURCE AVAILABILITY
  - Lead contact
  - Materials availability
  - Data and code availability
- EXPERIMENTAL MODEL AND SUBJECT DETAILS
- METHOD DETAILS
  - Constraining and validating the model with experimental data
  - Experimental data used to constrain the model
  - Experimental data used for model validation
  - Validations at the network level
  - Reconstructing the morphological diversity of neurons
  - Morphology analysis, alignment and visualization
  - Unraveling morphologies
  - Repairing morphologies
  - Morphology diversification
  - Reconstructing the electrical diversity of neurons
  - Measuring neuron density
  - Reconstructing the dimensions and structure of a thalamoreticular microcircuit
  - Soma positions and me-type model assignment
  - Morphology placement
  - Generating different microcircuit instances
  - Reconstructing the synaptic connectivity of a thalamoreticular microcircuit
  - Modeling synapse physiology
  - Synapse models
  - Constraining synapse conductance values
  - Modeling gap junctions
  - Simulation methods and conditions

SUPPLEMENTAL INFORMATION

Supplemental information can be found online at https://doi.org/10.1016/j.celrep.2023.112200.

ACKNOWLEDGMENTS

This study was supported by funding to the Blue Brain Project, a research center of the École Polytechnique Fédérale de Lausanne (EPFL), from the Swiss government's ETH Board of the Swiss Federal Institutes of Technology. S.H. receives support from the Krembil Foundation. M.G.A. and F.C. were funded by the European Union's Horizon 2020 Research and Innovation Program, European Commission (grant agreement nos. HBP SGA2 785907 and SGA3 945539) and Ministerio de Ciencia e Innovación FLAG-ERA grant NeuronsReunited (MICINN-AEI PCI2019-111900-2).

We thank Karin Holm from the Blue Brain Project for her assistance with manuscript editing. We also thank our experimental collaborators Mario Rubio and César Porrero (Autonomous University of Madrid, Spain), and Yun Wang and the team at Wenzhou Medical University, for their contributions with morphology reconstructions.

AUTHOR CONTRIBUTIONS

Conceptualization, E.I. and S.L.H; methodology, E.I., C.O., O.A., A.E., and M.R.; software, B.C., J.C., W.V.G., M.G., J.H., G.I., J.G.K., P.K., I.M., F.L.P., M.R., A.T., and M.W.; validation, E.I., S.L.H., V.R.P., and P.L.; formal analysis, E.I.; investigation, E.I., J.S., Y.S., M.B., and M.G.A.; data curation, E.I., A.K., P.L., J.N., R.R., L.S., M.F.S., and A.U., writing – original draft, E.I. and S.L.H.; writing – review & editing, E.I., P.L., and S.L.H.; visualization, E.I., M.A., G.C., C.F., A.C.F., N.R.G., and F.P.; supervision, R.P., F.C., H.M., and S.L.H.; funding acquisition, H.M. and S.L.H.

DECLARATION OF INTERESTS

The authors declare no competing interests.

INCLUSION AND DIVERSITY

We support inclusive, diverse, and equitable conduct of research.

Received: April 15, 2022
Revised: January 4, 2023
Accepted: February 15, 2023
Published: March 1, 2023

REFERENCES

1. Dai, K., Hernando, J., Billeh, Y.N., Gratiy, S.L., Planas, J., Davison, A.P., Dura-Bernal, S., Gleeson, P., Devresse, A., Dichter, B.K., et al. (2020). The SONATA data format for efficient description of large-scale network models. PLoS Comput. Biol. *16*, e1007696. https://doi.org/10.1371/journal.pcbi.1007696.
2. Jones, E.G. (2007). The Thalamus, Second edition (Cambridge University Press). 1939-2011.
3. Ohara, P.T., and Lieberman, A.R. (1985). The thalamic reticular nucleus of the adult rat: experimental anatomical studies. J. Neurocytol. *14*, 365–411.
4. Pinault, D. (2004). The thalamic reticular nucleus: structure, function and concept. Brain Res. Brain Res. Rev. *46*, 1–31. https://doi.org/10.1016/j.brainresrev.2004.04.008.
5. Scheibel, M.E., and Scheibel, A.B. (1966). The organization of the nucleus reticularis thalami: a Golgi study. Brain Res. *1*, 43–62. https://doi.org/10.1016/0006-8993(66)90104-1.
6. Bal, T., von Krosigk, M., and McCormick, D.A. (1995). Synaptic and membrane mechanisms underlying synchronized oscillations in the ferret lateral geniculate nucleus in vitro. J. Physiol. *483*, 641–663. https://doi.org/10.1113/jphysiol.1995.sp020612.
7. Contreras, D., Destexhe, A., Sejnowski, T.J., and Steriade, M. (1997). Spatiotemporal patterns of spindle oscillations in cortex and thalamus. J. Neurosci. *17*, 1179–1196.
8. Fernandez, L.M.J., and Lüthi, A. (2020). Sleep spindles: mechanisms and functions. Physiol. Rev. *100*, 805–868. https://doi.org/10.1152/physrev.00042.2018.
9. Steriade, M., Domich, L., Oakson, G., and Deschênes, M. (1987). The deafferented reticular thalamic nucleus generates spindle rhythmicity. J. Neurophysiol. *57*, 260–273.
10. von Krosigk, M., Bal, T., and McCormick, D.A. (1993). Cellular mechanisms of a synchronized oscillation in the thalamus. Science *261*, 361–364.
11. Beenhakker, M.P., and Huguenard, J.R. (2009). Neurons that fire together also conspire together: is normal sleep circuitry hijacked to generate epilepsy? Neuron *62*, 612–632. https://doi.org/10.1016/j.neuron.2009.05.015.

12. Huguenard, J.R., and McCormick, D.A. (2007). Thalamic synchrony and dynamic regulation of global forebrain oscillations. Trends Neurosci. *30*, 350–356. https://doi.org/10.1016/j.tins.2007.05.007.

13. Makinson, C.D., Tanaka, B.S., Sorokin, J.M., Wong, J.C., Christian, C.A., Goldin, A.L., Escayg, A., and Huguenard, J.R. (2017). Regulation of thalamic and cortical network synchrony by Scn8a. Neuron *93*, 1165–1179.e6. https://doi.org/10.1016/j.neuron.2017.01.031.

14. Sohal, V.S., and Huguenard, J.R. (2003). Inhibitory interconnections control burst pattern and emergent network synchrony in reticular thalamus. J. Neurosci. *23*, 8978–8988.

15. Steriade, M. (2005). Sleep, epilepsy and thalamic reticular inhibitory neurons. Trends Neurosci. *28*, 317–324. https://doi.org/10.1016/j.tins.2005.03.007.

16. Castelnovo, A., Graziano, B., Ferrarelli, F., and D'Agostino, A. (2018). Sleep spindles and slow waves in schizophrenia and related disorders: main findings, challenges and future perspectives. Eur. J. Neurosci. *48*, 2738–2758. https://doi.org/10.1111/ejn.13815.

17. Ferrarelli, F., Huber, R., Peterson, M.J., Massimini, M., Murphy, M., Riedner, B.A., Watson, A., Bria, P., and Tononi, G. (2007). Reduced sleep spindle activity in schizophrenia patients. Am. J. Psychiatr. *164*, 483–492. https://doi.org/10.1176/ajp.2007.164.3.483.

18. Ferrarelli, F., Peterson, M.J., Sarasso, S., Riedner, B.A., Murphy, M.J., Benca, R.M., Bria, P., Kalin, N.H., and Tononi, G. (2010). Thalamic dysfunction in schizophrenia suggested by whole-night deficits in slow and fast spindles. Am. J. Psychiatr. *167*, 1339–1348. https://doi.org/10.1176/appi.ajp.2010.09121731.

19. Manoach, D.S., Demanuele, C., Wamsley, E.J., Vangel, M., Montrose, D.M., Miewald, J., Kupfer, D., Buysse, D., Stickgold, R., and Keshavan, M.S. (2014). Sleep spindle deficits in antipsychotic-naïve early course schizophrenia and in non-psychotic first-degree relatives. Front. Hum. Neurosci. *8*, 762. https://doi.org/10.3389/fnhum.2014.00762.

20. Manoach, D.S., Pan, J.Q., Purcell, S.M., and Stickgold, R. (2016). Reduced sleep spindles in schizophrenia: a treatable endophenotype that links risk genes to impaired cognition? Biol. Psychiatr. *80*, 599–608. https://doi.org/10.1016/j.biopsych.2015.10.003.

21. Gruber, R., and Wise, M.S. (2016). Sleep spindle characteristics in children with neurodevelopmental disorders and their relation to cognition. Neural Plast. *2016*, 4724792. https://doi.org/10.1155/2016/4724792.

22. Saito, Y., Kaga, Y., Nakagawa, E., Okubo, M., Kohashi, K., Omori, M., Fukuda, A., and Inagaki, M. (2019). Association of inattention with slow-spindle density in sleep EEG of children with attention deficit-hyperactivity disorder. Brain Dev. *41*, 751–759. https://doi.org/10.1016/j.braindev.2019.05.004.

23. Weng, Y.-Y., Lei, X., and Yu, J. (2020). Sleep spindle abnormalities related to Alzheimer's disease: a systematic mini-review. Sleep Med. *75*, 37–44. https://doi.org/10.1016/j.sleep.2020.07.044.

24. Markram, H., Muller, E., Ramaswamy, S., Reimann, M.W., Abdellah, M., Sanchez, C.A., Ailamaki, A., Alonso-Nanclares, L., Antille, N., Arsever, S., et al. (2015). Reconstruction and simulation of neocortical microcircuitry. Cell *163*, 456–492. https://doi.org/10.1016/j.cell.2015.09.029.

25. Winnubst, J., Bas, E., Ferreira, T.A., Wu, Z., Economo, M.N., Edson, P., Arthur, B.J., Bruns, C., Rokicki, K., Schauder, D., et al. (2019). Reconstruction of 1,000 projection neurons reveals new cell types and organization of long-range connectivity in the mouse brain. Cell *179*, 268–281.e13. https://doi.org/10.1016/j.cell.2019.07.042.

26. Iavarone, E., Yi, J., Shi, Y., Zandt, B.-J., O'Reilly, C., Van Geit, W., Rössert, C., Markram, H., and Hill, S.L. (2019). Experimentally-constrained biophysical models of tonic and burst firing modes in thalamocortical neurons. PLoS Comput. Biol. *15*, e1006753. https://doi.org/10.1371/journal.pcbi.1006753.

27. Simko, J., and Markram, H. (2021). Morphology, physiology and synaptic connectivity of local interneurons in the mouse somatosensory thalamus. J. Physiol. *599*, 5085–5101. https://doi.org/10.1113/JP281711.

28. Francis, J.T., Xu, S., and Chapin, J.K. (2008). Proprioceptive and cutaneous representations in the rat ventral posterolateral thalamus. J. Neurophysiol. *99*, 2291–2304. https://doi.org/10.1152/jn.01206.2007.

29. Goldowitz, D. (2010). Allen reference Atlas. A digital color brain Atlas of the C57BL/6J male mouse - by H. W. Dong. Gene Brain Behav. *9*, 128. https://doi.org/10.1111/j.1601-183X.2009.00552.x.

30. de Biasi, S., Frassoni, C., and Spreafico, R. (1986). GABA immunoreactivity in the thalamic reticular nucleus of the rat. A light and electron microscopical study. Brain Res. *399*, 143–147. https://doi.org/10.1016/0006-8993(86)90608-6.

31. Houser, C.R., Vaughn, J.E., Barber, R.P., and Roberts, E. (1980). GABA neurons are the major cell type of the nucleus reticularis thalami. Brain Res. *200*, 341–354.

32. Erö, C., Gewaltig, M.-O., Keller, D., and Markram, H. (2018). A cell Atlas for the mouse brain. Front. Neuroinf. *12*, 84. https://doi.org/10.3389/fninf.2018.00084.

33. Reimann, M.W., King, J.G., Muller, E.B., Ramaswamy, S., and Markram, H. (2015). An algorithm to predict the connectome of neural microcircuits. Front. Comput. Neurosci. *9*, 120. https://doi.org/10.3389/fncom.2015.00120.

34. Pinault, D., and Deschênes, M. (1998). Projection and innervation patterns of individual thalamic reticular axons in the thalamus of the adult rat: a three-dimensional, graphic, and morphometric analysis. J. Comp. Neurol. *391*, 180–203.

35. Hill, S.L., Wang, Y., Riachi, I., Schürmann, F., and Markram, H. (2012). Statistical connectivity provides a sufficient foundation for specific functional connectivity in neocortical neural microcircuits. Proc. Natl. Acad. Sci. USA *109*, E2885–E2894. https://doi.org/10.1073/pnas.1202128109.

36. Morgan, J.L., and Lichtman, J.W. (2020). An individual interneuron participates in many kinds of inhibition and innervates much of the mouse visual thalamus. Neuron *106*, 468–481.e2. https://doi.org/10.1016/j.neuron.2020.02.001.

37. Sherman, S.M. (2004). Interneurons and triadic circuitry of the thalamus. Trends Neurosci. *27*, 670–675. https://doi.org/10.1016/j.tins.2004.08.003.

38. Takeuchi, Y., Osaki, H., Yagasaki, Y., Katayama, Y., and Miyata, M. (2017). Afferent fiber remodeling in the somatosensory thalamus of mice as a neural basis of somatotopic reorganization in the brain and ectopic mechanical hypersensitivity after peripheral sensory nerve injury. eNeuro *4*, ENEURO.0345, 16.2017. https://doi.org/10.1523/ENEURO.0345-16.2017.

39. Çavdar, S., Hacioğlu, H., Şirvanci, S., Keskinöz, E., and Onat, F. (2011). Synaptic organization of the rat thalamus: a quantitative study. Neurol. Sci. *32*, 1047–1056. https://doi.org/10.1007/s10072-011-0606-4.

40. Mineff, E.M., and Weinberg, R.J. (2000). Differential synaptic distribution of AMPA receptor subunits in the ventral posterior and reticular thalamic nuclei of the rat. Neuroscience *101*, 969–982.

41. Liu, X.B., and Jones, E.G. (1999). Predominance of corticothalamic synaptic inputs to thalamic reticular nucleus neurons in the rat. J. Comp. Neurol. *414*, 67–79.

42. Fuhrmann, G., Segev, I., Markram, H., and Tsodyks, M. (2002). Coding of temporal information by activity-dependent synapses. J. Neurophysiol. *87*, 140–148. https://doi.org/10.1152/jn.00258.2001.

43. Haas, J.S., Zavala, B., and Landisman, C.E. (2011). Activity-dependent long-term depression of electrical synapses. Science *334*, 389–393. https://doi.org/10.1126/science.1207502.

44. Landisman, C.E., Long, M.A., Beierlein, M., Deans, M.R., Paul, D.L., and Connors, B.W. (2002). Electrical synapses in the thalamic reticular nucleus. J. Neurosci. *22*, 1002–1009.

45. Lee, S.-C., Patrick, S.L., Richardson, K.A., and Connors, B.W. (2014). Two functionally distinct networks of gap junction-coupled inhibitory neurons in the thalamic reticular nucleus. J. Neurosci. *34*, 13170–13182. https://doi.org/10.1523/JNEUROSCI.0562-14.2014.

46. Long, M.A., Landisman, C.E., and Connors, B.W. (2004). Small clusters of electrically coupled neurons generate synchronous rhythms in the thalamic reticular nucleus. J. Neurosci. *24*, 341–349. https://doi.org/10.1523/JNEUROSCI.3358-03.2004.

47. Crabtree, J.W. (2018). Functional diversity of thalamic reticular subnetworks. Front. Syst. Neurosci. *12*, 41. https://doi.org/10.3389/fnsys.2018.00041.

48. Amsalem, O., Van Geit, W., Muller, E., Markram, H., and Segev, I. (2016). From neuron biophysics to orientation selectivity in electrically coupled networks of neocortical L2/3 large basket cells. Cereb. Cortex N. Y. N *26*, 3655–3668. https://doi.org/10.1093/cercor/bhw166.

49. Born, G., Schneider-Soupiadis, F.A., Erisken, S., Vaiceliunaite, A., Lao, C.L., Mobarhan, M.H., Spacek, M.A., Einevoll, G.T., and Busse, L. (2021). Corticothalamic feedback sculpts visual spatial integration in mouse thalamus. Nat. Neurosci. *24*, 1711–1720. https://doi.org/10.1038/s41593-021-00943-0.

50. Hartings, J.A., Temereanca, S., and Simons, D.J. (2000). High responsiveness and direction sensitivity of neurons in the rat thalamic reticular nucleus to vibrissa deflections. J. Neurophysiol. *83*, 2791–2801. https://doi.org/10.1152/jn.2000.83.5.2791.

51. Nestvogel, D.B., and McCormick, D.A. (2022). Visual thalamocortical mechanisms of waking state-dependent activity and alpha oscillations. Neuron *110*, 120–138.e4. https://doi.org/10.1016/j.neuron.2021.10.005.

52. Urbain, N., Salin, P.A., Libourel, P.-A., Comte, J.-C., Gentet, L.J., and Petersen, C.C.H. (2015). Whisking-related changes in neuronal firing and membrane potential dynamics in the somatosensory thalamus of awake mice. Cell Rep. *13*, 647–656. https://doi.org/10.1016/j.celrep.2015.09.029.

53. Kimura, A. (2017). Robust interactions between the effects of auditory and cutaneous electrical stimulations on cell activities in the thalamic reticular nucleus. Brain Res. *1661*, 49–66. https://doi.org/10.1016/j.brainres.2017.02.005.

54. Soto-Sánchez, C., Wang, X., Vaingankar, V., Sommer, F.T., and Hirsch, J.A. (2017). Spatial scale of receptive fields in the visual sector of the cat thalamic reticular nucleus. Nat. Commun. *8*, 800. https://doi.org/10.1038/s41467-017-00762-7.

55. Destexhe, A., Hughes, S.W., Rudolph, M., and Crunelli, V. (2007). Are corticothalamic "up" states fragments of wakefulness? Trends Neurosci. *30*, 334–342. https://doi.org/10.1016/j.tins.2007.04.006.

56. McCormick, D.A., and Bal, T. (1997). Sleep and arousal: thalamocortical mechanisms. Annu. Rev. Neurosci. *20*, 185–215. https://doi.org/10.1146/annurev.neuro.20.1.185.

57. Steriade, M., McCormick, D.A., and Sejnowski, T.J. (1993). Thalamocortical oscillations in the sleeping and aroused brain. Science *262*, 679–685. https://doi.org/10.1126/science.8235588.

58. Castro-Alamancos, M.A. (2002). Different temporal processing of sensory inputs in the rat thalamus during quiescent and information processing states in vivo. J. Physiol. *539*, 567–578. https://doi.org/10.1113/jphysiol.2001.013283.

59. Castro-Alamancos, M.A. (2002). Properties of primary sensory (lemniscal) synapses in the ventrobasal thalamus and the relay of high-frequency sensory inputs. J. Neurophysiol. *87*, 946–953. https://doi.org/10.1152/jn.00426.2001.

60. Diamond, M.E., Armstrong-James, M., and Ebner, F.F. (1992). Somatic sensory responses in the rostral sector of the posterior group (POm) and in the ventral posterior medial nucleus (VPM) of the rat thalamus. J. Comp. Neurol. *318*, 462–476. https://doi.org/10.1002/cne.903180410.

61. Martin-Cortecero, J., and Nuñez, A. (2014). Tactile response adaptation to whisker stimulation in the lemniscal somatosensory pathway of rats. Brain Res. *1591*, 27–37. https://doi.org/10.1016/j.brainres.2014.10.002.

62. Simons, D.J., and Carvell, G.E. (1989). Thalamocortical response transformation in the rat vibrissa/barrel system. J. Neurophysiol. *61*, 311–330. https://doi.org/10.1152/jn.1989.61.2.311.

63. Mease, R.A., Krieger, P., and Groh, A. (2014). Cortical control of adaptation and sensory relay mode in the thalamus. Proc. Natl. Acad. Sci. USA *111*, 6798–6803. https://doi.org/10.1073/pnas.1318665111.

64. Crandall, S.R., Cruikshank, S.J., and Connors, B.W. (2015). A corticothalamic switch: controlling the thalamus with dynamic synapses. Neuron *86*, 768–782. https://doi.org/10.1016/j.neuron.2015.03.040.

65. Kirchgessner, M.A., Franklin, A.D., and Callaway, E.M. (2020). Context-dependent and dynamic functional influence of corticothalamic pathways to first- and higher-order visual thalamus. Proc. Natl. Acad. Sci. USA *117*, 13066–13077. https://doi.org/10.1073/pnas.2002080117.

66. Steriade, M., Deschênes, M., Domich, L., and Mulle, C. (1985). Abolition of spindle oscillations in thalamic neurons disconnected from nucleus reticularis thalami. J. Neurophysiol. *54*, 1473–1497. https://doi.org/10.1152/jn.1985.54.6.1473.

67. Destexhe, A., McCormick, D.A., and Sejnowski, T.J. (1993). A model for 8-10 Hz spindling in interconnected thalamic relay and reticularis neurons. Biophys. J. *65*, 2473–2477. https://doi.org/10.1016/S0006-3495(93)81297-9.

68. Destexhe, A., Bal, T., McCormick, D.A., and Sejnowski, T.J. (1996). Ionic mechanisms underlying synchronized oscillations and propagating waves in a model of ferret thalamic slices. J. Neurophysiol. *76*, 2049–2070.

69. Golomb, D., Wang, X.J., and Rinzel, J. (1996). Propagation of spindle waves in a thalamic slice model. J. Neurophysiol. *75*, 750–769. https://doi.org/10.1152/jn.1996.75.2.750.

70. Kim, U., Bal, T., and McCormick, D.A. (1995). Spindle waves are propagating synchronized oscillations in the ferret LGNd in vitro. J. Neurophysiol. *74*, 1301–1323.

71. Wang, X.J., Golomb, D., and Rinzel, J. (1995). Emergent spindle oscillations and intermittent burst firing in a thalamic model: specific neuronal mechanisms. Proc. Natl. Acad. Sci. USA *92*, 5577–5581. https://doi.org/10.1073/pnas.92.12.5577.

72. Sanchez-Vives, M.V., and McCormick, D.A. (1997). Functional properties of perigeniculate inhibition of dorsal lateral geniculate nucleus thalamocortical neurons in vitro. J. Neurosci. *17*, 8880–8893.

73. Sohal, V.S., Pangratz-Fuehrer, S., Rudolph, U., and Huguenard, J.R. (2006). Intrinsic and synaptic dynamics interact to generate emergent patterns of rhythmic bursting in thalamocortical neurons. J. Neurosci. *26*, 4247–4255. https://doi.org/10.1523/JNEUROSCI.3812-05.2006.

74. Halassa, M.M., Siegle, J.H., Ritt, J.T., Ting, J.T., Feng, G., and Moore, C.I. (2011). Selective optical drive of thalamic reticular nucleus generates thalamic bursts and cortical spindles. Nat. Neurosci. *14*, 1118–1120. https://doi.org/10.1038/nn.2880.

75. Astori, S., Wimmer, R.D., Prosser, H.M., Corti, C., Corsi, M., Liaudet, N., Volterra, A., Franken, P., Adelman, J.P., and Lüthi, A. (2011). The Ca(V)3.3 calcium channel is the major sleep spindle pacemaker in thalamus. Proc. Natl. Acad. Sci. USA *108*, 13823–13828. https://doi.org/10.1073/pnas.1105115108.

76. Liu, X.-B., Murray, K.D., and Jones, E.G. (2011). Low-threshold calcium channel subunit Ca(v) 3.3 is specifically localized in GABAergic neurons of rodent thalamus and cerebral cortex. J. Comp. Neurol. *519*, 1181–1195. https://doi.org/10.1002/cne.22567.

77. Pellegrini, C., Lecci, S., Lüthi, A., and Astori, S. (2016). Suppression of sleep spindle rhythmogenesis in mice with deletion of CaV3.2 and CaV3.3 T-type Ca(2+) channels. Sleep *39*, 875–885. https://doi.org/10.5665/sleep.5646.

78. Talley, E.M., Cribbs, L.L., Lee, J.H., Daud, A., Perez-Reyes, E., and Bayliss, D.A. (1999). Differential distribution of three members of a gene family encoding low voltage-activated (T-type) calcium channels. J. Neurosci. *19*, 1895–1911.

79. Jahnsen, H., and Llinás, R. (1984). Electrophysiological properties of Guinea-pig thalamic neurones: an in vitro study. J. Physiol. *349*, 205–226.

80. Lüthi, A., and McCormick, D.A. (1998). Periodicity of thalamic synchronized oscillations: the role of Ca2+-mediated upregulation of Ih. Neuron *20*, 553–563.

81. Kim, U., and McCormick, D.A. (1998). Functional and ionic properties of a slow afterhyperpolarization in ferret perigeniculate neurons in vitro. J. Neurophysiol. *80*, 1222–1235. https://doi.org/10.1152/jn.1998.80.3.1222.

82. Aston-Jones, G., and Bloom, F.E. (1981). Activity of norepinephrine-containing locus coeruleus neurons in behaving rats anticipates fluctuations in the sleep-waking cycle. J. Neurosci. *1*, 876–886.

83. Bonjean, M., Baker, T., Lemieux, M., Timofeev, I., Sejnowski, T., and Bazhenov, M. (2011). Corticothalamic feedback controls sleep spindle duration in vivo. J. Neurosci. *31*, 9124–9134. https://doi.org/10.1523/JNEUROSCI.0077-11.2011.

84. Clawson, B.C., Durkin, J., and Aton, S.J. (2016). Form and function of sleep spindles across the lifespan. Neural Plast. *2016*, 6936381. https://doi.org/10.1155/2016/6936381.

85. Kandel, A., and Buzsáki, G. (1997). Cellular-synaptic generation of sleep spindles, spike-and-wave discharges, and evoked thalamocortical responses in the neocortex of the rat. J. Neurosci. *17*, 6783–6797.

86. Lüthi, A. (2014). Sleep spindles where they come from, what they do. Neuroscientist *20*, 243–256. https://doi.org/10.1177/1073858413500854.

87. Timofeev, I., Bazhenov, M., Sejnowski, T.J., and Steriade, M. (2001). Contribution of intrinsic and synaptic factors in the desynchronization of thalamic oscillatory activity. Thalamus Relat. Syst. *1*, 53–69. https://doi.org/10.1016/S1472-9288(01)00004-8.

88. Barthó, P., Slézia, A., Mátyás, F., Faradzs-Zade, L., Ulbert, I., Harris, K.D., and Acsády, L. (2014). Ongoing network state controls the length of sleep spindles via inhibitory activity. Neuron *82*, 1367–1379. https://doi.org/10.1016/j.neuron.2014.04.046.

89. Urbain, N., Fourcaud-Trocmé, N., Laheux, S., Salin, P.A., and Gentet, L.J. (2019). Brain-state-dependent modulation of neuronal firing and membrane potential dynamics in the somatosensory thalamus during natural sleep. Cell Rep. *26*, 1443–1457.e5. https://doi.org/10.1016/j.celrep.2019.01.038.

90. Purcell, S.M., Manoach, D.S., Demanuele, C., Cade, B.E., Mariani, S., Cox, R., Panagiotaropoulou, G., Saxena, R., Pan, J.Q., Smoller, J.W., et al. (2017). Characterizing sleep spindles in 11,630 individuals from the national sleep research resource. Nat. Commun. *8*, 15930. https://doi.org/10.1038/ncomms15930.

91. Cox, R., Schapiro, A.C., Manoach, D.S., and Stickgold, R. (2017). Individual differences in frequency and topography of slow and fast sleep spindles. Front. Hum. Neurosci. *11*, 433. https://doi.org/10.3389/fnhum.2017.00433.

92. Destexhe, A., Contreras, D., Sejnowski, T.J., and Steriade, M. (1994). Modeling the control of reticular thalamic oscillations by neuromodulators. Neuroreport *5*, 2217–2220. https://doi.org/10.1097/00001756-199411000-00003.

93. Osorio-Forero, A., Cardis, R., Vantomme, G., Guillaume-Gentil, A., Katsioudi, G., Devenoges, C., Fernandez, L.M.J., and Lüthi, A. (2021). Noradrenergic circuit control of non-REM sleep substates. Curr. Biol. *31*, 5009–5023.e7. https://doi.org/10.1016/j.cub.2021.09.041.

94. Vyazovskiy, V.V., Achermann, P., Borbély, A.A., and Tobler, I. (2004). The dynamics of spindles and EEG slow-wave activity in NREM sleep in mice. Arch. Ital. Biol. *142*, 511–523.

95. Beierlein, M. (2014). Synaptic mechanisms underlying cholinergic control of thalamic reticular nucleus neurons. J. Physiol. *592*, 4137–4145. https://doi.org/10.1113/jphysiol.2014.277376.

96. Boucetta, S., Cissé, Y., Mainville, L., Morales, M., and Jones, B.E. (2014). Discharge profiles across the sleep–waking cycle of identified cholinergic, GABAergic, and glutamatergic neurons in the pontomesencephalic tegmentum of the rat. J. Neurosci. *34*, 4708–4727. https://doi.org/10.1523/JNEUROSCI.2617-13.2014.

97. McCormick, D.A., and Prince, D.A. (1987). Actions of acetylcholine in the Guinea-pig and cat medial and lateral geniculate nuclei, in vitro. J. Physiol. *392*, 147–165.

98. Bazhenov, M., Timofeev, I., Steriade, M., and Sejnowski, T. (2000). Spiking-bursting activity in the thalamic reticular nucleus initiates sequences of spindle oscillations in thalamic networks. J. Neurophysiol. *84*, 1076–1087. https://doi.org/10.1152/jn.2000.84.2.1076.

99. Brown, J.W., Taheri, A., Kenyon, R.V., Berger-Wolf, T.Y., and Llano, D.A. (2020). Signal propagation via open-loop intrathalamic architectures: a computational model. eNeuro *7*, ENEURO.0441-19.2020. https://doi.org/10.1523/ENEURO.0441-19.2020.

100. Bús, B., Antal, K., and Emri, Z. (2018). Intrathalamic connections shape spindle activity - a modelling study. Acta Biol. Hung. *69*, 16–28. https://doi.org/10.1556/018.68.2018.1.2.

101. Destexhe, A., Contreras, D., Sejnowski, T.J., and Steriade, M. (1994). A model of spindle rhythmicity in the isolated thalamic reticular nucleus. J. Neurophysiol. *72*, 803–818.

102. Destexhe, A., Contreras, D., and Steriade, M. (1998). Mechanisms underlying the synchronizing action of corticothalamic feedback through inhibition of thalamic relay cells. J. Neurophysiol. *79*, 999–1016. https://doi.org/10.1152/jn.1998.79.2.999.

103. Traub, R.D., Contreras, D., Cunningham, M.O., Murray, H., LeBeau, F.E.N., Roopun, A., Bibbig, A., Wilent, W.B., Higley, M.J., and Whittington, M.A. (2005). Single-column thalamocortical network model exhibiting gamma oscillations, sleep spindles, and epileptogenic bursts. J. Neurophysiol. *93*, 2194–2232. https://doi.org/10.1152/jn.00983.2004.

104. McAlonan, K., Cavanaugh, J., and Wurtz, R.H. (2006). Attentional modulation of thalamic reticular neurons. J. Neurosci. *26*, 4444–4450. https://doi.org/10.1523/JNEUROSCI.5602-05.2006.

105. Chen, Z., Wimmer, R.D., Wilson, M.A., and Halassa, M.M. (2015). Thalamic circuit mechanisms link sensory processing in sleep and attention. Front. Neural Circ. *9*, 83. https://doi.org/10.3389/fncir.2015.00083.

106. Rovó, Z., Mátyás, F., Barthó, P., Slézia, A., Lecci, S., Pellegrini, C., Astori, S., Dávid, C., Hangya, B., Lüthi, A., and Acsády, L. (2014). Phasic, nonsynaptic GABA-A receptor-mediated inhibition entrains thalamocortical oscillations. J. Neurosci. *34*, 7137–7147. https://doi.org/10.1523/JNEUROSCI.4386-13.2014.

107. Li, G., Henriquez, C.S., and Fröhlich, F. (2017). Unified thalamic model generates multiple distinct oscillations with state-dependent entrainment by stimulation. PLoS Comput. Biol. *13*, e1005797. https://doi.org/10.1371/journal.pcbi.1005797.

108. Fogerson, P.M., and Huguenard, J.R. (2016). Tapping the brakes: cellular and synaptic mechanisms that regulate thalamic oscillations. Neuron *92*, 687–704. https://doi.org/10.1016/j.neuron.2016.10.024.

109. Lee, K.H., and McCormick, D.A. (1996). Abolition of spindle oscillations by serotonin and norepinephrine in the ferret lateral geniculate and perigeniculate nuclei in vitro. Neuron *17*, 309–321. https://doi.org/10.1016/S0896-6273(00)80162-2.

110. Christensen, J.A.E., Kempfner, J., Zoetmulder, M., Leonthin, H.L., Arvastson, L., Christensen, S.R., Sorensen, H.B.D., and Jennum, P. (2014). Decreased sleep spindle density in patients with idiopathic REM sleep behavior disorder and patients with Parkinson's disease. Clin. Neurophysiol. *125*, 512–519. https://doi.org/10.1016/j.clinph.2013.08.013.

111. Kam, K., Parekh, A., Sharma, R.A., Andrade, A., Lewin, M., Castillo, B., Bubu, O.M., Chua, N.J., Miller, M.D., Mullins, A.E., et al. (2019). Sleep oscillation-specific associations with Alzheimer's disease CSF biomarkers: novel roles for sleep spindles and tau. Mol. Neurodegener. *14*, 10. https://doi.org/10.1186/s13024-019-0309-5.

112. Ktonas, P.Y., Golemati, S., Xanthopoulos, P., Sakkalis, V., Ortigueira, M.D., Tsekou, H., Zervakis, M., Paparrigopoulos, T., Bonakis, A.,

CellPress
OPEN ACCESS

Economou, N.T., et al. (2009). Time–frequency analysis methods to quantify the time-varying microstructure of sleep EEG spindles: possibility for dementia biomarkers? J. Neurosci. Methods *185*, 133–142. https://doi.org/10.1016/j.jneumeth.2009.09.001.

113. Timofeev, I., and Chauvette, S. (2013). The spindles: are they still thalamic? Sleep *36*, 825–826. https://doi.org/10.5665/sleep.2702.

114. Clascá, F., Rubio-Garrido, P., and Jabaudon, D. (2012). Unveiling the diversity of thalamocortical neuron subtypes: thalamocortical neuron diversity. Eur. J. Neurosci. *35*, 1524–1532. https://doi.org/10.1111/j.1460-9568.2012.08033.x.

115. Jones, E.G. (2009). Synchrony in the interconnected circuitry of the thalamus and cerebral cortex. Ann. N. Y. Acad. Sci. *1157*, 10–23. https://doi.org/10.1111/j.1749-6632.2009.04534.x.

116. Acuna-Goycolea, C., Brenowitz, S.D., and Regehr, W.G. (2008). Active dendritic conductances dynamically regulate GABA release from thalamic interneurons. Neuron *57*, 420–431. https://doi.org/10.1016/j.neuron.2007.12.022.

117. Huguenard, J.R., and Prince, D.A. (1992). A novel T-type current underlies prolonged Ca(2+)-dependent burst firing in GABAergic neurons of rat thalamic reticular nucleus. J. Neurosci. *12*, 3804–3817.

118. Deleuze, C., and Huguenard, J.R. (2006). Distinct electrical and chemical connectivity maps in the thalamic reticular nucleus: potential roles in synchronization and sensation. J. Neurosci. *26*, 8633–8645. https://doi.org/10.1523/JNEUROSCI.2333-06.2006.

119. Krahe, T.E., El-Danaf, R.N., Dilger, E.K., Henderson, S.C., and Guido, W. (2011). Morphologically distinct classes of relay cells exhibit regional preferences in the dorsal lateral geniculate nucleus of the mouse. J. Neurosci. *31*, 17437–17448. https://doi.org/10.1523/JNEUROSCI.4370-11.2011.

120. Martinez-Garcia, R.I., Voelcker, B., Zaltsman, J.B., Patrick, S.L., Stevens, T.R., Connors, B.W., and Cruikshank, S.J. (2020). Two dynamically distinct circuits drive inhibition in the sensory thalamus. Nature *583*, 813–818. https://doi.org/10.1038/s41586-020-2512-5.

121. Spreafico, R., Battaglia, G., and Frassoni, C. (1991). The reticular thalamic nucleus (RTN) of the rat: cytoarchitectural, Golgi, immunocytochemical, and horseradish peroxidase study. J. Comp. Neurol. *304*, 478–490. https://doi.org/10.1002/cne.903040311.

122. Borges, F.S., Moreira, J.V.S., Takarabe, L.M., Lytton, W.W., and Dura-Bernal, S. (2022). Large-scale biophysically detailed model of somatosensory thalamocortical circuits in NetPyNE. Front. Neuroinf. *16*, 884245.

123. Li, Y., Lopez-Huerta, V.G., Adiconis, X., Levandowski, K., Choi, S., Simmons, S.K., Arias-Garcia, M.A., Guo, B., Yao, A.Y., Blosser, T.R., et al. (2020). Distinct subnetworks of the thalamic reticular nucleus. Nature *583*, 819–824. https://doi.org/10.1038/s41586-020-2504-5.

124. Wang, Q., Ding, S.-L., Li, Y., Royall, J., Feng, D., Lesnar, P., Graddis, N., Naeemi, M., Facer, B., Ho, A., et al. (2020). The allen mouse brain common coordinate framework: a 3D reference Atlas. Cell *181*, 936–953.e20. https://doi.org/10.1016/j.cell.2020.04.007.

125. Federhen, S. (2012). The NCBI Taxonomy database. Nucleic Acids Res. *40*, D136–D143. https://doi.org/10.1093/nar/gkr1178.

126. Clemente-Perez, A., Makinson, S.R., Higashikubo, B., Brovarney, S., Cho, F.S., Urry, A., Holden, S.S., Wimer, M., Dávid, C., Fenno, L.E., et al. (2017). Distinct thalamic reticular cell types differentially modulate normal and pathological cortical rhythms. Cell Rep. *19*, 2130–2142. https://doi.org/10.1016/j.celrep.2017.05.044.

127. Lam, Y.-W., and Sherman, S.M. (2011). Functional organization of the thalamic input to the thalamic reticular nucleus. J. Neurosci. *31*, 6791–6799. https://doi.org/10.1523/JNEUROSCI.3073-10.2011.

128. Furuta, T., Deschênes, M., and Kaneko, T. (2011). Anisotropic distribution of thalamocortical boutons in barrels. J. Neurosci. *31*, 6432–6439. https://doi.org/10.1523/JNEUROSCI.6154-10.2011.

129. Porrero, C., Rodríguez-Moreno, J., Quetglas, J.I., Smerdou, C., Furuta, T., and Clascá, F. (2016). A simple and efficient in vivo non-viral RNA transfection method for labeling the whole axonal tree of individual adult long-range projection neurons. Front. Neuroanat. *10*, 27. https://doi.org/10.3389/fnana.2016.00027.

130. Shu, S.Y., Ju, G., and Fan, L.Z. (1988). The glucose oxidase-DAB-nickel method in peroxidase histochemistry of the nervous system. Neurosci. Lett. *85*, 169–171. https://doi.org/10.1016/0304-3940(88)90346-1.

131. Rodriguez-Moreno, J., Porrero, C., Rollenhagen, A., Rubio-Teves, M., Casas-Torremocha, D., Alonso-Nanclares, L., Yakoubi, R., Santuy, A., Merchan-Pérez, A., DeFelipe, J., et al. (2020). Area-specific synapse structure in branched posterior nucleus axons reveals a new level of complexity in thalamocortical networks. J. Neurosci. *40*, 2663–2679. https://doi.org/10.1523/JNEUROSCI.2886-19.2020.

132. Jaeger, D. (2000). Accurate reconstruction of neuronal morphology. In Computational Neuroscience: Realistic Modeling for Experimentalists, pp. 159–178.

133. Harris, R.M. (1987). Axon collaterals in the thalamic reticular nucleus from thalamocortical neurons of the rat ventrobasal thalamus. J. Comp. Neurol. *258*, 397–406. https://doi.org/10.1002/cne.902580308.

134. Daigle, T.L., Madisen, L., Hage, T.A., Valley, M.T., Knoblich, U., Larsen, R.S., Takeno, M.M., Huang, L., Gu, H., Larsen, R., et al. (2018). A suite of transgenic driver and reporter mouse lines with enhanced brain-cell-type targeting and functionality. Cell *174*, 465–480.e22. https://doi.org/10.1016/j.cell.2018.06.035.

135. Harris, J.A., Hirokawa, K.E., Sorensen, S.A., Gu, H., Mills, M., Ng, L.L., Bohn, P., Mortrud, M., Ouellette, B., Kidney, J., et al. (2014). Anatomical characterization of Cre driver mice for neural circuit mapping and manipulation. Front. Neural Circ. *8*, 76. https://doi.org/10.3389/fncir.2014.00076.

136. Gong, H., Xu, D., Yuan, J., Li, X., Guo, C., Peng, J., Li, Y., Schwarz, L.A., Li, A., Hu, B., et al. (2016). High-throughput dual-colour precision imaging for brain-wide connectome with cytoarchitectonic landmarks at the cellular level. Nat. Commun. *7*, 12142. https://doi.org/10.1038/ncomms12142.

137. Peng, H., Xie, P., Liu, L., Kuang, X., Wang, Y., Qu, L., Gong, H., Jiang, S., Li, A., Ruan, Z., et al. (2021). Morphological diversity of single neurons in molecularly defined cell types. Nature *598*, 174–181. https://doi.org/10.1038/s41586-021-03941-1.

138. Abdellah, M., Hernando, J., Eilemann, S., Lapere, S., Antille, N., Markram, H., and Schürmann, F. (2018). NeuroMorphoVis: a collaborative framework for analysis and visualization of neuronal morphology skeletons reconstructed from microscopy stacks. Bioinformatics *34*, i574–i582. https://doi.org/10.1093/bioinformatics/bty231.

139. Anwar, H., Riachi, I., Hill, S., Schürmann, F., and Markram, H. (2009). An approach to capturing neuron morphological diversity. In Computational Modeling Methods for Neuroscientists (The MIT Press). https://doi.org/10.7551/mitpress/9780262013277.003.0010.

140. Sholl, D.A. (1953). Dendritic organization in the neurons of the visual and motor cortices of the cat. J. Anat. *87*, 387–406.

141. Ramaswamy, S., Hill, S.L., King, J.G., Schürmann, F., Wang, Y., and Markram, H. (2012). Intrinsic morphological diversity of thick-tufted layer 5 pyramidal neurons ensures robust and invariant properties of *in silico* synaptic connections: comparison of *in vitro* and *in silico* TTL5 synaptic connections. J. Physiol. *590*, 737–752. https://doi.org/10.1113/jphysiol.2011.219576.

142. Destexhe, A., Neubig, M., Ulrich, D., and Huguenard, J. (1998). Dendritic low-threshold calcium currents in thalamic relay cells. J. Neurosci. *18*, 3574–3588.

143. Budde, T., Biella, G., Munsch, T., and Pape, H.C. (1997). Lack of regulation by intracellular Ca2+ of the hyperpolarization-activated cation current in rat thalamic neurones. J. Physiol. *503*, 79–85.

144. Huguenard, J.R., and McCormick, D.A. (1992). Simulation of the currents involved in rhythmic oscillations in thalamic relay neurons. J. Neurophysiol. *68*, 1373–1383.

145. McCormick, D.A., and Pape, H.C. (1990). Properties of a hyperpolarization-activated cation current and its role in rhythmic oscillation in thalamic relay neurones. J. Physiol. *431*, 291–318.

146. Amarillo, Y., Zagha, E., Mato, G., Rudy, B., and Nadal, M.S. (2014). The interplay of seven subthreshold conductances controls the resting membrane potential and the oscillatory behavior of thalamocortical neurons. J. Neurophysiol. *112*, 393–410. https://doi.org/10.1152/jn.00647.2013.

147. Hay, E., Hill, S., Schürmann, F., Markram, H., and Segev, I. (2011). Models of neocortical layer 5b pyramidal cells capturing a wide range of dendritic and perisomatic active properties. PLoS Comput. Biol. *7*, e1002107. https://doi.org/10.1371/journal.pcbi.1002107.

148. Parri, H.R., and Crunelli, V. (1998). Sodium current in rat and cat thalamocortical neurons: role of a non-inactivating component in tonic and burst firing. J. Neurosci. *18*, 854–867.

149. Köhler, M., Hirschberg, B., Bond, C.T., Kinzie, J.M., Marrion, N.V., Maylie, J., and Adelman, J.P. (1996). Small-conductance, calcium-activated potassium channels from mammalian brain. Science *273*, 1709–1714. https://doi.org/10.1126/science.273.5282.1709.

150. Colbert, C.M., and Pan, E. (2002). Ion channel properties underlying axonal action potential initiation in pyramidal neurons. Nat. Neurosci. *5*, 533–538. https://doi.org/10.1038/nn0602-857.

151. Avery, R.B., and Johnston, D. (1996). Multiple channel types contribute to the low-voltage-activated calcium current in hippocampal CA3 pyramidal neurons. J. Neurosci. *16*, 5567–5582.

152. Kole, M.H.P., Hallermann, S., and Stuart, G.J. (2006). Single Ih channels in pyramidal neuron dendrites: properties, distribution, and impact on action potential output. J. Neurosci. *26*, 1677–1687. https://doi.org/10.1523/JNEUROSCI.3664-05.2006.

153. Magistretti, J., and Alonso, A. (1999). Biophysical properties and slow voltage-dependent inactivation of a sustained sodium current in entorhinal cortex layer-II principal neurons: a whole-cell and single-channel study. J. Gen. Physiol. *114*, 491–509.

154. Korngreen, A., and Sakmann, B. (2000). Voltage-gated K+ channels in layer 5 neocortical pyramidal neurones from young rats: subtypes and gradients. J. Physiol. *525*, 621–639. https://doi.org/10.1111/j.1469-7793.2000.00621.x.

155. Reuveni, I., Friedman, A., Amitai, Y., and Gutnick, M.J. (1993). Stepwise repolarization from Ca2+ plateaus in neocortical pyramidal cells: evidence for nonhomogeneous distribution of HVA Ca2+ channels in dendrites. J. Neurosci. *13*, 4609–4621.

156. Rettig, J., Wunder, F., Stocker, M., Lichtinghagen, R., Mastiaux, F., Beckh, S., Kues, W., Pedarzani, P., Schröter, K.H., and Ruppersberg, J.P. (1992). Characterization of a Shaw-related potassium channel family in rat brain. EMBO J. *11*, 2473–2486.

157. Van Geit, W., Gevaert, M., Chindemi, G., Rössert, C., Courcol, J.-D., Muller, E.B., Schürmann, F., Segev, I., and Markram, H. (2016). BluePyOpt: leveraging open source software and cloud infrastructure to optimise model parameters in neuroscience. Front. Neuroinf. *10*, 17.

158. Avanzini, G., de Curtis, M., Panzica, F., and Spreafico, R. (1989). Intrinsic properties of nucleus reticularis thalami neurones of the rat studied in vitro. J. Physiol. *416*, 111–122.

159. Cueni, L., Canepari, M., Luján, R., Emmenegger, Y., Watanabe, M., Bond, C.T., Franken, P., Adelman, J.P., and Lüthi, A. (2008). T-type Ca2+ channels, SK2 channels and SERCAs gate sleep-related oscillations in thalamic dendrites. Nat. Neurosci. *11*, 683–692. https://doi.org/10.1038/nn.2124.

160. Bridson, R. (2007). Fast Poisson disk sampling in arbitrary dimensions. SIGGRAPH Sketches *10*, 1278780–1278807.

161. Tulleken, H. (2009). Poisson disk sampling. Dev. Mamm. http://devmag.org.za/2009/05/03/poisson-disk-sampling/

162. Harris, R.M. (1986). Morphology of physiologically identified thalamocortical relay neurons in the rat ventrobasal thalamus. J. Comp. Neurol. *251*, 491–505. https://doi.org/10.1002/cne.902510405.

163. Pinault, D., Bourassa, J., and Deschênes, M. (1995). The axonal arborization of single thalamic reticular neurons in the somatosensory thalamus of the rat. Eur. J. Neurosci. *7*, 31–40.

164. Kozloski, J., Sfyrakis, K., Hill, S., Schurmann, F., Peck, C., and Markram, H. (2008). Identifying, tabulating, and analyzing contacts between branched neuron morphologies. IBM J. Res. Dev. *52*, 43–55. https://doi.org/10.1147/rd.521.0043.

165. Lee, S.-C., Cruikshank, S.J., and Connors, B.W. (2010). Electrical and chemical synapses between relay neurons in developing thalamus. J. Physiol. *588*, 2403–2415. https://doi.org/10.1113/jphysiol.2010.187096.

166. Cox, C.L., and Sherman, S.M. (2000). Control of dendritic outputs of inhibitory interneurons in the lateral geniculate nucleus. Neuron *27*, 597–610. https://doi.org/10.1016/S0896-6273(00)00069-6.

167. Zhu, J., and Heggelund, P. (2001). Muscarinic regulation of dendritic and axonal outputs of rat thalamic interneurons: a new cellular mechanism for uncoupling distal dendrites. J. Neurosci. *21*, 1148–1159.

168. Shishido, S.-I., and Toda, T. (2017). Temporal patterns of individual neuronal firing in rat dorsal column nuclei provide information required for somatosensory discrimination. Tohoku J. Exp. Med. *243*, 115–126. https://doi.org/10.1620/tjem.243.115.

169. Monconduit, L., Lopez-Avila, A., Molat, J.-L., Chalus, M., and Villanueva, L. (2006). Corticofugal output from the primary somatosensory cortex selectively modulates innocuous and noxious inputs in the rat spinothalamic system. J. Neurosci. *26*, 8441–8450. https://doi.org/10.1523/JNEUROSCI.1293-06.2006.

170. Sherman, S.M., and Koch, C. (1986). The control of retinogeniculate transmission in the mammalian lateral geniculate nucleus. Exp. Brain Res. *63*, 1–20.

171. Mo, C., Petrof, I., Viaene, A.N., and Sherman, S.M. (2017). Synaptic properties of the lemniscal and paralemniscal pathways to the mouse somatosensory thalamus. Proc. Natl. Acad. Sci. USA *114*, E6212–E6221. https://doi.org/10.1073/pnas.1703222114.

172. Sherman, S.M., and Guillery, R.W. (1998). On the actions that one nerve cell can have on another: distinguishing "drivers" from "modulators. Proc. Natl. Acad. Sci. USA *95*, 7121–7126. https://doi.org/10.1073/pnas.95.12.7121.

173. Arsenault, D., and Zhang, Z.w. (2006). Developmental remodelling of the lemniscal synapse in the ventral basal thalamus of the mouse. J. Physiol. *573*, 121–132. https://doi.org/10.1113/jphysiol.2006.106542.

174. Miyata, M., and Imoto, K. (2006). Different composition of glutamate receptors in corticothalamic and lemniscal synaptic responses and their roles in the firing responses of ventrobasal thalamic neurons in juvenile mice. J. Physiol. *575*, 161–174. https://doi.org/10.1113/jphysiol.2006.114413.

175. Warren, R.A., Agmon, A., and Jones, E.G. (1994). Oscillatory synaptic interactions between ventroposterior and reticular neurons in mouse thalamus in vitro. J. Neurophysiol. *72*, 1993–2003. https://doi.org/10.1152/jn.1994.72.4.1993.

176. Zhu, J.J., and Lo, F.S. (1999). Three GABA receptor-mediated postsynaptic potentials in interneurons in the rat lateral geniculate nucleus. J. Neurosci. *19*, 5721–5730.

177. Tsodyks, M.V., and Markram, H. (1997). The neural code between neocortical pyramidal neurons depends on neurotransmitter release probability. Proc. Natl. Acad. Sci. USA *94*, 719–723. https://doi.org/10.1073/pnas.94.2.719.

178. del Castillo, J., and Katz, B. (1954). Quantal components of the end-plate potential. J. Physiol. *124*, 560–573.

179. Korn, H., and Faber, D.S. (1991). Quantal analysis and synaptic efficacy in the CNS. Trends Neurosci. *14*, 439–445. https://doi.org/10.1016/0166-2236(91)90042-s.

180. Antille, N., Courcol, J.D., Abdellah, M., Adaszewski, S.R., Ramaswamy, S., Arsever, S., Atenekeng, G., Bilgili, A., Brukau, Y., Chalimourda, A.,

CellPress
OPEN ACCESS

et al. (2015). The neocortical microcircuit collaboration portal: a resource for rat somatosensory cortex. Front. Neural Circ. *9*, 44. https://doi.org/10.3389/fncir.2015.00044.

181. Cox, C.L., Huguenard, J.R., and Prince, D.A. (1997). Nucleus reticularis neurons mediate diverse inhibitory effects in thalamus. Proc. Natl. Acad. Sci. USA *94*, 8854–8859.
182. Gentet, L.J., and Ulrich, D. (2003). Strong, reliable and precise synaptic connections between thalamic relay cells and neurones of the nucleus reticularis in juvenile rats. J. Physiol. *546*, 801–811.
183. Miyata, M. (2007). Distinct properties of corticothalamic and primary sensory synapses to thalamic neurons. Neurosci. Res. *59*, 377–382. https://doi.org/10.1016/j.neures.2007.08.015.
184. Jurgens, C.W.D., Bell, K.A., McQuiston, A.R., and Guido, W. (2012). Optogenetic stimulation of the corticothalamic pathway affects relay cells and GABAergic neurons differently in the mouse visual thalamus. PLoS One *7*, e45717. https://doi.org/10.1371/journal.pone.0045717.
185. Landisman, C.E., and Connors, B.W. (2007). VPM and PoM nuclei of the rat somatosensory thalamus: intrinsic neuronal properties and corticothalamic feedback. Cerebr. Cortex *17*, 2853–2865. https://doi.org/10.1093/cercor/bhm025.
186. Reichova, I., and Sherman, S.M. (2004). Somatosensory corticothalamic projections: distinguishing drivers from modulators. J. Neurophysiol. *92*, 2185–2197. https://doi.org/10.1152/jn.00322.2004.
187. Barros-Zulaica, N., Rahmon, J., Chindemi, G., Perin, R., Markram, H., Muller, E., and Ramaswamy, S. (2019). Estimating the readily-releasable vesicle pool size at synaptic connections in the neocortex. Front. Synaptic Neurosci. *11*, 29.
188. Ecker, A., Romani, A., Sáray, S., Káli, S., Migliore, M., Falck, J., Lange, S., Mercer, A., Thomson, A.M., Muller, E., et al. (2020). Data-driven integration of hippocampal CA1 synaptic physiology in silico. Hippocampus *30*, 1129–1145. https://doi.org/10.1002/hipo.23220.
189. Häusser, M., and Roth, A. (1997). Estimating the time course of the excitatory synaptic conductance in neocortical pyramidal cells using a novel voltage jump method. J. Neurosci. *17*, 7606–7625.
190. Deleuze, C., and Huguenard, J.R. (2016). Two classes of excitatory synaptic responses in rat thalamic reticular neurons. J. Neurophysiol. *116*, 995–1011. https://doi.org/10.1152/jn.01121.2015.
191. Sarid, L., Bruno, R., Sakmann, B., Segev, I., and Feldmeyer, D. (2007). Modeling a layer 4-to-layer 2/3 module of a single column in rat neocortex: interweaving in vitro and in vivo experimental observations. Proc. Natl. Acad. Sci. USA *104*, 16353–16358. https://doi.org/10.1073/pnas.0707853104.
192. Jahr, C.E., and Stevens, C.F. (1990). Voltage dependence of NMDA-activated macroscopic conductances predicted by single-channel kinetics. J. Neurosci. *10*, 3178–3182.
193. Ulrich, D., and Huguenard, J.R. (1997). Nucleus-specific chloride homeostasis in rat thalamus. J. Neurosci. *17*, 2348–2354.
194. Hines, M.L., and Carnevale, N.T. (1997). The NEURON simulation environment. Neural Comput. *9*, 1179–1209. https://doi.org/10.1162/neco.1997.9.6.1179.
195. Kumbhar, P., Hines, M., Fouriaux, J., Ovcharenko, A., King, J., Delalondre, F., and Schürmann, F. (2019). CoreNEURON : an optimized compute engine for the NEURON simulator. Front. Neuroinf. *13*, 63.
196. Minnery, B.S., Bruno, R.M., and Simons, D.J. (2003). Response transformation and receptive-field synthesis in the lemniscal trigeminothalamic circuit. J. Neurophysiol. *90*, 1556–1570. https://doi.org/10.1152/jn.00111.2003.
197. Pauzin, F.P., and Krieger, P. (2018). A corticothalamic circuit for refining tactile encoding. Cell Rep. *23*, 1314–1325. https://doi.org/10.1016/j.celrep.2018.03.128.

STAR★METHODS

KEY RESOURCES TABLE

REAGENT or RESOURCE	SOURCE	IDENTIFIER
**Deposited data**		
Microcircuit Portal	https://identifiers.org/bbkg:thalamus/studios/e9ceee28-b2c2-4c4d-bff9-d16f43c3eb0f	N/A
**Experimental models: Organisms/strains**		
Mouse: GAD67-eGFP	Laboratory of Sensory Processing, EPFL	N/A
Mouse: C57Bl/6J	Charles River Laboratories, France	N/A
Mouse: SSt-Cre; Ai139	JAX Strain 013,044, USA	MGI Ref ID J:151,755
**Software and algorithms**		
Blue Brain Nexus software	https://www.w3.org/standards/semanticweb/data	RRID: SCR_022029
NeuroM	https://www.w3.org/standards/semanticweb/data	N/A
eFEL	https://www.w3.org/standards/semanticweb/data	N/A
Janelia MouseLight Project	http://ml-neuronbrowser.janelia.org/	RRID: SCR_016668;
Mouse Genomics Information database	http://www.informatics.jax.org/downloads/reports/MGI_Strain.rpt	N/A
Data type schemas	http://neuroshapes.org/	N/A
BBP Cell Atlas	https://portal.bluebrain.epfl.ch/resources/models/cell-atlas/	RRID: SCR_019266
InterLex		RRID: SCR_016178
Allen Common Coordinate Framework	https://www.zotero.org/google-docs/?MGapw0	RRID: SCR_020999
Neocortical Microcircuit Portal	https://www.zotero.org/google-docs/?TzYZzH	RRID: SCR_022032

RESOURCE AVAILABILITY

Lead contact

Further information and requests for resources should be directed to and will be fulfilled by the lead contact, Sean Hill (sean.hill@epfl.ch).

Materials availability

Experimental data, model entities and metadata are made available in the Thalamoreticular Microcircuit Portal (https://identifiers.org/bbkg:thalamus/studios/e9ceee28-b2c2-4c4d-bff9-d16f43c3eb0f). The portal includes data and model entities, including single neuron models, circuit files in SONATA format[1] and simulation output.

All data were integrated, and aligned to FAIR principles (Findible, Accessible, Interoperable, Reusable) using the Blue Brain Nexus software (RRID:SCR_022029). At the center of Blue Brain Nexus lies a knowledge graph which supports W3-standard "linked data" (https://www.w3.org/standards/semanticweb/data) storage and indexing. In the context of the knowledge graph, W3C (World Wide Web Consortium) Shapes Constraint Language (https://www.w3.org/TR/shacl/) was used to define FAIR data models (i.e. 'shape' the data and apply constraints). To support indexation of the datasets, specific data models were developed. Each individual data type was modeled according to a schema, available from Neuroshapes (http://neuroshapes.org/), to ensure standardization across different projects. This process made sure existing schemas, semantic markups, existing ontologies and controlled vocabularies were used.

For each data type, a minimum set of metadata was required to guarantee reusability of the data. We provide a concrete example of integration for an exemplar dataset consisting of neuron morphological reconstructions.

1. Identification of the dataset: 99 morphological reconstructions collected from acute brain slices through whole-cell patch clamp recording and biocytin filling, stored in.asc Neurolucida (rrid:SCR_001775) file format;
2. Identification of the metadata: A spreadsheet containing all the information related to the specimen, experimental protocol, date of the experiment, human agents involved in the experiment and reconstruction;
3. Creation of a data model: A schematic was developed according to the W3C PROV-O specification (https://www.w3.org/2011/prov/wiki/Diagrams), describing how the morphology was obtained in the form of a provenance graph;
4. Vocabulary and ontologies to integrate the dataset and metadata: Cell type terms from InterLex (rrid:SCR_016178) were used. InterLex is a dynamic lexicon of biomedical terms. For brain region, terms from the Allen Common Coordinate Framework version 3 were used[124](rrid:SCT_020999). To store species information, the NCBI organismal classification was used.[125]

For storing information about strain, the Mouse Genomics Informatics database (http://www.informatics.jax.org/downloads/reports/MGI_Strain.rpt) was used. To store sex information, the Phenotype And Trait Ontology was used.

Data and code availability

- The experimental data, including neuron morphologies, *in vitro* electrophysiological recordings, cell and bouton densities are all available under an open access license on the Thalamoreticular Microcircuit Portal (https://identifiers.org/bbkg:thalamus/studios/e9ceee28-b2c2-4c4d-bff9-d16f43c3eb0).
- The Thalamoreticular Microcircuit Portal (https://identifiers.org/bbkg:thalamus/studios/e9ceee28-b2c2-4c4d-bff9-d16f43c3eb0) includes code for the ion channel models, single neuron models, synapse models, circuit files in SONATA format,[1] simulation output.
- Any additional information required to reanalyze the data reported in this paper is available from the lead contact upon request.

EXPERIMENTAL MODEL AND SUBJECT DETAILS

Experimental data were collected in conformity with the Swiss Welfare Act and the Swiss National Institutional Guidelines on Animal Experimentation for the ethical use of animals. The Swiss Cantonal Veterinary Office approved the project following an ethical review by the State Committee for Animal Experimentation.

In vitro-stained TC and Rt morphologies were obtained from P12-35 mice (GAD67-eGFP or C57Bl/6J strains).

In vivo-stained TC and Rt morphologies were obtained from C57Bl/6J 3 month-old male mice.

In vivo labeling of reticular neurons were employed in SSt-Cre; Ai139 P54 mice.

Electrophysiological data for TC and Rt neurons and interneurons (INs) were characterized *in vitro* from brain slices of P12-35 GAD67-eGFP or C57Bl/6J mice.

For all mouse lines, if not otherwise specified, both male and female subjects were used.

METHOD DETAILS

Constraining and validating the model with experimental data

The model microcircuit was built by constraining and validating it at multiple levels, using experimental data and algorithmic approaches, based on methods published previously.[24] For validation we performed direct comparison of the model properties with experimental measurements that were not used during the model building steps. Before describing the details of the reconstruction, validation and simulations, we provide a list of data used for constraining the model, the validation data and further validations at the network level.

Experimental data used to constrain the model

The following experimental data was used to constrain the model, further details on the experimental procedures and literature references are provided below.

- Three-dimensional reconstructions of neuron morphologies, from *in vitro* and *in vivo* labeling
- Ion channel kinetic parameters
- Electrophysiological data from *in vitro* patch-clamp recordings (current step stimuli)
- Neuron densities
- Fraction of inhibitory and excitatory neurons
- Fraction of electrical types for each morphological type
- Axonal bouton densities (i.e. number of boutons per axonal unit length)
- Volumetric densities of lemniscal boutons (number of boutons per unit volume)
- Ratio of corticothalamic to lemniscal bouton densities and ratio of corticothalamic to thalamocortical bouton densities (volumetric data from the literature)
- Postsynaptic potential amplitudes and their change in response to trains of presynaptic inputs from *in vitro* paired-recordings (short-term plasticity protocols)
- Number of neurons connected through gap junctions
- Synaptic current kinetic parameters

Experimental data used for model validation

The following experimental measurements were not used for constraining the model during the building process but were used for validation.

- Electrophysiological data from *in vitro* patch-clamp recordings (current ramps and noise)

- Number of synapses per connection between interneurons and thalamocortical neurons (*i.e*. number of synapses between each pair of neurons)
- Synaptic convergence onto reticular neurons
- Postsynaptic potential amplitudes (different subset of neuron pairs than the ones used to constrain the model)
- Coefficient of variation of first postsynaptic potential amplitudes
- Distance-dependent gap junction connectivity between reticular neurons
- Gap junctions coupling coefficients

Validations at the network level

We identified the following network responses during simulated activity as a general validation of the reconstruction process.

- Spontaneous *in vivo*-like activity, characterized by uncorrelated firing and low firing rates in TC and Rt cells[49–51]
- Evoked activity with simulated sensory input in TC as well Rt cells[50]
- Adaptation to repeated sensory stimuli at different frequencies[59]
- Corticothalamic inputs counterbalance sensory adaptation[63]
- Increased thalamic bursts after brief stimulation of the reticular nucleus and evoked spindle-like oscillations[74]
- Initiation of spindle-like oscillations during cortical UP-states[55–57]

Reconstructing the morphological diversity of neurons

Reconstruction of morphologies

A subset of 3D reconstructions of biocytin-stained thalamocortical (TC) neurons, reticular thalamic (Rt) neurons and thalamic interneurons (IN) were obtained from *in vitro* patch-clamp experiments from 300 μm slices of P12-35 mice (GAD67-eGFP or C57Bl/6J strains) as previously described.[24,26] During the electrophysiological recordings neurons were stained intracellularly with biocytin. *In vitro*-stained neurons were mainly located in primary somatosensory nuclei (VPL, and ventral posteromedial nucleus - VPM) and the somatosensory sector of the reticular nucleus.[34,126,127] Although the main focus of our model was the VPL, we found that specifically targeting this nucleus was challenging. To increase the available experimental data we thus included recordings from the VPM. Reconstructions used the Neurolucida system (MicroBrightField) and were corrected for shrinkage along the thickness of the slice. Shrinkage along other dimensions was taken into account during the unraveling step (see below). Dendrites were reconstructed with a 100× magnification (oil immersion objective) and axons at 60× (water immersion objective).

In vivo-stained TC and Rt morphologies were obtained through different experimental techniques. In some cases, neurons were labeled by injection of replication-defective Sindbis virus particles in the thalamus or Rt nucleus in C57Bl/6J adult mice[128] or electroporation of RNA of the same virus.[129] The virus labeled the membrane of the neurons thanks to a palmitoylation signal linked to a green fluorescent protein (GFP). Brains were cut in 50 μm serial sections and immunostained against GFP and enhanced with glucose oxidase-nickel staining.[130] Neurons were reconstructed from sequentially-ordered slices under bright-field optics using the Neurolucida system (MicroBrightField). The complete method is described elsewhere.[131]

In vivo-labelled TC morphologies were obtained from the Janelia Mouselight project, from sparsely-labeled adult C57/BL6 mice brains; the method is described in detail elsewhere[25] and summarized here. Brains were then delipidated, fluorescence was enhanced by immunolabeling and imaged with a 40× oil-immersion objective. This procedure generated large datasets of high-resolution image stacks. The 3D reconstructions were conducted combining semi-automated segmentation of the neurites, human annotation and quality control. Janelia Mouselight reconstructions lacked diameter variations in their neurites, which is important for accurate electrical modeling of neurons.[132] For this reason, we only used their axons in order to increase the variability of our axonal reconstructions. We obtained 96 morphologies whose soma was located in the thalamus and we visually inspected their shape along with 3D meshes of the reticular nucleus of the thalamus using the Janelia MouseLight Project (RRID:SCR_016668; http://ml-neuronbrowser.janelia.org/). Since most thalamocortical neurons project to the Rt on their path to the cortex,[114,127] we selected the 41 morphologies which gave off collaterals in the reticular nucleus. We assumed that neurons without collaterals in the Rt were partially labeled and/or reconstructed, since those collaterals are often very thin.[133] Given the limited number of reconstructed morphologies of neurons in VPL and VPM in the Janelia MouseLight dataset, we included 27 axons (with collaterals to Rt) from other thalamic nuclei. To ensure that the connectivity would not be impacted, we analyzed the geometrical properties of the Rt collaterals and found that the difference within the same nucleus was as high as the difference between nuclei.

For *in vivo* labeling of reticular neurons virus injections for sparse labeling of whole brain neuron morphologies were employed in SSt-Cre; Ai139 adult mice.[134,135] Brains were imaged using fluorescence micro-optical sectioning tomography (fMOST).[136] Neurons were manually reconstructed from high resolution image stacks obtained after slicing. Further details of the method are available in related publications.[137]

Morphology analysis, alignment and visualization

Raw morphological data did not have a common orientation along a principal axis, which is necessary to place them in the microcircuit volume according to biologically-plausible constraints (see below). We thus computed a rotation matrix so that the principal axis of the morphology was parallel to the vertical axis of the microcircuit. The principal axis of TC morphologies was the one

connecting the center of the soma and the center of mass of the axon collaterals in the Rt nucleus (see below). For Rt neurons, the principal axis connected the soma and the center of mass of the axonal arborization in the thalamus. After rotating the morphologies, we visually validated the results. Rotation of the INs was not performed, since no orientation information relative to known landmarks was available.

For morphology analysis we used the open-source library NeuroM (https://github.com/BlueBrain/NeuroM). To identify the TC axon collaterals projecting to the Rt we selected the morphological sections which had branch order $\geq$ 1 and path distance from the soma <2,500 μm and visually validated the results. For some morphologies, we selected those having path distance $\leq$ 2,000 μm, because some TC neurons have collaterals projecting to other subcortical regions (e.g striatum), see Clascá et al.[114]

Raw morphological data were algorithmically corrected for slicing artifacts and processed to generate a large pool of unique morphologies for building the microcircuit and connectivity. Spurious sections, which were accidentally introduced during manual reconstruction, were identified as those having 0 μm diameter and removed. The details are described in Supplemental Experimental Procedure of the neocortical microcircuit model, and summarized below.[24]

The morphology images in Figure 2 were created using NeuroMorphoVis.[138]

Unraveling morphologies

Since we found that 3D reconstructions from *in vitro*-stained neurons had increased tortuosity in their dendrites as a result of tissue shrinkage, we unraveled them using an existing algorithm.[24] This process resulted in an increase of the reach of the morphologies, while preserving the original length of the branches. Briefly, unraveling was performed by sections and for each section a sliding window composed of a given number of successive points was created. The number of points in the sliding window (N) was the only parameter of the algorithm and we found that N = 5 previously used performed well on thalamic morphologies. The general direction of the points in the window was computed using principal component analysis (PCA). The segment at the middle of the window was then aligned along this direction. It meant that its direction was set to the one of the sliding windows but it retained its original length. The sliding window was moved over all points of the section and the algorithm was applied to all sections.

Repairing morphologies

Most of the *in vitro*-stained morphologies were truncated at slice edges and in the case of some TC morphologies, which have very dense dendritic arborization, this resulted in a significant decrease in dendritic mass. We applied an existing algorithm[24,139] to repair missing dendritic branches. First, the algorithm detects cut points on the XY plane, i.e. the plane parallel to the slice, along the Z direction (parallel to the slice thickness). The 3D coordinate system was centered on the morphology soma. Although the algorithm was designed to detect cut points on two planes, we found that our morphologies were truncated on the top plane. We improved the algorithm by searching the cut points before unraveling the morphologies and updated their position during the unraveling step. Cut detection required a tolerance parameter to detect terminal points within a certain distance from maximum Z extents. We found that 15 μm gave the most accurate results by visual inspection of the morphology. Some terminal points were then tagged cut points and dendrites were repaired.

The dendrite repair process created new dendritic sections starting at the identified cut points. Dendrite repair did not aim to recover the initial morphology, but rather recreated it in a statistical manner, under the assumption of statistical symmetry of the morphology. This method analyzed the behavior of intact branches as a function of branch order and euclidean distance from the soma. For each branch order, probability density clouds of branch continuation, bifurcation or termination were calculated in a series of concentric spheres.[140] At each cut point, the behavior of the branch was sampled according to the calculated probabilities. The factor governing the direction of the re-grown branches was adjusted to achieve final branches tortuosity comparable with our experimental data. To address neurite swelling artifacts at cut points, the diameters of the re-grown branches were set to the average diameter of the last section.

Morphology diversification

We increased the variability of the reconstructed and repaired morphologies to ensure robust and invariant connectivity patterns.[35,141] We followed a previously published method[24] to generate a unique branching pattern for each morphology, while maintaining the general morphological and electrical structure for each m-type. In summary, branch lengths and rotations at each bifurcation point were varied according to random numbers drawn from Gaussian distributions with mean 0% and standard deviation 20% for branch lengths and mean 0° and standard deviation 20° for branch rotations. A sample of the resulting morphologies was visually validated, and we did not find significant alterations of their structure for any of the m-types (Figure S1).

We then applied a mix-and-match procedure to maximize the utilization of good morphological reconstruction data. This procedure divided dendrites from axons and allowed us to combine good dendritic reconstructions of TC and Rt dendrites from *in vitro* and *in vivo*-stained neurons and good axonal reconstructions from *in vivo*-stained neurons. *In vitro*-stained neurons typically lacked reconstruction of the full axon due to the slicing procedure and/or poor labeling. For each morphology, we manually annotated which dendrites and axons were to be kept. The decision in most cases depended on the labeling method (*in vitro* vs. *in vivo*).

To increase the probability that *in vivo*-stained morphologies and in particular the axons of TC and Rt morphologies were compatible with the microcircuit dimensions (see below) we duplicated and scaled the morphologies along their principal axis (Y axis) by ± 2.5%.

Reconstructing the electrical diversity of neurons

Electrophysiological data

The firing patterns of TC, Rt neurons and interneurons (INs) were characterized *in vitro* from brain slices of P12-35 GAD67-eGFP or C57Bl/6J mice and expert-classified into five electrical types (see results). The detailed electrophysiological protocol has been published elsewhere.[26] Neurons were sampled from the ventrobasal complex of the thalamus (VPL and VPM nuclei) and the somatosensory sector of the reticular nucleus.[126,127]

We used responses to step-like currents to build electrical models, ramp and noise currents to validate them,[26] along with excitatory postsynaptic-like currents (EPSC) injected into the dendrites. All the recordings were corrected for liquid junction potential by subtracting 14 mV from the recorded voltage.

Neuron models

Multicompartmental conductance-based models employed 3D morphological reconstructions. Active ion currents and a simple intracellular calcium dynamics model were distributed in the somatic, dendritic and axonal compartments. Only the axonal initial segment (AIS) – not the complete axon – was modeled.[24] The axons were substituted by a 60 μm stub constituted by two sections, five segments each. For each segment, the diameter was extracted from the original axon in order to preserve its tapering. Morphologies were divided into compartments of 40 μm maximal length. Specific membrane capacitance was set to 1 $\mu F/cm^2$ and specific intracellular resistivity to 100 Ωcm.

Ion channel models

We included ion current models whose kinetics were obtained from previously published ion current models or published experimental data. All ion channel models were corrected for liquid junction potential and for simulation at different temperatures whenever possible. Simulation temperature was always set to 34°C.

The details of the ion channel kinetics and calcium dynamics used for low-threshold bursting neurons (TC and Rt) have been described elsewhere[26] and are summarized here. The type of ionic currents present in TC and Rt were: transient sodium current, delayed potassium current and low-threshold calcium from a previous model of TC neurons[142]; h-current model (I_h) was built from published data[26,143–145]; persistent sodium, based on an existing models[146,147] and published data,[148] A-type transient potassium was taken from an existing model,[146] based on published data[144]; high-threshold calcium was based on published models and data,[144,146] SK-type calcium-activated potassium, was taken from previous models and published data.[147,149] Intracellular calcium dynamics was modeled with an exponential decay mechanism that linked low-threshold and high-threshold calcium currents to the calcium-activated potassium.

Since interneurons had firing patterns similar to cortical ones, we used the same ion channel models of the cortical microcircuit model,[24] which were based on existing models or published data. The type of ionic currents were transient sodium,[150] low-threshold calcium,[151] h-current,[152] persistent sodium,[153] transient potassium,[154] high-threshold calcium[155] and potassium Kv3.1.[156] The reversal potential of sodium, potassium and h-current were set to 50 mV, −90 mV and −43 mV, respectively.

Ion channel models were distributed uniformly and with different peak conductance values for somatic, dendritic and axonal compartments, except for the h-current in the interneurons, whose distribution increased exponentially from the soma to the dendrites.[24]

Optimization of neuron models

Five electrical models (e-models), corresponding to each electrical-type (e-type), were fitted using a multiobjective optimization algorithm using the Python library BluePyOpt.[26,157] The free parameters of the model were the peak conductances of the different mechanisms, parameters of the intracellular calcium dynamics (time constant of decay and percent of free calcium, *gamma*) and the reversal potential of the passive mechanism that contributes to the resting membrane potential. Each e-model was fitted with an exemplar morphology.

The optimization objectives were the electrical features extracted from the electrophysiological recordings. All modeled neurons included transient sodium, persistent sodium, A-type transient potassium, delayed potassium, low-threshold calcium, high-threshold calcium, calcium-activated potassium (SK-type), h-current ion channel currents. All cellular compartments (i.e. somata, dendrites and axon initial segments) contained active membrane mechanisms. Rt_RC e-models followed the same approach as for VPL_TC cells, as previously published,[26] with the additional electrical features to quantify the deeper post-burst afterhyperpolarization observed in Rt_RCs.[158,159] To validate and test the generalization of the neuron models, we used features from current stimuli not used during the optimization phase.

The detailed experimental protocol and the type of current stimuli and features are described elsewhere,[26] and summarized here. For all the e-types, two hyperpolarizing steps (−20/−40% and −120/−140% of the threshold current) were used to constrain passive properties (input resistance, resting membrane potential) and current activated by hyperpolarization, e.g. h-current (sag amplitude). Three levels of depolarizing steps (150%, 200%, 250% of the threshold current) were used to constrain firing pattern (adaptation index or inverse of the first and last interspike intervals, spike count, mean frequency) and spike shape-related features (action potential amplitude, depth of the after-hyperpolarization, action potential duration). All these protocols were applied in combination with a hyperpolarizing holding current (to reach stable membrane potential of −84 mV, after liquid junction potential correction).

When low-threshold bursting cells are hyperpolarized compared to their resting membrane potential and then stimulated, they fire stereotypical low-threshold bursts. One step (200% of firing threshold) on top of a hyperpolarizing current was therefore used to constrain the bursting response, while three depolarizing steps on top of a depolarizing holding current (to reach −64 mV) were used to constrain the tonic firing responses, as explained above. For reticular neurons, a new feature (*initburst_sahp*) was added for the afterhyperpolarization after the burst. For Rt and TC cells, two additional protocols without any current injection or only holding currents were used to ensure that the e-models were not firing without stimulus or with the holding current only.

Electrical features from the experimental recordings and model traces were extracted using the open source library eFEL (https://github.com/BlueBrain/eFEL).

We considered a model a good fit to the experimental data if all the feature errors (i.e. the Z-scores) were below 3.

Quality assurance of morpho-electrical models

After fitting the five e-models, they were combined with the 92,970 morphologies generated as output of the morphology diversification step. An automated pipeline tested the e-models in combination with the different morphologies (me-models) and filtered out those that deviated significantly from the experimental electrical features. To decide which me-model was to be accepted, we used the repaired exemplar morphology (i.e. the morphology used during the optimization, after being repaired) as a benchmark: a me-model passed if it had all the feature errors were below 5 standard deviations of the repaired exemplar.[24] To account for the input resistance given by the different morphologies, we devised an algorithm, based on binary search, to find the appropriate holding and threshold current for each me-model.[26]

In addition, we first ran this pipeline on a small subset of the morphologies generated after morphology repair. In this way, we could visually inspect if the accepted me-models were generating biologically plausible firing behavior and the reasons why other me-models had high feature errors. In some cases, after inspecting the me-model voltage responses, we set less stringent criteria on some features, to ensure that we had enough different me-models for building the microcircuit. At the same time, we set more stringent criteria to reject me-models that were active without any input, since we did not find neurons that were spontaneously active in our experimental recordings.

Measuring neuron density

Immunohistochemistry of Rt and VPL for cell counting

We complemented the neuron densities values from the Blue Brain Cell Atlas[32] (RRID:SCR_019266) by counting neurons in adult mouse brain slices. The brain was cryosliced at 50 μm on the sagittal plane and stained following standard immunohistological procedures with antibodies anti-GABA (for inhibitory neurons), anti-NeuN (for neurons) and DAPI (for all cells), using an existing protocol.[24] The slices were imaged with a confocal microscope (Zeiss, 710). The immunohistology and imaging of the region of interest (ROI) was completed for one P21 C57B1/6J mouse.

Semi-automated cell counting and cell densities

The images were aligned to the Allen Reference Atlas to create proper boundaries for the Rt and VPL. We used Imaris software (Bitmap) to create the ROI, for counting the neurons and to estimate the volume for density calculation. For a chosen ROI, the software detected the difference of signal intensity, created a 3D shape around the detected cells and extracted statistics (e.g. count, positions) following given parameters. These parameters were defined by running multiple trials so that the results from semi-automated cell counting were as close as possible to those from manual cell counting. The semi-automated counting method results in very low error rates compared to manual counting (2.25%) and is less time consuming. A 3D shape of the entire ROI was created in order to extract the volume for density calculation. Neuron densities were calculated as the ratio between neuron counts in a ROI and the volume as calculated in Imaris for each slice. For modeling we used the average cell densities for Rt and VPL neurons.

Reconstructing the dimensions and structure of a thalamoreticular microcircuit

Since the thalamus does not have a clear laminar structure, we approximated a thalamic microcircuit as a cylindrical volume having its base parallel to a portion of the Rt and its vertical dimension (y axis) running through the VPL and Rt (see Figure 1C).

The horizontal dimensions of the microcircuit were calculated from the density of dendritic fibers at the center of the circuit, following an approach published previously.[24] For each m-type, we began by considering all the morphologies (after repairing them) that had their somata located within 25 μm from the circuit center on the horizontal plane (XZ). We then increased the maximal distance in steps of 25 μm which resulted in an increase of dendritic densities at the center. The microcircuit horizontal dimension (radius) resulting from this process was 294 μm, corresponding to the distance where 95% of the asymptotical maximal density of reticular neuron dendrites was reached. As a comparison, considering only thalamocortical cell morphologies would have resulted in a circuit with radius 125 μm, while considering only interneurons the radius would have been 279 μm.

We used hexagonal boundaries with the same area as the resulting circle to facilitate tiling of multiple microcircuits, while keeping asymmetrical edge effects minimal. The resulting side of the hexagon was 323 μm and the longest diagonal (vertex-to-vertex) measured 646 μm.

To calculate the vertical dimension of the microcircuit, we extracted a 3D subvolume within the VPL and the Rt. We started from the thalamus parcellation of the Allen Brain Atlas version 3 (25 μm resolution).[29] A spherical coordinate system was fitted to the volume of the Rt, which can be approximated by a spheroidal surface. We chose a ROI located approximately in the middle of the VPL nucleus and computed the probability distribution of widths in the ROI for the VPL and Rt. The widths were calculated along the radius of the

spherical coordinate system. The resulting thickness corresponds to the median of the distributions, which was 550 μm for the VPL and 250 μm for the Rt.

Soma positions and me-type model assignment

The horizontal and vertical extents resulted in a microcircuit having the shape of a hexagonal prism, that was 646 μm wide (at the widest point) and 800 μm high; 69% of the volume was occupied by the VPL and 31% by the Rt. This volume was then populated by defining somata positions according to the experimentally measured neuron densities in the Rt and VPL. The positions were distributed according to an algorithm based on Poisson disc sampling.[160,161] This algorithm avoids clustering normally obtained with sampling according to uniform distributions, by using a parameter for the minimum distance between points. To calculate the minimum distance, we used the cell densities to calculate the expected number of cell positions per voxel. Each soma position was assigned an m-type according to the excitatory/inhibitory fractions and an electrical model in agreement with the me-types composition (Figures 1E–1G). Moreover, each position was associated with a random rotation around the y axis to be applied to each morphology.

Morphology placement

Our pool of experimental morphologies and the ones derived from the morphology diversification process contained morphologies with different sizes and shapes. Moreover, it contained TC morphologies whose somata was not located in the VPL nucleus and Rt morphologies whose axons were not arborizing in the VPL nucleus. We adapted a placement scoring algorithm[24] to ensure that each position was assigned a suitable morphology considering its geometrical properties and the microcircuit vertical dimension.

We thus defined placement rules that took into account the known properties of Rt and TC neurons' arborizations relative to the anatomical boundaries of thalamic nuclei.[162,163] Each reconstruction of TC and Rt neuron morphologies was manually annotated according to the placement rules. For TC cells, we identified the axonal arborization projecting to the Rt (and that should be located in the Rt part of the model). For Rt cells, the densest part of axonal arborization was annotated, which should be located in the VPL. For IN, the only constraint is that the full morphology should be contained within the VPL and not crossing into the Rt.[36] Each annotation was automatically carried over during the unraveling, repairing and diversification steps. Moreover, we included a stricter rule to avoid that Rt morphologies were located outside the top of the circuit boundary, with a 30 μm tolerance.

Given the placement rules, each morphology was assigned a score based on the microcircuit position and the constraints set by the placement rules.[24]

Generating different microcircuit instances

We created five different microcircuit instances to assess the model robustness to different input parameters. The experimentally-measured cell densities were jittered by +/− 5%, resulting in microcircuits with different total number of neurons and number of neurons for each m-type (see Figure 1F).

Reconstructing the synaptic connectivity of a thalamoreticular microcircuit

Connectivity based on morphological appositions

After placing the morphologies in the 3D microcircuit volume we generate the first version of the connectivity by detecting zones of geometrical overlap ("touches") using an existing touch detection algorithm.[24,164] Briefly, this algorithm sub-divided the circuit 3D space into sub-volumes ensuring that each sub-volume contained the same amount of data, i.e. the same number of morphological segments. Each sub-volume was processed in parallel on different cores and written in parallel to disk. All geometrical overlaps were considered as touches if their distance was smaller or equal to 1 μm ("touch distance").

Touches were then filtered according to biological rules: touches were allowed between all m-types, except between interneurons and reticular cells, because interneurons are only located in the thalamus and are not expected to have neurites extending into the reticular nucleus.[36] Touches between VPL neurons were removed, in agreement with experimental findings showing that excitatory connections between TC neurons disappear during development.[165]

Interneurons also form axonal and dendritic inhibitory synapses.[116,166,167] For all other m-type combinations, touches were formed between presynaptic axons, postsynaptic dendrites and somata.

The same algorithm was used to detect touches between Rt_RC dendrites, i.e. the locations of putative gap junctions. Since gap junctions are established with close appositions of cell membranes, we used a touch distance of 0 μm in this case.

At the end of this process the resulting contacts (or "appositions") are normally higher compared to experimental findings and are pruned further to arrive at the final functional synapses.[33]

Determining functional synapse positions

We employed an existing algorithm to decide which appositions were to be pruned according to biological constraints.[33] The main constraints were the experimental bouton densities (number of boutons/axonal length) from 3D neuron reconstructions (n = 9 TC axons and n = 2 Rt axons) and the coefficient of variation of number of synapses per connections (i.e. the number of functional synapses, between a pair of neurons) from presynaptic INs and post-synaptic INs and TCs.[36]

In the first two steps, the algorithm tried to match the distribution of synapses per connection, using the coefficient of variation of appositions per connections and the coefficient of variation of synapses per connection. Then, in step 3, it compared the current

bouton density to the target value and removed multi-synaptic connections until the target value was matched. The number of synapses per connections, N_{func}, was predicted from the number of appositions per connections (N_{app}) resulting from the previous steps, similarly to uncharacterized pathways in cortical microcircuitry.[33] N_{func} was predicted from N_{app} according to a simple formula ($N_{func} = 1 \cdot N_{app}$) for each m-type to m-type connection. We used a generalized coefficient of variation for N_{func} of 0.9 for all connections, extracted from published data (Morgan and Lichtman,[36] Figure 2E). The coefficient of variation was combined with the predicted N_{func} to calculate its standard deviation, as detailed in Reimann et al.[33] At the end of this pruning process, we verified that the bouton densities in the model matched the experimental ones (see Figure 2A). The shape of a geometric distribution for N_{func} was a prediction from our touch detection process.

Connections from lemniscal and corticothalamic afferents

We followed an approach similar to the generation of thalamic input to the cortical microcircuit model[24] to model afferent synapses in the thalamus from the sensory periphery (medial lemniscus) and from the cortex. The algorithm uses volumetric bouton densities and the morphologies already placed in a circuit to map synapses from afferent "virtual" fibers to postsynaptic morphologies.

We built medial lemniscus (ML) and corticothalamic (CT) afferents separately for one microcircuit. Since data for lemniscal innervation in the mouse VPL was not available we calculated volumetric bouton density from data of mouse VPM,[38] see Figure 2D for the exact values. Volumetric bouton densities for the CT pathway were derived from known proportions between CT synapses and other synapses onto TC and Rt neurons, as found in electron microscope investigations.[39,40]

Each synapse was assigned a virtual ML or CT fiber. We estimated a number of 2,601 ML fibers; this number took into account the ratio between the putative number of neurons from the dorsal column nuclei projecting to the thalamus[168] and the number of neurons in the VPL (see Jones[2] for a similar calculation). The number of CT fibers was 75,325, about ten times the number of thalamocortical fibers in a microcircuit.[64,169,170]

To take into account the correlation between synaptic inputs onto postsynaptic neurons innervated from the same afferent fiber, the mapping between postsynaptic synapses and fibers took into account their respective positions, i.e. synapses that were closer together were more likely to be innervated by the same presynaptic fiber. As in the neocortical microcircuit model,[24] the probability (P) that a synapse was assigned to a fiber depended on the distance between the synapse and the fiber:

$$P(S_{pre} = i) \propto e^{-\frac{|f_i - T_{pre}|}{2\sigma^2}}$$

where S_{pre} represents the mapping of a synapse S to the presynaptic fiber i, T_{pre} is its spatial location, f_i the spatial location of fiber i and σ denoted the degree of spatial mapping, that was set to 25 μm.

Modeling synapse physiology

Stochastic synaptic transmission and short-term plasticity

We used available paired-recording data and generalization principles to assign synaptic conductance values (g_{syn}) to match experimentally recorded PSP amplitudes. We predicted that single g_{syn} from inhibitory neurons are in general small (e.g. 0.9 ± 0.23 nS for VPL_IN to VPL_IN), while conductances from VPL_TCs and lemniscal afferents are larger (>2 nS), consistent with being "driver" synapses,[171,172] while corticothalamic synapses have small g_{syn} (<0.5 nS), but are facilitating.

To model synapse kinetics, we used existing models of synaptic currents[24] and included literature findings on decay time constants, reversal potentials and the relative contribution of AMPA, NMDA, $GABA_A$ and $GABA_B$ currents, summarized in Table S1.[118,173–176]

These models consist of a 2-state Markov process, with recovered and unrecovered states. When a pre-synaptic event occurs (pre-synaptic spike or spontaneous release) the synapse will release if it is the recovered state. If there is release, the synapse will transition to the unrecovered state. The ensemble average response is equivalent to the phenomenological Tsodyks-Markram model.[42,177] The underlying assumptions were derived from the classical model of quantal synaptic release, in which each synapse is assumed to have N independent release sites, each has a probability p of releasing a single quantum q.[178,179] The number of release sites was assumed to be equivalent to the number of synapses per connection.[24] The detailed implementation of the synapse models can be downloaded from the neuron model packages in the Neocortical Microcircuit Portal[180] (rrid:SCR_022032).

We modeled short-term synapse plasticity with depressing (E2 and I2) and facilitating synapses (E1), see Figure 2H. In our experimental recordings, in agreement with experimental findings, all existing intrathalamic (between TC, Rt neurons and INs) and lemniscal connections were depressing,[27,171,181–183] while corticothalamic ones were facilitating.[64,183–186] When sufficient experimental paired recordings data were available, the parameters of the Tsodyks-Markram model of short-term synaptic plasticity were fitted (see above). The data used for fitting were the excitatory postsynaptic potentials (EPSPs) or inhibitory postsynaptic potentials (IPSPs) peaks amplitudes (or EPSCs/IPSCs in the case of voltage-clamp recordings), evoked by stimulating the presynaptic cell with a train of eight pulses followed by a recovery pulse (see Figure 2G). The parameters were: U - release probability, D - time constant of recovery from depression, and F - time constant of recovery from facilitation. Postsynaptic data were filtered and deconvolved for easier automatic identification of the peaks.[187] A multi-objective optimization algorithm was used to find the values for U, D and F.[157]

Data to fit the UDF parameters was available for some of the pathways: Rt neurons to TCs, IN to TCs, INs to INs and ML to INs connections; for all the other pathways we followed these generalization rules:

- TC to Rt synapses were shown to be strong, reliable and depressing.[182] We used parameters for L4Exc to L4Exc connections from the neocortical microcircuit model[24] as they had the highest release probability (analogous to the U value in the case of depressing synapses.[188]
- All uncharacterized inhibitory to inhibitory synapses (i.e. Rt to Rt and Rt to IN) had the same dynamics of an inhibitory-inhibitory characterized pathway (i.e. IN to IN).
- CT synapses onto first order thalamic nuclei (e.g. VPL, VPM, dorsal part of the lateral geniculate complex) have been consistently reported to be facilitating. As we did not have paired recordings to estimate synapse parameters for CT to TCs, CT to INs and CT to Rt pathways, we took parameters from excitatory facilitating synapses (E1: L5TTPC-L5MC, Markram et al.[24])
- ML inputs to first order sensory thalamic nuclei (e.g. VPM) were shown to be depressing,[59,171,183,186] as shown in our ML to INs recordings. We thus extrapolated the parameters for ML to TC connections from ML to INs ones (for which data was available).

Synapse dynamic parameters in the model were different for each synapse and drawn for truncated Gaussian distributions.

Spontaneous miniature potentials were modeled as independent Poisson processes at each synapse that triggered release at low rates (0.01 Hz).

Synapse models

Excitatory synaptic transmission was modeled with AMPA and NMDA receptor kinetics, and $GABA_A$ receptors were used for inhibitory connections. The rise and decay phases of the currents were described using mono-exponential functions. We used time constants from thalamic experiments performed at 34–35°C, when available, or from cortical synapses models when thalamic-specific ones were missing. The rise time and decay time constants for AMPA receptors were 0.2 ms and 1.74 ms, respectively.[189] For TC to Rt connections the AMPA decay time constant was 1.58 ms and CT afferents to Rt was 2.74 ms.[190] The rise and decay time constants of the NMDA component were 0.29 and 43 ms.[191] The magnesium concentration was set to 1 mM[192] and the reversal potential of the AMPA and NMDA currents was 0 mV. Experimentally measured ratios of NMDA and AMPA conductances were gathered from the literature and are summarized in Table S1.[173,174,190]

Inhibitory synaptic transmission was modeled with $GABA_A$ receptor kinetics. The rise and decay time constants were 0.2 ms and 8.3 ms, respectively.[24] The reversal potential of $GABA_A$ current was set to −82 mV for all inhibitory pathways, except for connections onto postsynaptic TC neurons, where it was −94 mV, consistent with lower chloride reversal potentials in TC compared to Rt neurons.[193]

Constraining synapse conductance values

Synaptic conductance values were optimized by performing *in silico* paired recordings to match the postsynaptic potential (PSP) amplitudes measured experimentally whenever data was available, similarly to other morphologically detailed models.[24,188] For each pathway, 50 neuron pairs were simulated, and each pair was recorded for 30 trials. Experimentally characterized values in rodents are summarized in Table S2. For all other pathways, we extrapolated the quantal synapse conductances from similar pathways, according to the same generalization principles applied for short-term plasticity parameters (see Table S1).

Modeling gap junctions

Along with excitatory and inhibitory chemical synapses, the microcircuit included detailed gap junction (GJ) connectivity established between the dendrites of Rt neurons. We used the same touch detection algorithm described above to find appositions between Rt neuron dendrites and somata. Since we did not have any experimental data on the number of GJs between connected neurons or the density of GJs (number of GJs per unit length of dendrite or volume), in this first draft we randomly removed a certain fraction of GJs until we matched data on neuron divergence (Figure 3A). To analyze the number of coupled neurons and their spatial properties, we reproduced the experimental protocol,[45] by analyzing a sample of 33 Rt neurons in a 90 μm vertical slice located at the center of the microcircuit.

Functionally, GJ were modeled as conductances that coupled the membrane potential of the adjacent morphological compartments (simple resistors). We predicted the value of GJ conductance for all GJs and validated their functional properties by comparing coupling coefficient values with experiments.[43–46]

Once the structural properties of GJs-coupled neurons were validated, we performed *in silico* paired recordings and measured the coupling coefficients for each pair of neurons. We found that the mean coupling coefficients in the model compared well with the experiments for GJ conductance values of 0.2 nS (nS).

After adding GJs to the circuit, the input resistance of the neurons changed. To guarantee that the electrical properties of the neurons did not change, thus changing the responses to synaptic inputs, we devised an algorithm to compensate for the change in input resistance.[48] The algorithm changed the conductance of the leak current (g_{pas}) to restore the input resistance of the neuron before adding gap junctions. This compensation resulted in a different g_{pas} value for each neuron.

Simulation methods and conditions

Simulation software and high-performance computing resources

The reconstructed microcircuit was simulated using software based on the NEURON simulation package[194] (RRID:SCR_005393). A collection of tools and templates were written in order to handle simulation configuration, *in silico* network experiments and to save the results. We used the CoreNEURON simulator engine,[195] which has been optimized for efficient large-scale simulations. A typical simulation run of a microcircuit for 3,500 ms of simulation time took ~45 min on 16 Intel Xeon 6140 CPUs (288 cores, with HyperThreading enabled).

Simulating in vivo-like conditions

To simulate spontaneous activity in *in vivo* wakefulness-like states, we activated lemniscal and CT fibers with Poisson spike trains at 25 and 4 Hz, respectively. To find the optimal value of lemniscal and cortical spontaneous rates we started from literature values[196,197] and then explored different combinations until we reached a biologically-plausible level of spontaneous activity in the model. We considered it as biologically plausible uncorrelated (asynchronous) firing in all cell types. From the literature, we know that spontaneous activity from L6 corticothalamic afferents is low in spontaneous conditions and is lower than lemniscal activity. We lowered the extracellular calcium concentration from 2 mM (*in vitro*-like conditions) to 1.2 mM, with the effect of reducing synapse release probabilities and PSPs amplitudes.[24] Since the dependence of PSP amplitudes on the extracellular Ca^{2+} concentration in the thalamus is not known, we assumed that PSPs were dependent on calcium concentration in the same way as uncharacterized pathways as previously published (Markram et al.,[24] Figure S11 – Intermediate $[Ca^{2+}]_o$ dependence). This condition was used in all simulations of *in vivo* wakefulness-like activity, if not stated otherwise.

To simulate cortical UP and DOWN states, we removed the background activity from the CT afferents (Figures 4 and S5).

Simulating lightly-anesthetized in vivo-like conditions

To simulate lightly anesthetized *in vivo*-like states, we followed the same methodology as for *in vivo*-like conditions, however the spontaneous firing induced at the thalamus through the ML afferents was reduced from 25 Hz to 10 Hz to reflect the presumed hyperpolarizing influence of the anesthetic (Figure 4). After exploring different values for the ML background activity, we found that for the 10 Hz ML background TC cells responded to the stimulus with low-threshold spikes of bursts as shown in the related publication.[63]

Simulating in vitro-like conditions

To simulate *in vitro*-like states, all neurons were left at their resting potentials (which ranged between −75 and −70 mV as recorded experimentally) and the only source of input was the spontaneous synaptic release from intrathalamic, medial lemniscus and corticothalamic synapses (at a rate of 0.01 Hz). The spontaneous synaptic release rate was taken from other modeling studies. After verifying that changing this value had no significant effect on the microcircuit network activity, we did not modify it in any simulation condition. The extracellular calcium concentration was set to 2 mM, as used in our *in-vitro* experiments.

Simulating depolarization levels

As a first approximation of the action of neuromodulators in the VPL and Rt, we applied constant current injections to the soma of each neuron. All neurons in the VPL or Rt regions were depolarized to the same target baseline membrane potential. The amplitude of the current was different for each neuron, to take into account the different input resistance of each morpho-electrical model.

Simulation analysis

The spectrogram in Figure S4 was calculated using the function *scipy.signal.spectrogram*, with inputs the sampling frequency of simulated membrane potential (10 kHz), *interval* = 5000, *overlap* = 0.99, and the other parameters with the default values.

Burst probabilities (Figure S4) were calculated as the ratio between the numbers of spikes belonging to a burst and the overall number of spikes. We considered a spike belonging to a burst when the interspike intervals were $\leq$ 15 ms and the first spike in the burst was preceded by a pause $\geq$ 50ms. For this analysis, we considered neurons that had a baseline activity between 1 and 20 Hz, as shown in corresponding publication.[74]

To analyze the percentage of neurons firing for each m-type during each cycle of the oscillation (Figure 6C) we started by finding the oscillation peaks. The peaks were extracted from the firing rate histograms as input, using the *scipy.signal.find_peaks* function. We then added a peak corresponding to the time of the stimulus injected into Rt neurons (cycle 0). Spikes for each m-type were then assigned to the different cycles if they occurred within 30 ms of the oscillation peak.

Oscillation strength (Figure 7) was calculated as the maximal value of the power spectral density (PSD). The PSD was obtained using the function *scipy.signal.periodogram*.

To calculate the oscillation duration (in ms) we used firing rate histograms for all the neurons and extracted their peaks, using the *scipy.signal.find_peaks* function (Figure 7). Peaks were counted only if they were significantly higher than baseline firing rates. Oscillation duration was then calculated as the time difference between the last and the first peak.

To calculate oscillation frequency (Figure 7), we computed the normalized autocorrelation of the firing rate histograms and extracted the time (oscillation period) corresponding to the first non-zero peak.[73] The inverse of the oscillation period corresponded to the oscillation frequency.

Cell Reports, Volume *42*

Supplemental information

Thalamic control of sensory processing and spindles in a biophysical somatosensory thalamoreticular circuit model of wakefulness and sleep

Elisabetta Iavarone, Jane Simko, Ying Shi, Marine Bertschy, María García-Amado, Polina Litvak, Anna-Kristin Kaufmann, Christian O'Reilly, Oren Amsalem, Marwan Abdellah, Grigori Chevtchenko, Benoît Coste, Jean-Denis Courcol, András Ecker, Cyrille Favreau, Adrien Christian Fleury, Werner Van Geit, Michael Gevaert, Nadir Román Guerrero, Joni Herttuainen, Genrich Ivaska, Samuel Kerrien, James G. King, Pramod Kumbhar, Patrycja Lurie, Ioannis Magkanaris, Vignayanandam Ravindernath Muddapu, Jayakrishnan Nair, Fernando L. Pereira, Rodrigo Perin, Fabien Petitjean, Rajnish Ranjan, Michael Reimann, Liviu Soltuzu, Mohameth François Sy, M. Anıl Tuncel, Alexander Ulbrich, Matthias Wolf, Francisco Clascá, Henry Markram, and Sean L. Hill

Table S1

Pathway	Synapse type	g_{syn} (nS)	τ_d (ms)	NMDA/AMPA ratio	U_{SE}	D	F
**Rt_RC to Rt_RC**	Inh. Dep.	0.9±0.23	8.3±2.2	NA	0.41±0.14	464±339	54±71
**Rt_RC to VPL_TC**	Inh. Dep.	1.1±0.4	8.3±2.2	NA	0.32±0.18	352±46	2±209
**Rt_RC to VPL_IN**	Inh. Dep.	0.9±0.23	8.3±2.2	NA	0.41±0.14	464±339	54±71
**VPL_TC to Rt_RC**	Exc. Dep.	2.8±0.1	1.58±0.26	0.57	0.86±0.09	671±17	17±5
**VPL_IN to VPL_TC**	Inh. Dep.	0.4±0.4	8.3±2.2	NA	0.47±0.18	137±46	239±209
**VPL_IN to VPL_IN**	Inh. Dep.	2.7±0.4	8.3±2.2	NA	0.41±0.14	464±339	54±71
**ML to VPL_TC**	Exc. Dep.	1.15±0.12	1.74±0.18	0.41	0.3±0.21	2350±315	1±2
**ML to VPL_IN**	Exc. Dep.	1.15±0.12	1.74±0.18	0.41	0.48±0.21	690±315	57±53
**CT to Rt_RC**	Exc. Fac.	0.16±0.016	2.74±0.25	0.99	0.09±0.12	138±211	670±830
**CT to VPL_TC**	Exc. Fac.	0.16±0.016	1.74±0.18	1.91	0.09±0.12	138±211	670±830
**CT to VPL_IN**	Exc. Fac.	0.16±0.016	1.74±0.18	0.99	0.09±0.12	138±211	670±830

Table S1. Synapse kinetics and short-term plasticity parameters, related to Figure 2

Synaptic parameters for all pathways in the model. Quantal synaptic conductance g_{syn} (in nanosiemens nS), τ_d is the decay time constant of AMPA and $GABA_A$ currents for excitatory and inhibitory connections. U_{SE} (utilisation of synaptic efficacy, analogous to release probability), D (time constant of recovery from depression), F (time constant of recovery from facilitation) are the short-term plasticity parameters. Values are expressed as mean ± standard deviation. All the parameters were fitted to in-house paired-recordings or generalized from similar pathways.

Table S2

**Presynaptic**	**Postsynaptic**	**CV 1st PSP amplitude, experiment (mV)**	**CV 1st PSP amplitude, model (mV)**	**Data source**
**Rt_RC**	**VPL_TC**	0.4600 (n=1)	0.8424 ± 0.3450 (n=47)	In-house
**VPL_TC**	**Rt_RC**	0.1232 ± 0.0686 (n=11)	0.3089 ± 0.2112 (n=43)	(Gentet and Ulrich, 2003)
**VPL_IN**	**VPL_TC**	0.5479 ± 0.1744 (n=4)	0.8663 ± 0.386 (n=22)	In-house
**VPL_IN**	**VPL_IN**	0.5028 ± 0.2783 (n=10)	1.0993 ± 0.4132 (n=49)	In-house
**ML**	**VPL_IN**	0.5466 ± 0.1195 (n=1)	1.4047 ± 0.7389 (n=49)	In-house

TableS 2. Coefficient of variation (CV) of first PSP amplitudes, related to Figure 2

CV of first PSP amplitudes values as characterized experimentally through *in vitro* paired recordings. Values are reported as mean ± standard deviation (of multiple pairs). (Related to Fig. 5C1).

Table S3

Presynaptic	Postsynaptic	PSP amplitude, experiment (mV)	PSP amplitude, model (mV)	Data source
**Rt_RC**	**VPL_TC**	1.33 ± 0.36 (n=1)	1.31 ± 1.30 (n=47)	In-house
**VPL_TC**	**Rt_RC**	7.4 ± 1.5 (n=11)	6.79 ± 1.30 (n=43)	(Gentet and Ulrich, 2003)
**VPL_IN**	**VPL_TC**	0.55±0.15 (n=4)	1.16 ± 1.56 (n=22)	In-house
**VPL_IN**	**VPL_IN**	1.66±1.44 (n=10)	0.82 ± 0.77 (n=49)	In-house
**ML**	**VPL_TC**	4.58 ± 0.30 (n=11)	3.62 ± 2.30 (n=49)	(Mo et al., 2017)
**CT**	**VPL_TC**	0.085±0.008 (n=3)	0.071 ± 0.022 (n=29)	(Golshani et al., 2001)

Table S3. Postsynaptic potential (PSP) amplitudes, related to Figure 2

PSP amplitude values as characterized experimentally through *in vitro* paired recordings. Values are reported as mean ± standard deviation (of multiple pairs).

Figure S1

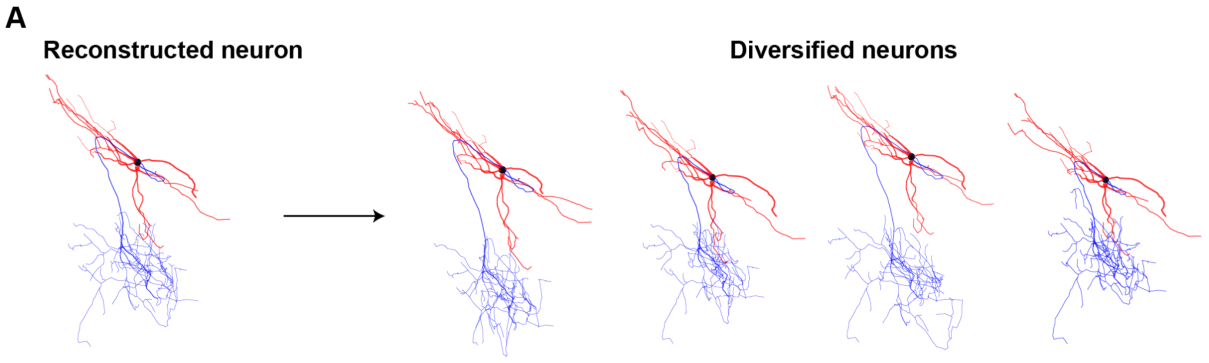

Figure S1, morphology diversification, related to Figure 1.

(A) The reconstructed neuron on the left was diversified to generate a sample of unique morphologies, by introducing variability (jittering in f branch lengths of 0 ± 20% and jittering in branch rotations of 0° ± 20°, mean ± standard deviation, see Methods) to the branch lengths and angles (see Methods).

Figure S2

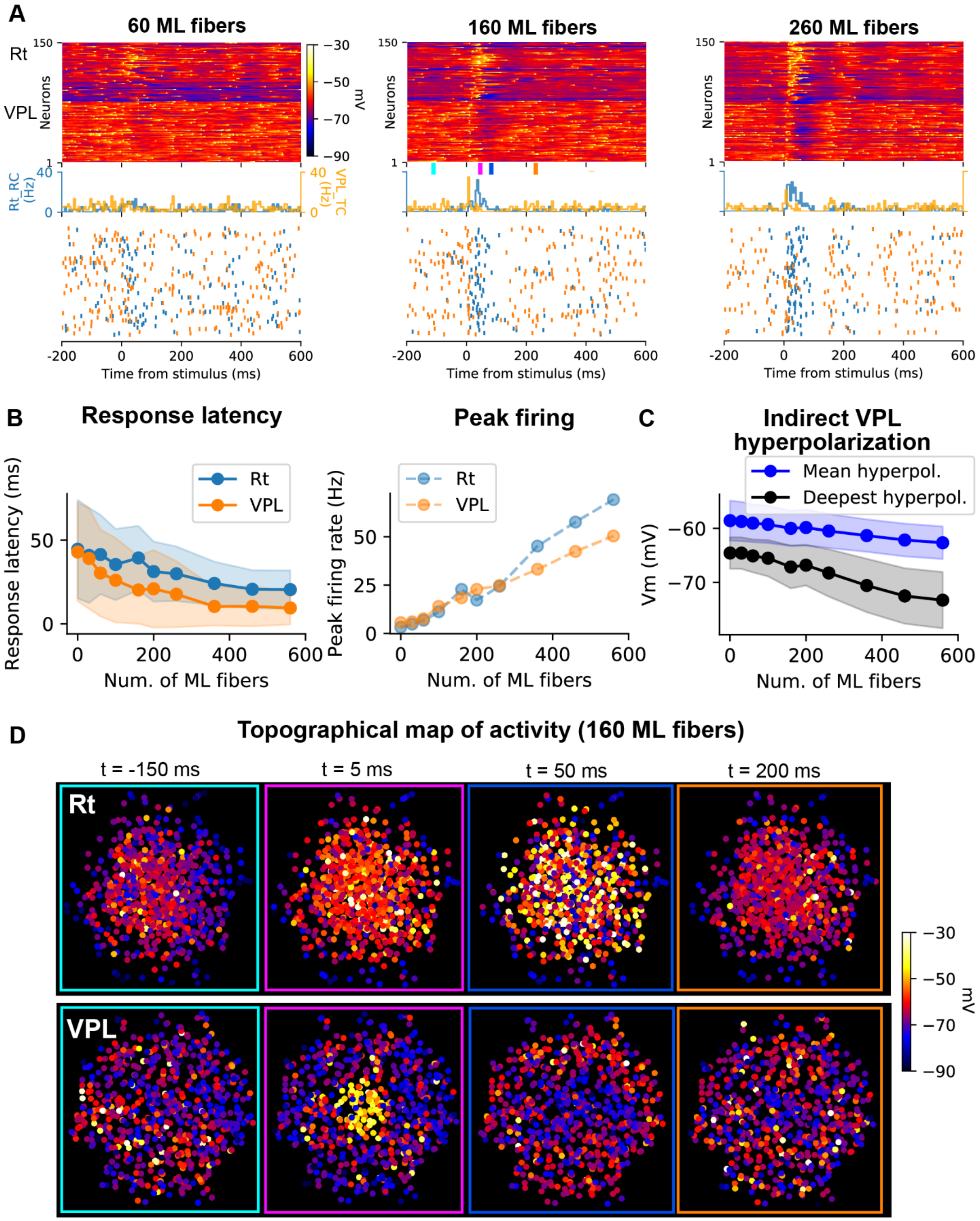

A
60 ML fibers
160 ML fibers
260 ML fibers
Rt
VPL
Neurons
mV
Time from stimulus (ms)
B
Response latency
Response latency (ms)
Num. of ML fibers
Rt
VPL
Peak firing
Peak firing rate (Hz)
C
Indirect VPL hyperpolarization
Mean hyperpol.
Deepest hyperpol.
Vm (mV)
D
Topographical map of activity (160 ML fibers)
t = -150 ms
t = 5 ms
t = 50 ms
t = 200 ms
Rt
VPL
mV

Figure S2. **Stimulus-dependent recruitment of reticular nucleus neurons and surround inhibition of thalamic neurons (*in vivo* wakefulness-like condition), related to Figure 4.**

(**A**) Simulated sensory inputs with brief activation of increasing numbers of medial lemniscal fibers (ML). Top: voltage rasters show Rt and VPL responses of a sample of 150 active neurons, sorted by their vertical position in the microcircuit. Bottom: spiking responses (firing rate histograms and spike rasters). Note that stimulus-evoked responses in the VPL as well as the following hyperpolarization increase with increasing stimulus size. (**B**) Stimulus-response curves. Response latency decreases with stimulus size (left), while peak firing rates increase the VPL, as well as in the Rt (right). Mean (lines) and standard deviation (shaded areas) are shown. The peak firing rate is calculated in the 100 ms following the stimulus and response latency as the time to first spike after the stimulus (n=1,000 neurons). (**C**) Stimulus-dependent hyperpolarization in the VPL. The mean and deepest hyperpolarization in VPL cells are shown (lines: mean, shaded areas: standard deviation). Note that with increasing stimulus size more VPL cells are inhibited by Rt neurons and the hyperpolarization becomes stronger. The hyperpolarization is calculated in a time window of 40-200 ms after the stimulus (same sample as in B, n=1,000). (**D**) Topographical activity in a slice through the VPL and Rt showing the average membrane potentials at different time windows before, during and after the stimulus, as indicated by colored ticks in A (middle panel), 160 fibers were activated. Time windows of 10 ms starting at the time indicated were used for the average activity. Note that increased activity is confined to the central part of the VPL in response to the stimulus (t=5 ms), which triggers spiking activity in the Rt (t=50 ms) in central as well as in peripheral neurons. This result suggests that the Rt has larger receptive fields compared to the VPL. Consequently (t=50ms), the central part and the surround in the VPL is inhibited (blue points at t=50ms).

Figure S3

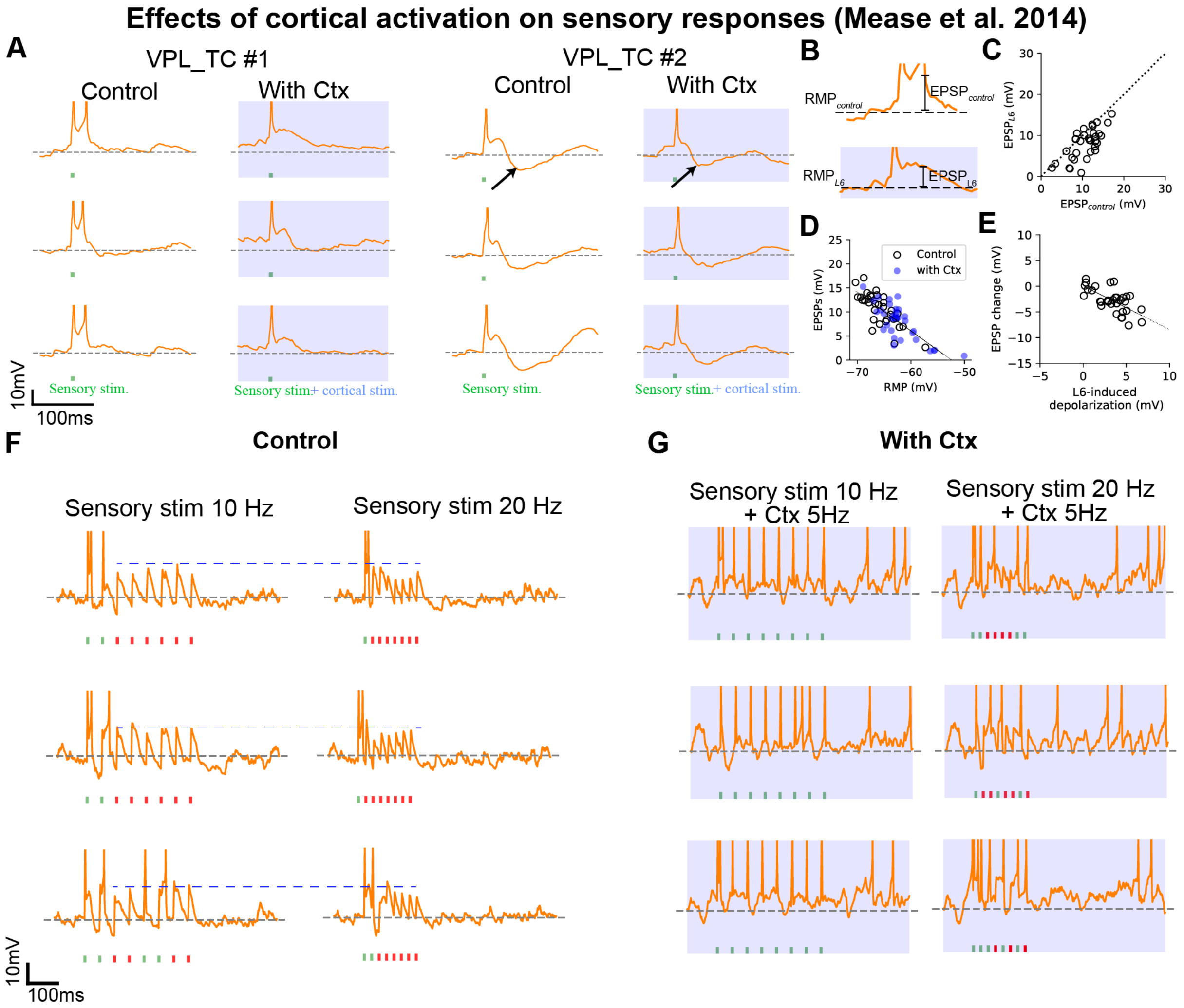
Effects of cortical activation on sensory responses (Mease et al. 2014)
A
VPL_TC #1
Control
With Ctx
VPL_TC #2
Control
With Ctx
10mV
100ms
Sensory stim.
Sensory stim.+ cortical stim.
Sensory stim.
Sensory stim.+ cortical stim.
B
RMP control
EPSP control
RMP L6
EPSP L6
C
EPSP L6 (mV)
EPSP control (mV)
D
Control
with Ctx
EPSPs (mV)
RMP (mV)
E
EPSP change (mV)
L6-induced depolarization (mV)
F
Control
Sensory stim 10 Hz
Sensory stim 20 Hz
G
With Ctx
Sensory stim 10 Hz + Ctx 5Hz
Sensory stim 20 Hz + Ctx 5Hz
10mV
100ms

Figure S3. Cortical activation decreases sensory adaptation by depolarizing VPL_TCs and enhances responses to stimuli at ~10Hz preferentially, related to Figure 5.

(**A**) Left: Single cell recording of a VPL_TC neuron (3 of 25 repetitions are shown) that responded to the sensory stimulus (green) with a burst of two spikes. The sensory stimulus was generated with brief synchronous activation of 160 ML afferent fibers. Right: activation of the CT afferent fibers (blue) stimulus depolarized the cells and shifted their responses to single spikes. VPL_TC #2 showed a marked IPSP (arrow) following the stimulus-evoked spike, which was reduced with cortical activation. CT fibers were activated with noisy input at 4 Hz, 200 ms before the sensory stimulus to approximate the optogenetic protocol in Mease et al., 2014. (**B**) Illustration of different metrics used to quantify subthreshold responses (in a time window of 50 ms after the stimulus to the sensory stimuli (cfr. Mease et al, 2014). (**C**) Population analysis of VPL_TC cells (n=50, values are median of the 25 repetitions for each cell) showing the decrease of EPSP amplitude with cortical activation ($EPSP_{L6}$). This effect is due to partial inactivation of the low-threshold Ca^{2+} conductance, but inhibition from the Rt can't be excluded. (**D**) The amplitude of the EPSPs (both with and without cortical activation) is negatively correlated with the resting potential of the cell (r=−0.8). This is due to a greater availability of ionic currents activated at hyperpolarized potentials and greater driving force of excitatory conductances (whose reversal potential is 0 mV). (**E**) Correlation between the magnitude of sensory response change ($EPSP_{L6}$ − EPSP with sensory stimulus only) and the depolarization induced by the cortical activation. The line shows the best fit (r=−0.8). This shows that greater cortical activation corresponds to decreased responses to sensory input (for single stimulation). (**F**) Single cell recordings of a VPL_TC neuron (3 of 25 repetitions are shown) with sensory stimulus at 10 Hz (left) and 20 Hz (right). Note the smaller amplitudes of the EPSPs in response to the 20 Hz stimulus. (**G**) Same cell as in F, with activation of cortex (noisy input at 5 Hz). Note a higher number of spiking failures (red ticks) with 20 Hz sensory stimulation. With 10 Hz stimulus, cortical activation made the cell fire in response to each pulse of the stimulus (green ticks).

Figure S4

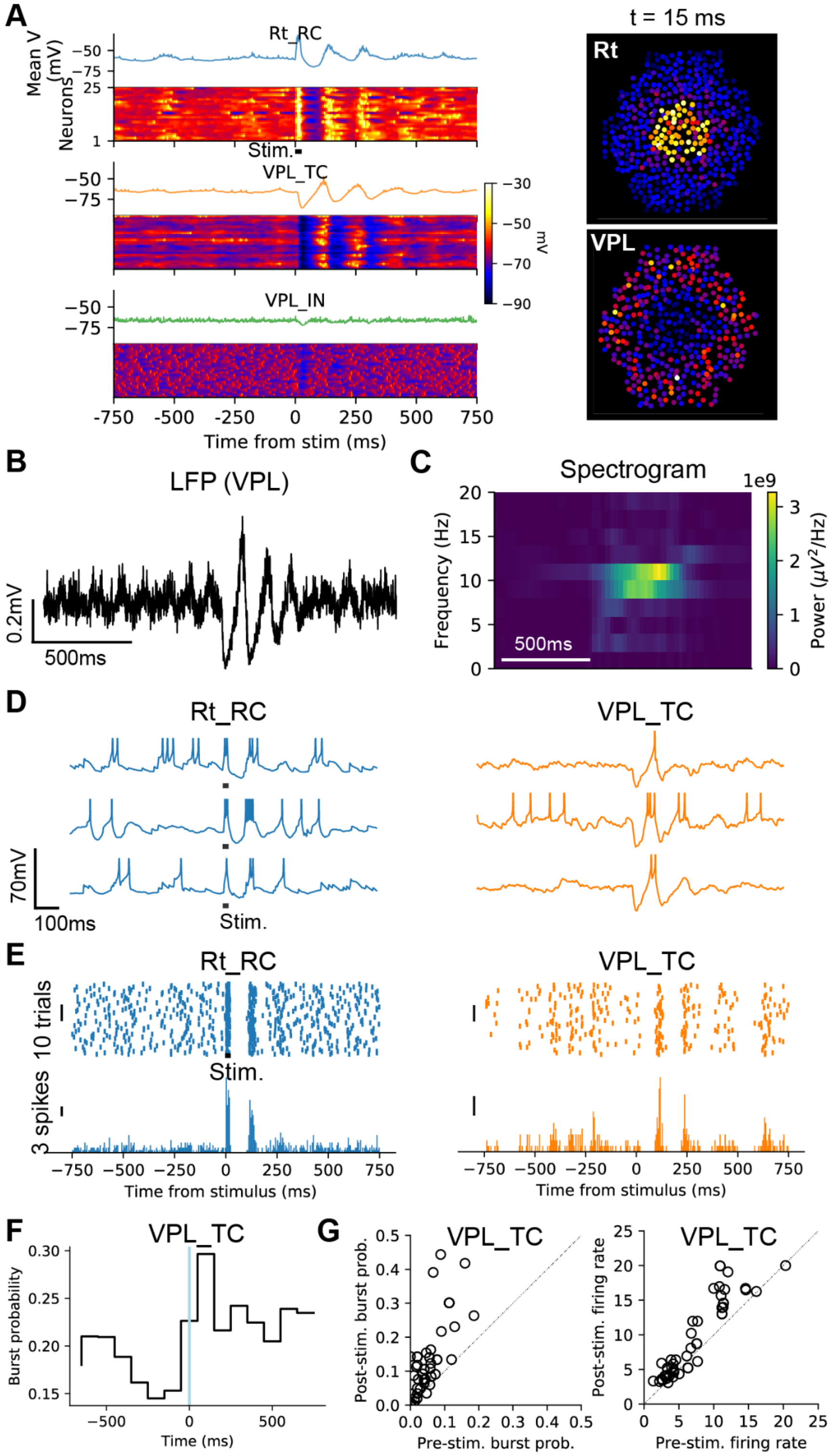
A
Rt_RC
Mean V (mV)
Neurons
Stim.
VPL_TC
VPL_IN
mV
Time from stim (ms)
t = 15 ms
Rt
VPL
B
LFP (VPL)
0.2mV
500ms
C
Spectrogram
1e9
Frequency (Hz)
Power (μV^2/Hz)
500ms
D
Rt_RC
VPL_TC
70mV
100ms
Stim.
E
Rt_RC
VPL_TC
10 trials
3 spikes
Stim.
Time from stimulus (ms)
F
VPL_TC
Burst probability
Time (ms)
G
VPL_TC
Post-stim. burst prob.
Pre-stim. burst prob.
VPL_TC
Post-stim. firing rate
Pre-stim. firing rate

Figure S4. Activating the reticular nucleus increases thalamic bursts and initiates spindle-like oscillations (*in vivo* wakefulness-like condition), related to Figure 6.

Spindle-like oscillations are evoked by localized pulse (20 ms) activation of 750 Rt_RC cells located at the center of the microcircuit. (**A**) Left: voltage rasters showing spindle-like activity. A sample of neighboring 25 neurons per each m-type is shown and color-coded according to their membrane potential. Right: topographical map of activity showing average membrane potential of Rt and VPL neurons in a 10 ms time window starting at the time indicated. (**B**) LFP recording from a central site in the VPL. Note the increased oscillatory activity after the stimulus applied to the Rt (not shown here). (**C**) Frequency-time analysis of the LFP in C showing increased power in the 8-10 Hz frequency range. (**D**) Example of single cell recordings for 3 cells per m-type. Note the burst responses in Rt_RCs and the IPSPs-rebound sequences in VPL_TCs. (**E**) Left: spike rasters and PSTHs showing the activity of one exemplar Rt_RC neuron (50 trials). Note the increased activity in response to the stimulus (black dot, 20 ms pulse) and a second peak, generated by network interactions. Right: Same as in A, for one example VPL_TC, note the post-inhibitory rebound response ~100 ms after the Rt stimulation. (**F**) Histogram showing increased burst probability following the stimulus in VPL_TC (n=100 VPL_TC), as shown in experiments (Halassa et al., 2011). (**G**) Left: burst probability in VPL_TCs increases as a result of Rt_RC stimulus (each dot corresponds to one cell, the same sample as in F, n=100). Right: analysis of firing rates of VPL_TCs before and after the stimulus, as shown in experiments (Halassa et al., 2011). Pre-stim./post-stim. data were calculated in the 1s preceding/following the stimulus

Figure S5

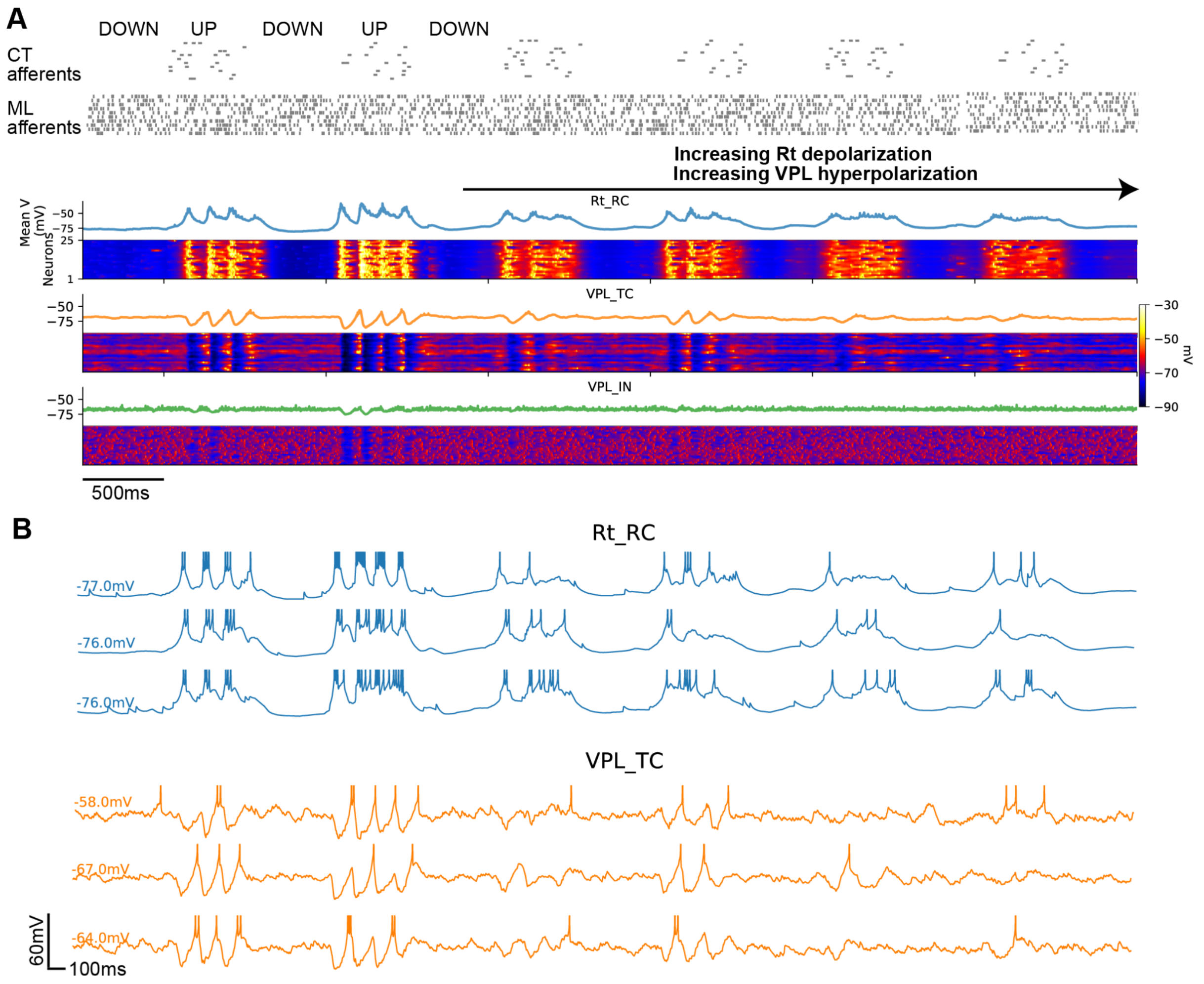

Figure S5. Spindle-like oscillations cease when simulating the effect of neuromodulation on thalamic and reticular neurons, related to Figure 7.

(**A**) Simulated UP and DOWN states evoke reticular and thalamic depolarizations through afferent input, resulting in the "ping-pong" generation of spindle-like oscillations (as seen in Fig. 11). To approximate the differential effects of neuromodulators (e.g. acetylcholine) onto Rt_RC and VPL_TC we applied constant currents to depolarize Rt_RC and hyperpolarize VPL_TC cells. This resulted in spindle-like oscillations (left) being abolished (right). (**B**) Example single cell recordings from the simulation in A, note that while Rt_RC cells fire preferentially low threshold bursts during the cortical UP states (left), they transition to single spike modes when depolarized (right). The change in Rt_RC firing mode and hyperpolarization of VPL_TC cells resulted in a significant decrease of large amplitude IPSPs in VPL_TC cells and rebound bursts.
